# Supplementary material for: Cost-effectiveness of COVID rapid diagnostic tests for patients with severe/critical illness in low- and middle-income countries: A modeling study
Source: PLoS Med. 2024 Jul 18;21(7):e1004429. doi: 10.1371/journal.pmed.1004429 (PMC11293649; doi:10.1371/journal.pmed.1004429)
Supplement: S1 Appendix — (DOCX) [file pmed.1004429.s001.docx]

# **S1 Appendix: Context and checklists**

## **A. Rapid review of the evidence before this study**

We searched Embase and Medline for papers published between 1 Jan. 2020 and 5 Dec. 2023 on the cost-effectiveness, cost-utility or cost-benefit of COVID testing. We searched for: *(cost-effectiveness OR cost-utility OR cost-benefit OR (cost adj analysis)) AND (test* OR PCR OR RDT OR antigen) AND (COVID* OR SARS-CoV-2 OR coronavirus)* in paper titles or abstracts. We also required the title or abstract or (for Embase) MeSH term to contain the name of a low-and-middle-income country (as per the World Bank’s 2022 classification [1]) or one of the following terms: *Africa OR Asia OR Latin OR LMICs OR low-income OR middle-income*. We included papers in English, French, Spanish and Portuguese, and excluded protocols, conference abstracts and papers computing neither a net monetary benefit nor an incremental cost-effectiveness, cost-benefit or cost-utility ratio.

100 records were initially identified, but 29 of them were excluded as they did not focus on SARS-CoV-2, 40 because they were not cost-effectiveness analyses, 8 because they did not actually consider testing. Out of the remaining 23 records, 3 were conference abstracts, one was not focusing on LMICs, and 2 were reviews in Chinese of the literature worldwide (given our lack of knowledge of the language, it was unclear whether these reviews included LMIC papers, and which ones). This left seventeen papers that met our criteria, including two literature reviews [2,3], two preprints [4,5], and 13 journal articles [6–18]. 6/15 papers [4,5,7–9,17] (excluding the reviews) referred to antigen-based rapid diagnostic tests (Ag-RDTs). Only a minority (6/15 [5,6,8,10,12,13], and 2/6 papers on Ag-RDTs [5,8]) considered testing for improved treatment. Furthermore, two of those [8,10] (one of them [8] on ag-RDTs) did not include any measure of health outcomes, and only two [5,10] examined different SARS-CoV-2 prevalences. Of the two papers on Ag-RDTs that considered testing for treatment, one was a journal article that compared testing costs to numbers diagnosed accurately [8] in Brazil, while the other was a preprint comparing DALYs, testing and treatment costs [5] in Kenya. It compared three options (clinical judgement, delayed polymerase chain reaction - PCR, or ag-RDTs) applied to people presenting to care (suspected cases or asymptomatic contacts) at 5%, 10% or 20% prevalence and found that the use of Ag-RDTs was cost-effective at all levels of prevalence when PCRs were unavailable and at high prevalence levels when they were. No paper considered both presumptive COVID treatment and symptomatic treatment as alternatives to testing, estimated the unintended effects of treating false positives, or assessed a range of treatment options (e.g. corticosteroids and/or IL-6 receptor blockers).

In this context, we felt that our paper could meaningfully add to the extremely scarce literature on COVID testing in support of treatment and care in low-resource settings, particularly through the inclusion of a large range of countries and scenarios, and by including for the first time unintended treatment effects and their health and economic consequences.

## **B. CHEERS 2022 checklist**

**S1 Table: CHEERS 2022 reporting checklist**

| **Topic** | **No.** | **Item** | **Location where item is reported** |
| --- | --- | --- | --- |
| **Title and abstract** | | | |
| Title | 1 | Identify the study as an economic evaluation and specify the interventions being compared. | See Title on the first page |
| Abstract | 2 | Provide a structured summary that highlights context, key methods, results, and alternative analyses. | See Abstract on the first page |
| **Introduction** | | | |
| Background and objectives | 3 | Give the context for the study, the study question, and its practical relevance for decision making in policy or practice. | See introduction section (para 3 for the study question, para 2 for relevance) |
| **Methods** | | | |
| Health economic analysis plan | 4 | Indicate whether a health economic analysis plan was developed and where available. | Not applicable as not RCT based |
| Study population | 5 | Describe characteristics of the study population (such as age range, demographics, socioeconomic, or clinical characteristics). | See “model overview” section |
| Setting and location | 6 | Provide relevant contextual information that may influence findings. | See “country context” section |
| Comparators | 7 | Describe the interventions or strategies being compared and why chosen. | See “testing options and implications” section, paras 1 and 2 |
| Perspective | 8 | State the perspective(s) adopted by the study and why chosen. | See “costs” section |
| Time horizon | 9 | State the time horizon for the study and why appropriate. | See the first paragraph of Methods |
| Discount rate | 10 | Report the discount rate(s) and reason chosen. | See “costs” and “health impact of disease and treatment” sections |
| Selection of outcomes | 11 | Describe what outcomes were used as the measure(s) of benefit(s) and harm(s). | See “costs” and “health impact of disease and treatment” sections |
| Measurement of outcomes | 12 | Describe how outcomes used to capture benefit(s) and harm(s) were measured. | See “costs” and “health impact of disease and treatment” sections |
| Valuation of outcomes | 13 | Describe the population and methods used to measure and value outcomes. | See “estimates of model parameters and cost-effectiveness” section |
| Measurement and valuation of resources and costs | 14 | Describe how costs were valued. | See “costs” section and Appendices 2.3.2 and 2.4. |
| Currency, price date, and conversion | 15 | Report the dates of the estimated resource quantities and unit costs, plus the currency and year of conversion. | See “Costs” section and Appendices 2.3.2 and 2.4. |
| Rationale and description of model | 16 | If modelling is used, describe in detail and why used. Report if the model is publicly available and where it can be accessed. | See “model overview” section and “analysis methods” section, para 3. |
| Analytics and assumptions | 17 | Describe any methods for analysing or statistically transforming data, any extrapolation methods, and approaches for validating any model used. | See “analysis methods” section (paras 1-3). |
| Characterising heterogeneity | 18 | Describe any methods used for estimating how the results of the study vary for subgroups. | Between-country heterogeneity was described in “analysis methods” para 3 & S4 Appendix. |
| Characterising distributional effects | 19 | Describe how impacts are distributed across different individuals or adjustments made to reflect priority populations. | Not applicable. |
| Characterising uncertainty | 20 | Describe methods to characterise any sources of uncertainty in the analysis. | See “sensitivity analyses” section and “analysis method” section (para 2). |
| Approach to engagement with patients and others affected by the study | 21 | Describe any approaches to engage patients or service recipients, the general public, communities, or stakeholders (such as clinicians or payers) in the design of the study. | LMIC partners were involved in this paper and are among its co-authors. See author list, “Methods and findings” Abstract section & “Country context: results of an experts’ consultation” section. |
| **Results** | | | |
| Study parameters | 22 | Report all analytic inputs (such as values, ranges, references) including uncertainty or distributional assumptions. | Study parameter values and distributions are detailed in S2 Appendix. |
| Summary of main results | 23 | Report the mean values for the main categories of costs and outcomes of interest and summarise them in the most appropriate overall measure. | Mean net monetary benefits per country group and across the range of prevalences explored in this paper are provided in Section A in S3 Appendix. |
| Effect of uncertainty | 24 | Describe how uncertainty about analytic judgments, inputs, or projections affect findings. Report the effect of choice of discount rate and time horizon, if applicable. | See Results section (throughout), and S3 Appendix. |
| Effect of engagement with patients and others affected by the study | 25 | Report on any difference patient/service recipient, general public, community, or stakeholder involvement made to the approach or findings of the study | No patients were involved in this study. LMIC stakeholders’ contributions are highlighted in the “Country context: results of an experts’ consultation” section. |
| **Discussion** | | | |
| Study findings, limitations, generalisability, and current knowledge | 26 | Report key findings, limitations, ethical or equity considerations not captured, and how these could affect patients, policy, or practice. | See Discussion section, particularly para 1 (key findings), paras 4, 6-7 (limitations) & paras 2-3 (implications for patients, policy or practice). |
| **Other relevant information** | | | |
| Source of funding | 27 | Describe how the study was funded and any role of the funder in the identification, design, conduct, and reporting of the analysis. | Sources of funding were detailed in authors’ individual declarations and funding statement. |
| Conflicts of interest | 28 | Report authors conflicts of interest according to journal or International Committee of Medical Journal Editors requirements. | Conflicts of interest forms were filled in by all authors. |

# **References**

1. World Bank Country and Lending Groups. 2022.

2. Dolatshahi Z, Nargesi S, Sadeghifar J, Mezginejad F, Jafari A, Bazyar M, et al. Economic evaluation of laboratory diagnostic test types in Covid-19 epidemic: A systematic review. International Journal of Surgery. 2022 Sep 1;105:106820.

3. Zhou L, Yan W, Li S, Yang H, Zhang X, Lu W, et al. Cost-effectiveness of interventions for the prevention and control of COVID-19: Systematic review of 85 modelling studies. J Glob Health. 2022 Jun 15;12:05022.

4. Chevalier J, Han A, Hansen M, Klock E, Pandithakoralage H, Ockhuisen T, et al. Impact and Cost-Effectiveness of SARS-CoV-2 Self-Testing Strategies in Schools: A Multi-Country Modelling Analysis. 2023.

5. Arwah B, Mbugua S, Ngure J, Makau M, Mwaura P, Kamau D, et al. Cost & Cost-Effectiveness of Implementing SD Biosensor Antigen Detecting SARs-CoV-2 Rapid Diagnostic Tests in Kenya. medRxiv. 2023 Jan 1;2023.01.05.23284225.

6. Jiang Y, Cai D, Chen D, Jiang S. The cost-effectiveness of conducting three versus two reverse transcription-polymerase chain reaction tests for diagnosing and discharging people with COVID-19: evidence from the epidemic in Wuhan, China. BMJ Global Health. 2020 Jul 1;5:e002690.

7. Li H, Zhang H. Cost-effectiveness analysis of COVID-19 screening strategy under China’s dynamic zero-case policy. Frontiers in Public Health [Internet]. 2023;11. Available from: https://www.frontiersin.org/articles/10.3389/fpubh.2023.1099116

8. Cedro VQM, de Lima Gomes S, Simões ACCD, Sverzut T do VL, Bertti KCX, Tristão MT, et al. Cost-effectiveness analysis of COVID-19 tests in the unified health system. Cost Eff Resour Alloc. 2023 Sep 13;21(1):64.

9. Aldila D. Analyzing the impact of the media campaign and rapid testing for COVID-19 as an optimal control problem in East Java, Indonesia. Chaos, Solitons & Fractals. 2020 Dec 1;141:110364.

10. Rahmanzadeh F, Malekpour N, Faramarzi A, Yusefzadeh H. Cost-effectiveness analysis of diagnostic strategies for COVID-19 in Iran. BMC Health Serv Res. 2023 Aug 14;23(1):861.

11. de Assis TSM, Freire ML, Carvalho J de P, Rabello A, Cota G. Cost-effectiveness of anti-SARS-CoV-2 antibody diagnostic tests in Brazil. PLOS ONE. 2022 Feb 25;17(2):e0264159.

12. Reddy KP, Shebl FM, Foote JHA, Harling G, Scott JA, Panella C, et al. Cost-effectiveness of public health strategies for COVID-19 epidemic control in South Africa: a microsimulation modelling study. Lancet Glob Health. 2021 Feb;9(2):e120–9.

13. Guzmán Ruiz Y, Vecino-Ortiz AI, Guzman-Tordecilla N, Peñaloza-Quintero RE, Fernández-Niño JA, Rojas-Botero M, et al. Cost-Effectiveness of the COVID-19 Test, Trace and Isolate Program in Colombia. Lancet Reg Health Am. 2022 Feb;6:100109.

14. Asamoah JKK, Owusu MA, Jin Z, Oduro FT, Abidemi A, Gyasi EO. Global stability and cost-effectiveness analysis of COVID-19 considering the impact of the environment: using data from Ghana. Chaos, Solitons & Fractals. 2020 Nov 1;140:110103.

15. Wang X, Pei S, Wang L, La B, Zhao M, Zhang X, et al. Investigation on the possibility of dynamic COVID-Zero strategy in China: a population-based transmission model analysis and economic evaluation. BMJ Open. 2023 Aug 3;13(8):e067294.

16. Bogere N, Bongomin F, Katende A, Ssebambulidde K, Ssengooba W, Ssenfuka H, et al. Performance and cost-effectiveness of a pooled testing strategy for SARS-CoV-2 using real-time polymerase chain reaction in Uganda. Int J Infect Dis. 2021 Dec;113:355–8.

17. Aldila D, Ndii MZ, Samiadji BM. Optimal control on COVID-19 eradication program in Indonesia under the effect of community awareness. Math Biosci Eng. 2020 Sep 23;17(6):6355–89.

18. Asamoah JKK, Jin Z, Sun GQ, Seidu B, Yankson E, Abidemi A, et al. Sensitivity assessment and optimal economic evaluation of a new COVID-19 compartmental epidemic model with control interventions. Chaos, Solitons & Fractals. 2021 May 1;146:110885.
