# Supplementary material for: Cost-effectiveness of COVID rapid diagnostic tests for patients with severe/critical illness in low- and middle-income countries: A modeling study
Source: PLoS Med. 2024 Jul 18;21(7):e1004429. doi: 10.1371/journal.pmed.1004429 (PMC11293649; doi:10.1371/journal.pmed.1004429)
Supplement: S2 Appendix — (DOCX) [file pmed.1004429.s002.docx]

# **S2 Appendix: Parameters**

## **A. List of all parameters included in the model and their sources**

**S2 Table: Overview of model parameters and sources**

| **Parameter** | **Value (base case)** | **Interval** | **Distribution (parameter values)** | **Values used in sensitivity analysis** | **Sources** |
| --- | --- | --- | --- | --- | --- |
| **Behaviors of patients and healthcare staff** | | | | | |
| Test refusal among patients with severe disease and low oxygen presenting at care centers | 1% | range [0.1%,5%] | lognormal (-4.61,0.82) | None | Sources suggest test refusal rates are low overall. 0.5-1.2% in Zambia & Lesotho (for self- and professional testing) [1], 2% in Singapore [2] 2.5% in France [3]. |
| Share of severe tested patients that are not treated | 5% | NA | Fixed (5%) | 30% and 60% | Assumption. We assumed the share of non-treatment to be low at baseline (optimal context) but explore up to 60% in sensitivity analysis, based on reports of high treatment refusal in some countries during past pandemic peaks [4]. |
| Use of antibiotics after a positive SARS-CoV-2 test | 30% | Range [10%,50%] | Uniform (0.1,0.5) | None | Estimated range based on Bonnet et al., 2023 [4] expert consultation. |
| Added use of antibiotics with a negative test or no testing as compared to a positive test result | 30% | Range [10%,50%] | Uniform (0.1,0.5) | None | Estimated range based on Bonnet et al., 2023 [4] expert consultation. |
| **Screening and testing** | | | | | |
| Sensitivity of clinical COVID screening | 65.0% | range [35%,95%] | Uniform (35-95%) | None | Estimated range based on Bonnet et al., 2023 [4] experts consultation. |
| Specificity of clinical COVID screening | 75.0% | range [55%, 95%] | Uniform (55-95%) | None | Estimated range based on Bonnet et al., 2023 [4] expert consultation. |
| Sensitivity of SARS-CoV-2 RDT tests | 80.0% | NA | Fixed (80%) | 90%, 60% and 40% | Normative assumption at baseline: minimum 80% sensitivity recommended by WHO [5]. Some tests may have better performance (hence the 90%), while sensitivity may decline, particularly with newer variants. The 60% and 40% values are taken from ranges in the literature for the Omicron variant [6]. |
| Specificity of SARS-CoV-2 RDT tests | 98.7% | 95% confidence interval (CI) [93.3%,100%] | Beta (31.8,0.41) | None | In line with PCR [7].and RDT [8] specificity estimates in meta-analyses. |
| Sensitivity of SARS-CoV-2 PCR tests | 95.0% | range [83.5%,97.8%] | Uniform (83.5-97.8%) | None | 83.5-97.8% sensitivity for Omicron depending on the type of PCR test (89.0% for oropharyngeal swab, 83.5% for nasopharyngeal swab and 97.8% for saliva), based on a study of 246 adult patients [9]. |
| Specificity of SARS-CoV-2 PCR tests | 99.0% | 95% CI [93.3%,100%] | Beta (31.8,0.41) | None | In line with PCR [7].and RDT [8] specificity estimates in meta-analyses. |
| **Intended health impacts of treatment** | | | | | |
| Mortality risk, severe COVID patients not treated in hospital | 39% | 95% CI [1%,79%] | Beta (2.33,3.41) | None | Beta distribution fitted to answers from 28 expert respondents from Whittaker et al., 2022 [10] - excluding one outlier that would lead to figures above 200%. |
| Mortality risk, critical COVID patients not treated in hospital | 95% | NA | Fixed (95%) | None | Assumption as per Whittaker et al., 2022 [10]’s expert consultation. |
| Mortality risk, severe/critical COVID patients, treated with O_2_ and (if needed) MV | 13% | 95% CI [1%,49%] | Beta (1.15,5.94) | None | WHO therapeutic guidelines [11], uncertainty based on variability in ratios of reported deaths to hospital admissions for 2023 [12], LMICs with ratio of reported to estimated deaths ≥ 0.5. |
| Mortality risk, critical patients needing MV, when it is available | 39% | 95% CI [16%,65%] | Beta (5.27,8.19) | None | WHO therapeutic guidelines [11], uncertainty based on variability in ratios of reported deaths to hospital admissions for 2023 [12], LMICs with ratio of reported to estimated deaths ≥ 0.5. |
| Share of severe/critical COVID patients needing MV | 12% | 95% CI [7%,20%] | Lognormal  (-2.19,0.27) | None | WHO therapeutic guidelines [11] and systematic review [13]. Uncertainty based on Chang et al., 2021’s systematic review [14]. |
| Mortality risk, patients needing MV given conventional O_2_ instead | 95% | 95% CI [74%,100%] | Beta (12.5,1.0) | None | Assumption based on Whittaker et al., 2022 [10]’s expert consultation (95% or more without mechanical ventilation, RR of death without vs. with oxygen in moderate disease: 1.91). |
| OR of mortality, COVID patients treated vs. not treated with corticosteroids | 0.80 | 95% CI [0.65,0.94] | Normal (-0.80,0.074) | None | Siemieniuk et al., 2022 systematic review [13] |
| OR of needing MV, COVID patients treated vs, not treated with corticosteroids | 0.79 | 95% CI [0.58,1.05] | Lognormal (-0.253,0.150) | None | Siemieniuk et al., 2022 systematic review [13] |
| OR of mortality, COVID patients treated with corticosteroids and IL-6 receptor blockers vs. corticosteroids | 0.80 | 95% CI [0.69,0.94] | Lognormal (-0.226,0.079) | None | Siemieniuk et al., 2022 systematic review [13] |
| OR of needing MV, COVID patients treated with corticosteroids and IL-6 receptor blockers vs. corticosteroids | 0.79 | 95% CI [0.63,0.98] | Lognormal (-0.242,0.113) | None | Siemieniuk et al., 2022 systematic review [13] |
| Time in hospital, hospitalized severe/critical COVID cases, under SoC | 12.8 | IQR [9,17] | Weibull (14.9,2.45) | None | WHO therapeutic guidelines [11]. For distribution: LoS across studies within China: 14 [10-19] IQR [15]; LoS across countries: 13 [9-17.8] [16]. |
| Time in ICU, hospitalized COVID cases needing MV, when available, under SoC | 14.7 | IQR [10.3,19.5] | Weibull (17.1,2.45) | None | WHO therapeutic guidelines [11]. For distribution, as above. There are fewer studies than for hospital LoS. Range: 4-19 (19 being an outlier) across 8 studies in review [15]. |
| Time in hospital for hospitalized severe/critical COVID patients, when only conventional O_2_ is available | 12.8 | IQR [9,17] | Weibull (14.9,2.45) | None | Assumption. Data on that issue is lacking. Further, critical patients represent a small proportion of all severe and critical COVID patients so the impact on the average for both groups is likely relatively small. |
| Change in time spent in hospital, COVID patients treated vs. not treated with corticosteroids | 1.0 | 95% CI [-2.1,4.1] | Normal (1.0,1.6) | None | From Siemieniuk et al., 2022 systematic review [13] |
| Change in time spent in hospital, COVID patients treated with IL-6 receptor blockers + corticosteroids vs. corticosteroids alone | -4.7 | 95% CI [-7.7,-1.7] | Normal  (-4.7,1.5) | None | WHO therapeutic guidelines [11] and Siemieniuk et al., 2022 systematic review [13] |
| Change in the time spent in ICU, COVID patients treated vs. not treated with corticosteroids | -1.4 | 95% CI [-3.4,0.6] | Normal  (-1.4,1.0) | None | WHO therapeutic guidelines [11] |
| Change in the time spent in ICU, COVID patients treated with IL-6 receptor blockers + corticosteroids vs. corticosteroids alone | -1.1 | 95% CI [-2.3,0.1] | Normal (-1.1,0.6) | None | WHO therapeutic guidelines [11] and Siemieniuk et al., 2022 systematic review [13] |
| Change in the time spent in hospital, COVID patients treated vs. not treated with corticosteroids, when MV is not available | 1.0 | 95% CI [-2.1,4.1] | Normal (1.0,1.6) | None | Assumption. Data on that issue is lacking. Further, critical patients represent a small proportion of all severe and critical COVID patients so the impact on the average for both groups is likely relatively small. |
| DALYs incurred by 1 day of hospitalization in the general ward | 0.00036 | 95% CI [0.00025, 0.00054] | Lognormal (-7.937,0.197) | None | Estimates from Salomon et al., 2012 [17]’s Global Burden of Disease estimates, cited in Liu et al, 2022 [18] |
| DALYs incurred by 1 day of hospitalization in ICU | 0.00179 | 95% CI [0.00160, 0.00201] | Lognormal (-6.325,0.057) | None | Estimates from Haagsma et al., 2015 [19]’s Global Burden of Disease estimates, cited in Liu et al., 2022 [18] |
| DALYs incurred by 1 day at home with severe or critical COVID | 0.0011 | Range [0.0002,0.0020] | Uniform (0.0002,0.0020) | None | Assumption, broad range derived from DALYs incurred by 1 day in hospital: untreated patients at home may be worse off that they would have been in hospital, hence we assume a disability weight ranging from the minimum weight for hospitalization in the general ward to the maximum weight for hospitalization in ICU. |
| YLLs per COVID death | NA | Range [12.9,24.7] | Country-specific | None | We fitted a model estimating YLLs per COVID death as a function of the share of country population in different age ranges and GDP per capita using data from (Arolas et al., 2021) [20]. See Section G in S2 Appendix for details. |
| YLLs per death in a COVID-like severe/critical patient who does not have COVID | NA | Range [14.5,70.0] | Country-specific | None | We combined LRTI, URTI and TB in the Global Burden of Disease [21] to estimate YLLs for ‘typical’ COVID-like severe/critical patients without COVID and needing O_2_. |
| DALYs incurred from post-COVID syndrome per 1000 hospitalized COVID patients | 643 | 95% CI [597,689] | Normal | None | Analysis of post-acute sequelae of 20,580 hospitalized COVID patients, compared to non-hospitalized patients and non-COVID controls [22]. |
| Percentage difference in DALYs incurred from post-COVID syndrome in treated vs. untreated patients | 0% | NA | Fixed | -20%, 20% | For the rationale for these assumptions, see Section F in S2 Appendix. |
| Health system costs of long-COVID | 0 | NA | Fixed | None | See Section F in S2 appendix. |
| **Unintended health effects of treatment** | | | | | |
| Likelihood of suffering from mild corticosteroid side effects | 8.9% | range [0.7%,17%] | Uniform (0.7%,17%) | None | Range derived from Gallant et al., 1986 [23] and Richards, 2008 [24] |
| DALYs incurred by 10 days of mild corticosteroid side effects | 0.00131 | range [0.001,0.002] | Uniform (0.001,0.002) | None | DALY weight for corticosteroid side effects based on an array of mild conditions: mild insomnia, heart burn, mild anxiety disorder and mild diarrhea (0.023, 0.038, 0.045 and 0.073 DALYs respectively [19]) weighted based on their respective estimated probability. We use a range of 0.023-0.073 DALYs. |
| Likelihood of severe corticosteroid side effects | 0.450% | 95% CI [0.426%,0.476%] | Normal (0.450%, 0.013%) | None | Derived from the values for fractures, gastro-intestinal bleeding, venous thrombosis and heart failure in the line directly below |
| Added risk of fracture following short-term corticosteroid use | 0.118% | 95% CI [0.00104, 0.00135] | Lognormal  (-6.739,0.068) | None | Derived from Waljee et al., 2017 [25] population-based cohort study of private health insurance claims (based on 327,452 adults with short-term corticosteroid prescriptions out of a total of 1,548,945). |
| Added risk of gastro-intestinal bleeding following short-term corticosteroid use | 0.230% | 95% CI [0.00216, 0.00246] | Lognormal  (-6.075,0.033) | None | Derived from Yao et al., 2020 [26] self-controlled case-series of medical claim records (based on 2,623,327 adults who received a single steroid burst, out of a total population of 15,859,129 adults). |
| Added risk of venous thrombosis following short-term corticosteroid use | 0.058% | 95% CI [0.00049, 0.00068] | Lognormal  (-7.455,0.083) | None | Derived from Waljee et al., 2017 [25] population-based cohort study of private health insurance claims (based on 327,452 adults with short-term corticosteroid prescriptions out of a total of 1,548,945). |
| Added risk of sepsis following short-term corticosteroid use | 0.025% | 95% CI [0.00018, 0.00030] | Lognormal  (-8.377,0.136) | None | Derived from Waljee et al, 2017 [25] (based on 327,452 adults with short-term corticosteroid prescriptions out of a total of 1,548,945) and Yao et al., 2020 [26] (based on 2,623,327 adults who received a single steroid burst, out of a total population of 15,859,129 adults. |
| Added risk of heart failure following short-term corticosteroid use | 0.020% | 95% CI [0.00017, 0.00024] | Lognormal  (-8.510,0.095) | None | Derived from Yao et al., 2020[26] self-controlled case-series of medical claim records (based on 2,623,327 adults who received a single steroid burst, out of a total population of 15,859,129 adults). |
| Mortality rate from severe corticosteroid side effects | 12% | Approx.. 95% CI [0.1,0.2] | Sum of distributions | None | See details in the lines immediately below. Severe side effects include bone fractures, gastro-intestinal bleeding, sepsis, venous thrombosis and heart failure [25,26]. |
| CFR, bone fractures | 0.36% | 95% CI [0.31%,0.42%] | Lognormal  (-5.630,0.079) | None | Derived, for the purpose of giving broad estimates of corticosteroid-induced bone fractures (resulting mostly from osteoporosis and/or muscle weakness [27]), from the closest we could find i.e. estimates from falls: 556,000 [449,000-611,000] deaths for 20.5 million (20.1–20.9) inpatient and 134 million (131–137) outpatient fall-related injuries [28]. |
| CFR, gastro-intestinal bleeding | 9.500% | range [4%,15%] | Uniform  (4%,15%) | None | Mortality from upper gastro-intestinal bleeding: 5-19% [29,30], from lower gastro-intestinal bleeding: 2-4% with 20-30% of cases being lower gastro-intestinal bleeds [31] |
| CFR venous thrombosis | 20.000% | range [10%,30%] | Uniform  (10%,30%) | None | As per Beckman et al., 2010 [32] |
| CFR, sepsis (immediate risk) | NA | NA | Country-specific | None | As per Rudd et al., 2020 [33] |
| Risk of death within a year of sepsis | 11.8% | 95% CI  [7.4%,16.1%] | Normal  (0.118,0.0222) | None | Prescott et al., 2018 [34] estimate that around 27% of sepsis patients die but that 16.2% (10.2% to 22.2%) [35] of those that recover die within the following year as a consequence of their sepsis (based on 504 matched pairs of patients), which corresponds to around 12.1% of all sepsis cases. |
| CFR, heart failure | 71.000% | [42%,100%] | Uniform (42%,100%) | None | 42% within a cohort of UK patients with a diagnosis of heart failure [36] (based on data from a cohort of 55,959 heart failure patients and 278,679 matched controls). We assume figures in LMICs are higher hence propose a range between 42 and 100%. |
| YLDs associated with severe corticosteroid side effects | NA | NA | Sum of distributions | None | See in the lines immediately below. |
| Ratio of YLL to DALYs incurred through fractures | 0.45760 | range [17%,75%] | Uniform (17-75%) | None | Derived from estimates for fall injuries [28]. Ratio of YLL to DALY lost through fractures around 0.45760 (range: 17-75%). |
| YLD per case of GI bleeding (reflecting the disability weight during the recovery period) | 0.0398 | 95% CI  [0.0264,0.0568] | Lognormal  (-3.243,0.196) | None | The GBD weight [17] for gastric bleeding has been estimated at 0.323 (0.214-0.461). Most patients appear to recover rapidly (within 45 days after discharge) [37]. |
| Disability weight for survivors of venous thromboembolism (lifelong) | 0.0231 | range  [0.017,0.029] | Uniform [0.017,0.029] | None | Over the life course of survivors of venous thromboembolism (VT), the disability weight of VT has been assessed at 0.0231 in Australia. This is not far from the estimated disability weight for survivors of pulmonary embolism after one year, i.e., 0.017 [38]. Limited information is available to build a confidence interval on this value, and we therefore chose a 0.017-0.029 plausible range. |
| Disability weight for recovered sepsis cases (lifelong) | 0.110 | Range  [0.055,0.165] | Uniform  (0.055,0.165) | None | Prescott et al., 2018 [34] estimate that 1/6 of sepsis cases (around 28% of those that do not die as a direct or indirect consequence of sepsis within a year) will have a severe long-term physical or cognitive disability. Mental health issues and exacerbation of pre-existing comorbidities also appear common. The GBD weights [39] for severe motor impairment, moderate dementia and severe motor plus cognitive impairment are 0.377 (0.251-0.518), 0.346 (0.233–0.475), and 0.425 (0.286–0.587) respectively. We use 0.4 [0.2-0.6] as a broad estimate of the weight of post-sepsis disability. |
| Average disability weight for heart failure patients | 0.0897 | 95% CI  [0.0538,0.1494] | Lognormal  (-2.445,0.260) | None | A secondary analysis of GBD data [40] has estimated the burden of heart failure globally at 5.04 [3.12-7.59] million YLDs in 2019 for 56.2 [43.4-71.67] million prevalent cases, or around 0.0897 YLDs per heart failure case per year. |
| Life expectancy with heart failure | 5.54 | 95% CI  [1.63,13.94] | Lognormal (1.562,0.547) | None | The life expectancy for patients with heart failure was estimated in one study at 5.54 (SD 3.80) [41]. We assume this life expectancy follows a lognormal distribution. |
| Increase in mortality risk for non-COVID cases associated with disease-specific impacts of corticosteroids. | 0% | NA | Fixed (0%) | 5% | Baseline assumes no effect on average. Effects are country- and season-specific (depending on the frequency/nature of other COVID-like diseases). The rationale for the order of magnitude used in sensitivity analysis is discussed in Section E in S2 Appendix. |
| Rate of mild gastro-intestinal side effects (diarrhea or nausea) with IL-6 receptor blockers [per patient-year] | 0.364 | 95% CI [0.352,0.376] | Normal (0.364, 0.006) | None | As per Schiff et al., 2011 [42] |
| Added rate of URTI with IL-6 receptor blockers [(per patient year] | 0.0691 | 95% CI [-0.0239, 0.1611] | Normal (0.069,0.047) | None | Derived from Schiff et al., 2011 [42]. Infections typically are URTIs or nasopharyngitis. |
| Added rate of TB (new/reactivated) with IL-6 receptor blockers [per patient year] | 0.00083 | 95% CI [0.00026, 0.00140] | Normal (0.00083, 0.00029) | None | As per Cantini et al., 2017 [43] and Schiff et al., 2011 [42]: impact suspected on TB development or reactivation. |
| DALYs resulting from mild gastro-intestinal side effects associated with IL-6 receptor blockers | 0.00020 | 95% CI [0.00014, 0.00030] | Lognormal (-8.523,0.192) | None | As per the Global Burden of Disease 2019 [21] (note: we assume 10 days of mild side effects) |
| DALYs resulting from URTIs associated with IL-6 receptor blockers | 0.00014 | 95% CI [0.00009, 0.00021] | Lognormal (-8.899,0.214) | None | As per the Global Burden of Disease 2019 [21] (note: we assume URTIs driven by IL-6 receptor blocker use last for 7-10 days) |
| Average time (in years) with TB disease per TB patient | 1.6 | 95% CI [0.78,2.42] | Normal (1.60,0.42) | None | Estimate averaging over treated and untreated: - Time to resolution (death or cure), untreated patients: 2.2-3.3, estimate 3 years[44].  - Treatment duration (treated patients): international guidelines: often 4, 6 or 9 months [45,46], some variability depending on the country/patient [47,48]. - Time to treatment initiation (treated patients): derived from multiple studies with close to 2 months the most common duration 65 days [49], 66 days [50], 67 days [51], patient delay: 24.6 days (17-63 days depending on the study) [52].  - Case detection rate: country-specific, median: 60% [53]. |
| Average duration of TB treatment, per TB patient | 0.3 | 95% CI [0.07, 0.53] | Normal (0.30,0.12) | None | Estimate averaging over treated and untreated as per data above. |
| TB case fatality rate | NA | NA | Country-specific | None | As per data in the Global Tuberculosis Report 2022 [53]. |
| DALY weight per year of symptomatic TB | 0.333 | 95% CI [0.218,0.448] | Normal (0.333,0.059) | None | As per Abate et al., 2017 [54]. |
| **Model cost estimates** | | | | | |
| Screening costs | NA | Range [$0.7, $8.3] | Country-specific | None | See Section C.1 in S2 appendix. |
| Estimated cost of a PCR test kit | $ 27.2 | 95% CI [$7.10, $140.25] | Lognormal  (2.99,0.79) | None | See Section C.2 in S2 appendix. |
| Estimated cost of an RDT test kit | $ 2 | Range  [$0.6, $6.2] | Fixed | $0.6, $6.2 | See Section C.2 in S2 appendix. |
| Estimated cost of a PCR test | NA | Average: $5.6  Range [$2.32, $12.31] | Country-specific | None | See Section C.2 in S2 appendix. |
| Estimated cost of an RDT test | NA | Average: $4.9  Range [$1.6, $13.3] | Country-specific | None | See Section C.2 in S2 appendix. |
| Estimated cost of a general bed-day | NA | Range [$17, $565] | Country-specific | None | Based on Torres-Rueda, 2021 [55] (updated to 2021). |
| Estimated cost of an ICU bed-day | NA | Range [$102, $5,330] | Country-specific | None | Based on Torres-Rueda, 2021 [55] (updated to 2021). |
| Cost of a corticosteroid treatment course | $ 0.19 | 95% CI [$0.2, $2.3] | $ 0.19 + Lognormal  (-0.97,1.30) | None | Could be developed for just $0.19 [56], list price assumed to be development price + a benefit following a lognormal distribution. |
| Cost of IL-6 receptor blocker treatment course | $ 861.5 | NA | Fixed ($861.5) | $ 411 and $1,207 | As per median, minimum and maximum in Wang et al., 2019 [56]. We also assessed the maximum plausible costs for which TCZ use may be cost-effective. |
| Cost of an antimicrobial treatment course | $ 0.70 | 95% CI [$0.1, $5.0] | Lognormal (-0.35,1.00) | No | Range in literature from very low to $5 (for the US), with most costs available in LMICs below $2 per treatment course:   - 500-1500 mg a day for 7-10 days of treatment with amoxicillin: $0.21-1.62 [57]. - Azithromycin treatment course: $0-0.8 in Thailand, $0.32-1.09 in India, $0.2-5 in the US [58,59].   We used a median of $0.7 and a range of $0.1-5. |
| Cost of treating a patient for the mild side effects of IL-6 receptor blockers, per patient with mild side effects | $ 0.20 | 95% CI [$0.03, $1.36] | lognormal  (-1.61,0.98) | No | See Section D.6 in S2 Appendix. |
| Cost of treating a patient for the mild side effects of corticosteroids, per patient with mild side effects | $ 0.06 | 95% CI [$0.01, $0.60] | Lognormal  (-2.81,2.30) | No | Potential mild corticosteroid side effects include: insomnia, heart burn, mild anxiety disorder and mild diarrhea [24]. Many of those may remain untreated. Those that are treated may receive e.g. anti-nausea medication [57]. We assume that costs are similar to those of treating around half of patients with side effects with anti-nausea medication. |
| Cost of treatment for patients for severe corticosteroid side effects | NA | NA | See below | No | See below and in Section D in S2 Appendix for the costs of treatment of bone fractures, gastro-intestinal bleeding, venous thrombosis, sepsis and heart failure. |
| Treatment costs per patient, bone fractures - Low-income countries  - Lower-middle income countries  - Upper middle income countries | $1,752 $1,843 $2,844 | Ranges: [$428, $7,180] [$450, $5,554] [$694, $11,657] | Fixed | Yes (low/high of ranges) | See Section D.1 in S2 Appendix and S5 Table for the source of the medians and intervals at each income level. |
| Treatment costs per patient, gastro-intestinal bleeding | NA | Country-specific, range across countries: $26 to $1,466 | Fixed | Yes (low/high estimates per country) | See Section D.2 in S2 Appendix for details. |
| Treatment costs per patient, venous thrombosis | NA | Country-specific, range across countries: $468 to $16,084 | Fixed | Yes (low/high estimates per country) | See Section D.3 in S2 Appendix for details. |
| Treatment costs per patient, sepsis | NA | Country-specific, range across countries: $24 to $4,833 | Fixed | Yes (low/high estimates per country) | See Section D.4 in S2 Appendix for details. |
| Treatment costs per patient, heart failure | $4,023 | Range [$473, $7572] | Fixed | Yes (low/high end of range) | See Section D.5 in S2 Appendix for details. |
| Cost of TB treatment (per month of treatment) | NA | Country-specific, range across countries: $31 to $196 | Lognormal | No | As per Siapka et al., 2020 [60] using most recent GDP/capita, TB detection and HIV prevalence rates. |
| Cost of a body bag (per death) | $ 68.3 | NA | Fixed ($ 68.3) | No | As per Torres-Rueda et al., 2021 [55] |
| **Other parameters** | | | | | |
| Cost-effectiveness threshold | NA | NA | Country-specific | No | Ochalek et al. thresholds [61], based on the health opportunity cost of healthcare spending using econometric analyses of global data. Using updated values of GDP/capita for countries with data and interpolation for countries without data. The threshold was given a uniform distribution with an expected value and range corresponding, respectively, to the median, highest, and lowest empirical estimates. |
| Cost discounting rate | 3% | NA | Fixed | No | 3% discounting rate. |
| Health impact discounting rate | 0% | NA | Fixed | Yes (3%) | 0% at baseline, 3% in sensitivity analysis [62]. |

## **B. Country-specific parameter values**

**S3 Table Country-specific parameters**

| **Country name** | **Income level** | **GDP per capita (2021)** | **Cost PCR testing** | **Cost RDT testing** | **Screen costs*** | **Cost per bed day (general ward)** | **Cost per bed day (ICU)** | **Sepsis CFR** | **TB CFR** | **YLL per COVID death** | **YLL per COVID-like death** | **YLL per heart failure death** | **Threshold (median value)** | **Cost TB treatment (per month)** | **Cost of treating sepsis** | **Cost of treating GI bleeding** | **Cost of treating VT** |
| --- | --- | --- | --- | --- | --- | --- | --- | --- | --- | --- | --- | --- | --- | --- | --- | --- | --- |
| Jordan | UMIC | $ 4,103 | $ 33.4 | $ 10.2 | $ 4.1 | 85 | 940 | 12% | 2% | 20.8 | 43.5 | 23.2 | $ 6,403 | $ 111 | $ 874 | $ 470 | $ 3,199 |
| Tonga | UMIC | $ 4,426 | $ 33.6 | $ 10.4 | $ 4.3 | 67 | 762 | 32% | 10% | 19.0 | 27.9 | 21.2 | $ 935 | $ 115 | $ 695 | $ 514 | $ 2,513 |
| Fiji | UMIC | $ 4,647 | $ 33.8 | $ 10.6 | $ 4.4 | 95 | 1,022 | 34% | 8% | 19.5 | 41.5 | 26.3 | $ 984 | $ 116 | $ 487 | $ 540 | $ 3,603 |
| Iraq | UMIC | $ 4,775 | $ 33.9 | $ 10.7 | $ 4.5 | 122 | 1,268 | 18% | 9% | 19.8 | 46.1 | 22.4 | $ 2,868 | $ 119 | $ 539 | $ 554 | $ 4,606 |
| Namibia | UMIC | $ 4,866 | $ 33.9 | $ 10.7 | $ 4.6 | 100 | 1,071 | 28% | 24% | 18.8 | 41.8 | 20.8 | $ 3,853 | $ 143 | $ 1,177 | $ 571 | $ 3,769 |
| Suriname | UMIC | $ 4,869 | $ 33.9 | $ 10.7 | $ 4.6 | 109 | 1,135 | 25% | 20% | 19.1 | 27.2 | 21.5 | $ 2,111 | $ 121 | $ 1,492 | $ 566 | $ 4,102 |
| Armenia | UMIC | $ 4,967 | $ 34.0 | $ 10.8 | $ 4.6 | 91 | 1,004 | 24% | 9% | 16.9 | 32.3 | 16.8 | $ 1,641 | $ 122 | $ 1,871 | $ 577 | $ 3,439 |
| Georgia | UMIC | $ 5,023 | $ 34.0 | $ 10.8 | $ 4.7 | 100 | 1,084 | 34% | 6% | 15.3 | 26.5 | 16.4 | $ 1,166 | $ 120 | $ 952 | $ 584 | $ 3,783 |
| Guatemala | UMIC | $ 5,026 | $ 34.0 | $ 10.9 | $ 4.7 | 84 | 938 | 21% | 16% | 21.1 | 39.5 | 19.3 | $ 1,875 | $ 122 | $ 840 | $ 584 | $ 3,173 |
| Jamaica | UMIC | $ 5,184 | $ 34.1 | $ 11.0 | $ 4.8 | 85 | 929 | 28% | 15% | 21.4 | 20.0 | 16.7 | $ 2,673 | $ 125 | $ 974 | $ 602 | $ 3,202 |
| Moldova | UMIC | $ 5,231 | $ 34.1 | $ 11.0 | $ 4.8 | 49 | 344 | 27% | 13% | 16.9 | 35.0 | 16.2 | $ 3,881 | $ 133 | $ 903 | $ 608 | $ 1,851 |
| Azerbaijan | UMIC | $ 5,388 | $ 34.2 | $ 11.1 | $ 4.9 | 140 | 1,465 | 17% | 14% | 19.3 | 58.6 | 20.5 | $ 1,607 | $ 126 | $ 503 | $ 628 | $ 5,283 |
| Paraguay | UMIC | $ 5,891 | $ 34.5 | $ 11.4 | $ 5.2 | 108 | 1,145 | 26% | 12% | 19.1 | 28.3 | 19.9 | $ 4,555 | $ 135 | $ 1,278 | $ 692 | $ 4,083 |
| Ecuador | UMIC | $ 5,965 | $ 34.5 | $ 11.5 | $ 5.2 | 102 | 1,088 | 22% | 14% | 20.3 | 29.1 | 19.0 | $ 5,511 | $ 116 | $ 1,569 | $ 693 | $ 3,860 |
| Colombia | UMIC | $ 6,104 | $ 34.6 | $ 11.6 | $ 5.3 | 110 | 1,145 | 18% | 12% | 19.7 | 28.6 | 16.5 | $ 8,932 | $ 131 | $ 1,563 | $ 718 | $ 4,146 |
| Belize | UMIC | $ 6,228 | $ 34.7 | $ 11.6 | $ 5.3 | 85 | 937 | 24% | 14% | 21.1 | 31.3 | 21.0 | $ 3,919 | $ 140 | $ 800 | $ 724 | $ 3,191 |
| Libya | UMIC | $ 6,357 | $ 34.8 | $ 11.7 | $ 5.4 | 89 | 907 | 16% | 25% | 20.7 | 27.3 | 22.8 | $ 4,212 | $ 134 | $ 1,035 | $ 686 | $ 3,368 |
| Albania | UMIC | $ 6,493 | $ 34.8 | $ 11.8 | $ 5.5 | 118 | 1,262 | 29% | 3% | 17.1 | 27.0 | 16.3 | $ 4,538 | $ 141 | $ 1,068 | $ 741 | $ 4,470 |
| Peru | UMIC | $ 6,622 | $ 34.9 | $ 11.9 | $ 5.6 | 111 | 1,153 | 17% | 11% | 19.5 | 23.0 | 16.4 | $ 5,241 | $ 136 | $ 1,212 | $ 771 | $ 4,172 |
| North Macedonia | UMIC | $ 6,695 | $ 34.9 | $ 11.9 | $ 5.6 | 136 | 1,413 | 31% | 11% | 17.4 | 24.2 | 18.3 | $ 6,330 | $ 137 | $ 1,465 | $ 778 | $ 5,120 |
| Botswana | UMIC | $ 6,805 | $ 35.0 | $ 12.0 | $ 5.6 | 142 | 1,446 | 24% | 35% | 19.8 | 44.1 | 24.4 | $ 3,050 | $ 180 | $ 1,113 | $ 841 | $ 5,371 |
| South Africa | UMIC | $ 7,055 | $ 35.1 | $ 12.1 | $ 5.8 | 116 | 1,188 | 31% | 19% | 18.1 | 41.9 | 20.6 | $ 3,405 | $ 174 | $ 1,614 | $ 820 | $ 4,388 |
| Thailand | UMIC | $ 7,066 | $ 35.1 | $ 12.1 | $ 5.8 | 133 | 1,361 | 24% | 11% | 17.1 | 20.5 | 18.6 | $ 6,588 | $ 137 | $ 898 | $ 820 | $ 5,027 |
| Bosnia & Herzegovina | UMIC | $ 7,143 | $ 35.2 | $ 12.2 | $ 5.8 | 125 | 1,313 | 38% | 12% | 15.7 | 18.4 | 16.1 | $ 1,939 | $ 142 | $ 2,040 | $ 840 | $ 4,720 |
| Belarus | UMIC | $ 7,302 | $ 35.2 | $ 12.3 | $ 5.9 | 144 | 1,477 | 24% | 17% | 15.5 | 28.2 | 16.4 | $ 4,872 | $ 143 | $ 1,288 | $ 870 | $ 5,432 |
| Turkmenistan | UMIC | $ 7,345 | $ 35.3 | $ 12.3 | $ 5.9 | 137 | 1,399 | 20% | 27% | 18.4 | 66.6 | 21.1 | $ 2,336 | $ 144 | $ 1,590 | $ 848 | $ 5,161 |
| Equatorial Guinea | UMIC | $ 7,507 | $ 35.4 | $ 12.4 | $ 6.0 | 184 | 1,827 | 15% | 37% | 17.3 | 42.4 | 21.7 | $ 7,906 | $ 160 | $ 655 | $ 872 | $ 6,946 |
| Brazil | UMIC | $ 7,507 | $ 35.4 | $ 12.4 | $ 6.0 | 109 | 1,108 | 25% | 8% | 19.2 | 23.4 | 20.9 | $ 6,625 | $ 148 | $ 2,517 | $ 894 | $ 4,104 |
| Dominican Rep. | UMIC | $ 8,477 | $ 35.8 | $ 13.0 | $ 6.5 | 134 | 1,368 | 15% | 13% | 19.5 | 34.8 | 21.0 | $ 4,344 | $ 152 | $ 1,080 | $ 985 | $ 5,062 |
| Gabon | UMIC | $ 8,635 | $ 35.9 | $ 13.0 | $ 6.6 | 160 | 1,631 | 25% | 34% | 18.1 | 35.8 | 22.0 | $ 3,316 | $ 133 | $ 629 | $ 1,003 | $ 6,032 |
| St. Vincent & the Grenadines | UMIC | $ 8,666 | $ 35.9 | $ 13.1 | $ 6.6 | 109 | 1,146 | 33% | 11% | 18.3 | 22.1 | 17.9 | $ 2,268 | $ 154 | $ 1,262 | $ 971 | $ 4,120 |
| Grenada | UMIC | $ 9,011 | $ 36.1 | $ 13.3 | $ 6.7 | 133 | 1,338 | 39% | 93% | 17.1 | 22.6 | 20.3 | $ 2,698 | $ 157 | $ 1,620 | $ 1,047 | $ 5,011 |
| Mauritius | UMIC | $ 9,106 | $ 36.1 | $ 13.3 | $ 6.8 | 152 | 1,502 | 27% | 17% | 19.1 | 21.4 | 20.0 | $ 4,309 | $ 160 | $ 1,908 | $ 1,053 | $ 5,752 |
| Serbia | UMIC | $ 9,230 | $ 36.2 | $ 13.4 | $ 6.8 | 148 | 1,522 | 34% | 12% | 14.1 | 18.6 | 15.5 | $ 3,517 | $ 164 | $ 2,391 | $ 1,072 | $ 5,589 |
| Saint Lucia | UMIC | $ 9,414 | $ 36.3 | $ 13.5 | $ 6.9 | 116 | 1,172 | 31% | 93% | 19.5 | 20.4 | 17.9 | $ 1,957 | $ 160 | $ 2,110 | $ 1,094 | $ 4,367 |
| Montenegro | UMIC | $ 9,466 | $ 36.3 | $ 13.5 | $ 7.0 | 165 | 1,657 | 29% | 2% | 16.3 | 20.6 | 17.6 | $ 3,898 | $ 164 | $ 3,273 | $ 1,100 | $ 6,216 |
| Cuba | UMIC | $ 9,500 | $ 36.3 | $ 13.5 | $ 7.0 | 308 | 3,554 | 27% | 10% | 14.1 | 14.5 | 16.1 | $ 5,314 | $ 165 | $ 4,833 | $ 1,104 | $ 11,638 |
| Turkey | UMIC | $ 9,661 | $ 36.4 | $ 13.6 | $ 7.0 | 174 | 1,725 | 19% | 7% | 18.6 | 20.8 | 18.1 | $ 8,467 | $ 162 | $ 1,238 | $ 1,122 | $ 6,587 |
| Guyana | UMIC | $ 9,999 | $ 36.5 | $ 13.8 | $ 7.2 | 74 | 820 | 27% | 27% | 19.9 | 33.4 | 23.3 | $ 3,429 | $ 168 | $ 1,171 | $ 1,161 | $ 2,795 |
| Mexico | UMIC | $ 10,046 | $ 36.6 | $ 13.8 | $ 7.2 | 153 | 1,531 | 22% | 15% | 19.8 | 30.9 | 18.4 | $ 7,445 | $ 162 | $ 1,816 | $ 1,167 | $ 5,763 |
| Maldives | UMIC | $ 10,366 | $ 36.7 | $ 14.0 | $ 7.4 | 130 | 1,318 | 11% | 6% | 19.8 | 27.0 | 20.7 | $ 2,398 | $ 167 | $ 3,084 | $ 1,204 | $ 4,921 |
| Kazakhstan | UMIC | $ 10,374 | $ 36.7 | $ 14.0 | $ 7.4 | 196 | 1,938 | 23% | 9% | 16.3 | 38.5 | 18.8 | $ 4,727 | $ 183 | $ 1,033 | $ 1,205 | $ 7,416 |
| Argentina | UMIC | $ 10,636 | $ 36.8 | $ 14.1 | $ 7.5 | 170 | 1,685 | 27% | 8% | 18.2 | 17.4 | 16.9 | $ 4,854 | $ 169 | $ 3,262 | $ 1,236 | $ 6,434 |
| Malaysia | UMIC | $ 11,109 | $ 37.0 | $ 14.4 | $ 7.7 | 216 | 2,110 | 21% | 8% | 19.8 | 22.2 | 22.4 | $ 6,745 | $ 175 | $ 1,329 | $ 1,291 | $ 8,173 |
| Russian Federation | UMIC | $ 12,195 | $ 37.5 | $ 14.9 | $ 8.2 | 190 | 1,867 | 25% | 13% | 15.5 | 32.9 | 17.3 | $ 7,777 | $ 196 | $ 2,847 | $ 1,463 | $ 7,189 |
| Bulgaria | UMIC | $ 12,221 | $ 37.5 | $ 14.9 | $ 8.2 | 183 | 1,834 | 32% | 8% | 12.9 | 22.8 | 15.9 | $ 8,928 | $ 179 | $ 3,229 | $ 1,420 | $ 6,918 |
| Costa Rica | UMIC | $ 12,472 | $ 37.6 | $ 15.0 | $ 8.3 | 138 | 1,373 | 23% | 13% | 18.4 | 21.8 | 17.7 | $ 17,913 | $ 176 | $ 3,685 | $ 1,456 | $ 5,223 |
| China | UMIC | $ 12,556 | $ 37.7 | $ 15.1 | $ 8.3 | 157 | 1,578 | 24% | 4% | 16.0 | 22.3 | 17.5 | $ 7,357 | $ 182 | $ 2,005 | $ 1,466 | $ 5,917 |
| Tajikistan | LMIC | $ 897 | $ 32.3 | $ 11.2 | $ 1.6 | 37 | 326 | 14% | 15% | 18.6 | 64.8 | 22.0 | $ 356 | $ 60 | $ 144 | $ 106 | $ 1,062 |
| Lesotho | LMIC | $ 1,094 | $ 32.7 | $ 11.6 | $ 1.8 | 36 | 262 | 40% | 40% | 19.9 | 47.1 | 24.6 | $ 633 | $ 76 | $ 245 | $ 121 | $ 1,035 |
| Tanzania | LMIC | $ 1,099 | $ 32.7 | $ 11.6 | $ 1.8 | 43 | 369 | 25% | 22% | 17.2 | 52.0 | 21.3 | $ 311 | $ 57 | $ 71 | $ 133 | $ 1,234 |
| Nepal | LMIC | $ 1,208 | $ 32.9 | $ 11.9 | $ 1.9 | 43 | 359 | 28% | 27% | 21.7 | 40.2 | 23.0 | $ 345 | $ 65 | $ 115 | $ 143 | $ 1,213 |
| Myanmar | LMIC | $ 1,210 | $ 32.9 | $ 11.9 | $ 1.9 | 38 | 273 | 30% | 19% | 20.4 | 39.8 | 22.0 | $ 677 | $ 64 | $ 150 | $ 141 | $ 1,084 |
| Kyrgyz Republic | LMIC | $ 1,277 | $ 33.0 | $ 12.0 | $ 2.0 | 34 | 244 | 18% | 8% | 18.1 | 56.8 | 18.1 | $ 931 | $ 68 | $ 129 | $ 152 | $ 966 |
| Benin | LMIC | $ 1,319 | $ 33.1 | $ 12.1 | $ 2.0 | 41 | 328 | 21% | 25% | 16.8 | 58.0 | 22.0 | $ 250 | $ 63 | $ 55 | $ 158 | $ 1,162 |
| Pakistan | LMIC | $ 1,505 | $ 33.4 | $ 12.5 | $ 2.2 | 37 | 247 | 21% | 8% | 19.8 | 57.9 | 27.7 | $ 177 | $ 69 | $ 68 | $ 175 | $ 1,058 |
| Comoros | LMIC | $ 1,577 | $ 33.5 | $ 12.7 | $ 2.2 | 36 | 258 | 34% | 26% | 18.3 | 38.4 | 20.7 | $ 320 | $ 67 | $ 173 | $ 183 | $ 1,020 |
| Kiribati | LMIC | $ 1,606 | $ 33.5 | $ 12.7 | $ 2.3 | 40 | 285 | 47% | 9% | 18.2 | 41.1 | 31.6 | $ 210 | $ 80 | $ 424 | $ 205 | $ 1,132 |
| Cambodia | LMIC | $ 1,625 | $ 33.5 | $ 12.8 | $ 2.3 | 35 | 249 | 34% | 9% | 18.9 | 38.5 | 22.4 | $ 331 | $ 72 | $ 270 | $ 189 | $ 989 |
| Senegal | LMIC | $ 1,637 | $ 33.6 | $ 12.8 | $ 2.3 | 38 | 273 | 27% | 16% | 18.4 | 43.9 | 20.7 | $ 444 | $ 72 | $ 162 | $ 190 | $ 1,084 |
| Cameroon | LMIC | $ 1,667 | $ 33.6 | $ 12.9 | $ 2.3 | 38 | 274 | 22% | 28% | 17.6 | 51.8 | 23.0 | $ 142 | $ 69 | $ 115 | $ 194 | $ 1,087 |
| Zimbabwe | LMIC | $ 1,774 | $ 33.8 | $ 13.1 | $ 2.4 | 565 | 4,023 | 35% | 25% | 19.7 | 48.1 | 23.0 | $ 303 | $ 91 | $ 97 | $ 206 | $ 16,084 |
| Haiti | LMIC | $ 1,830 | $ 33.9 | $ 13.2 | $ 2.5 | 52 | 396 | 27% | 11% | 20.5 | 56.3 | 23.8 | $ 213 | $ 83 | $ 82 | $ 212 | $ 1,480 |
| Angola | LMIC | $ 1,954 | $ 34.0 | $ 13.4 | $ 2.6 | 42 | 294 | 22% | 19% | 16.2 | 51.6 | 25.0 | $ 833 | $ 70 | $ 97 | $ 221 | $ 1,193 |
| Uzbekistan | LMIC | $ 1,983 | $ 34.1 | $ 13.5 | $ 2.6 | 39 | 278 | 18% | 7% | 18.3 | 65.8 | 23.5 | $ 874 | $ 80 | $ 284 | $ 232 | $ 1,111 |
| Nicaragua | LMIC | $ 2,046 | $ 34.2 | $ 13.6 | $ 2.6 | 39 | 278 | 18% | 9% | 20.9 | 43.1 | 18.0 | $ 2,546 | $ 84 | $ 407 | $ 240 | $ 1,114 |
| Nigeria | LMIC | $ 2,066 | $ 34.2 | $ 13.6 | $ 2.7 | 46 | 324 | 14% | 28% | 17.3 | 65.4 | 21.2 | $ 187 | $ 63 | $ 144 | $ 240 | $ 1,297 |
| Kenya | LMIC | $ 2,082 | $ 34.2 | $ 13.6 | $ 2.7 | 46 | 428 | 20% | 25% | 20.4 | 44.0 | 23.2 | $ 791 | $ 78 | $ 180 | $ 240 | $ 1,318 |
| Mauritania | LMIC | $ 2,166 | $ 34.3 | $ 13.8 | $ 2.7 | 40 | 291 | 16% | 18% | 18.6 | 38.4 | 19.4 | $ 432 | $ 77 | $ 117 | $ 252 | $ 1,150 |
| India | LMIC | $ 2,257 | $ 34.5 | $ 14.0 | $ 2.8 | 49 | 418 | 26% | 17% | 21.1 | 38.0 | 24.1 | $ 442 | $ 81 | $ 111 | $ 260 | $ 1,406 |
| Congo, Rep. | LMIC | $ 2,290 | $ 34.5 | $ 14.0 | $ 2.8 | 44 | 315 | 27% | 28% | 18.5 | 40.5 | 24.2 | $ 1,387 | $ 80 | $ 173 | $ 266 | $ 1,263 |
| Solomon Islands | LMIC | $ 2,305 | $ 34.5 | $ 14.0 | $ 2.9 | 39 | 277 | 42% | 11% | 18.2 | 45.7 | 32.0 | $ 258 | $ 94 | $ 223 | $ 259 | $ 1,108 |
| Sao Tome & Principe | LMIC | $ 2,361 | $ 34.6 | $ 14.1 | $ 2.9 | 43 | 310 | 32% | 34% | 19.2 | 34.3 | 20.5 | $ 356 | $ 95 | $ 245 | $ 274 | $ 1,236 |
| Ghana | LMIC | $ 2,363 | $ 34.6 | $ 14.1 | $ 2.9 | 42 | 300 | 22% | 39% | 19.3 | 40.7 | 22.1 | $ 592 | $ 70 | $ 184 | $ 280 | $ 1,203 |
| Bangladesh | LMIC | $ 2,458 | $ 34.7 | $ 14.3 | $ 3.0 | 38 | 275 | 19% | 12% | 21.8 | 39.1 | 22.9 | $ 274 | $ 86 | $ 97 | $ 286 | $ 1,095 |
| Lao PDR | LMIC | $ 2,536 | $ 34.8 | $ 14.4 | $ 3.0 | 41 | 287 | 27% | 22% | 20.7 | 46.9 | 23.7 | $ 370 | $ 78 | $ 140 | $ 295 | $ 1,158 |
| Cote d'Ivoire | LMIC | $ 2,549 | $ 34.8 | $ 14.5 | $ 3.0 | 39 | 281 | 26% | 23% | 19.0 | 54.8 | 23.9 | $ 300 | $ 85 | $ 185 | $ 304 | $ 1,119 |
| Papua New Guinea | LMIC | $ 2,673 | $ 35.0 | $ 14.7 | $ 3.1 | 46 | 323 | 29% | 13% | 20.1 | 68.2 | 29.1 | $ 303 | $ 92 | $ 129 | $ 307 | $ 1,299 |
| Timor-Leste | LMIC | $ 2,741 | $ 35.1 | $ 14.8 | $ 3.2 | 50 | 357 | 20% | 20% | 20.7 | 42.3 | 21.7 | $ 289 | $ 85 | $ 285 | $ 318 | $ 1,433 |
| Honduras | LMIC | $ 2,772 | $ 35.1 | $ 14.8 | $ 3.2 | 45 | 317 | 27% | 22% | 22.0 | 34.8 | 19.5 | $ 2,633 | $ 95 | $ 574 | $ 322 | $ 1,275 |
| Vanuatu | LMIC | $ 2,997 | $ 35.4 | $ 15.2 | $ 3.4 | 46 | 324 | 45% | 18% | 18.0 | 44.4 | 27.1 | $ 549 | $ 105 | $ 266 | $ 354 | $ 1,309 |
| Djibouti | LMIC | $ 3,150 | $ 35.6 | $ 15.4 | $ 3.5 | 44 | 309 | 27% | 12% | 20.9 | 48.1 | 25.1 | $ 323 | $ 103 | $ 127 | $ 354 | $ 1,242 |
| Bhutan | LMIC | $ 3,266 | $ 35.7 | $ 15.6 | $ 3.6 | 50 | 350 | 24% | 16% | 23.1 | 37.5 | 21.7 | $ 702 | $ 92 | $ 323 | $ 379 | $ 1,421 |
| Cabo Verde | LMIC | $ 3,293 | $ 35.8 | $ 15.7 | $ 3.6 | 51 | 356 | 27% | 18% | 21.1 | 23.6 | 16.9 | $ 2,749 | $ 79 | $ 454 | $ 413 | $ 1,448 |
| Bolivia | LMIC | $ 3,345 | $ 35.8 | $ 15.7 | $ 3.6 | 48 | 337 | 27% | 15% | 20.5 | 36.6 | 19.2 | $ 2,885 | $ 93 | $ 671 | $ 389 | $ 1,371 |
| Philippines | LMIC | $ 3,461 | $ 36.0 | $ 15.9 | $ 3.7 | 50 | 348 | 21% | 9% | 20.3 | 33.6 | 24.6 | $ 963 | $ 91 | $ 418 | $ 402 | $ 1,413 |
| Micronesia | LMIC | $ 3,571 | $ 36.1 | $ 16.1 | $ 3.8 | 47 | 328 | 43% | 11% | 21.7 | 38.0 | 28.9 | $ 638 | $ 114 | $ 1,353 | $ 415 | $ 1,329 |
| West Bank & Gaza | LMIC | $ 3,664 | $ 36.2 | $ 16.2 | $ 3.8 | 54 | 379 | 22% | 11% | 19.0 | 32.2 | 22.2 | $ 2,050 | $ 115 | $ 510 | $ 427 | $ 1,536 |
| Algeria | LMIC | $ 3,691 | $ 36.2 | $ 16.2 | $ 3.8 | 107 | 1,145 | 18% | 12% | 17.1 | 30.1 | 19.4 | $ 4,414 | $ 106 | $ 581 | $ 430 | $ 3,057 |
| Egypt, Arab Rep. | LMIC | $ 3,699 | $ 36.2 | $ 16.3 | $ 3.8 | 53 | 370 | 17% | 4% | 18.9 | 47.0 | 24.0 | $ 1,327 | $ 99 | $ 375 | $ 452 | $ 1,498 |
| Vietnam | LMIC | $ 3,756 | $ 36.3 | $ 16.3 | $ 3.9 | 43 | 307 | 28% | 9% | 19.4 | 27.5 | 19.6 | $ 2,235 | $ 111 | $ 423 | $ 436 | $ 1,237 |
| Morocco | LMIC | $ 3,795 | $ 36.3 | $ 16.4 | $ 3.9 | 51 | 356 | 22% | 10% | 19.5 | 31.4 | 21.1 | $ 1,399 | $ 113 | $ 491 | $ 441 | $ 1,446 |
| Tunisia | LMIC | $ 3,807 | $ 36.4 | $ 16.4 | $ 3.9 | 57 | 399 | 26% | 4% | 17.5 | 22.4 | 18.6 | $ 3,419 | $ 108 | $ 604 | $ 442 | $ 1,625 |
| Samoa | LMIC | $ 3,857 | $ 36.4 | $ 16.5 | $ 4.0 | 73 | 838 | 38% | 8% | 18.1 | 31.4 | 23.6 | $ 676 | $ 118 | $ 540 | $ 448 | $ 2,077 |
| Eswatini | LMIC | $ 3,978 | $ 36.6 | $ 16.7 | $ 4.0 | 51 | 357 | 30% | 26% | 20.5 | 49.7 | 24.5 | $ 2,043 | $ 172 | $ 596 | $ 463 | $ 1,461 |
| Sri Lanka | LMIC | $ 4,014 | $ 36.6 | $ 16.7 | $ 4.1 | 102 | 1,101 | 18% | 6% | 19.0 | 23.6 | 19.8 | $ 1,617 | $ 100 | $ 376 | $ 464 | $ 2,906 |
| Iran, Islamic Rep. | LMIC | $ 4,091 | $ 36.7 | $ 16.8 | $ 4.1 | 99 | 1,242 | 21% | 10% | 18.4 | 25.3 | 19.1 | $ 1,499 | $ 110 | $ 1,963 | $ 474 | $ 2,819 |
| Lebanon | LMIC | $ 4,136 | $ 36.7 | $ 16.9 | $ 4.1 | 514 | 5,330 | 10% | 8% | 19.1 | 20.4 | 18.7 | $ 3,284 | $ 113 | $ 3,885 | $ 480 | $ 14,623 |
| Indonesia | LMIC | $ 4,333 | $ 36.9 | $ 17.2 | $ 4.3 | 54 | 374 | 26% | 16% | 19.8 | 34.1 | 24.4 | $ 869 | $ 99 | $ 321 | $ 503 | $ 1,529 |
| El Salvador | LMIC | $ 4,551 | $ 37.2 | $ 17.5 | $ 4.4 | 53 | 369 | 27% | 4% | 21.4 | 22.1 | 16.7 | $ 4,006 | $ 117 | $ 1,201 | $ 542 | $ 1,506 |
| Mongolia | LMIC | $ 4,566 | $ 37.2 | $ 17.5 | $ 4.4 | 59 | 408 | 26% | 3% | 17.0 | 57.2 | 24.0 | $ 2,020 | $ 90 | $ 531 | $ 530 | $ 1,674 |
| Ukraine | LMIC | $ 4,836 | $ 37.5 | $ 17.8 | $ 4.6 | 59 | 417 | 26% | 19% | 16.0 | 35.4 | 16.9 | $ 3,058 | $ 119 | $ 771 | $ 561 | $ 1,691 |
| Burundi | LIC | $ 221 | $ 30.7 | $ 9.1 | $ 0.7 | 31 | 206 | 27% | 23% | 17.7 | 47.8 | 25.1 | $ 85 | $ 31 | $ 24 | $ 26 | $ 880 |
| Afghanistan | LIC | $ 369 | $ 31.1 | $ 9.7 | $ 0.9 | 37 | 285 | 32% | 17% | 18.7 | 70.0 | 28.2 | $ 51 | $ 38 | $ 172 | $ 42 | $ 1,042 |
| Somalia | LIC | $ 447 | $ 31.3 | $ 9.9 | $ 1.0 | 31 | 177 | 36% | 28% | 15.5 | 59.3 | 27.5 | $ 250 | $ 36 | $ 34 | $ 52 | $ 870 |
| Central African Rep. | LIC | $ 461 | $ 31.4 | $ 10.0 | $ 1.0 | 34 | 229 | 35% | 24% | 17.6 | 54.7 | 27.9 | $ 30 | $ 40 | $ 77 | $ 54 | $ 962 |
| Sierra Leone | LIC | $ 480 | $ 31.4 | $ 10.0 | $ 1.1 | 34 | 251 | 20% | 17% | 19.4 | 57.4 | 22.2 | $ 96 | $ 45 | $ 80 | $ 59 | $ 957 |
| Mozambique | LIC | $ 492 | $ 31.5 | $ 10.1 | $ 1.1 | 33 | 240 | 30% | 12% | 18.0 | 52.5 | 24.4 | $ 181 | $ 49 | $ 60 | $ 57 | $ 930 |
| Madagascar | LIC | $ 501 | $ 31.5 | $ 10.1 | $ 1.1 | 36 | 275 | 23% | 20% | 19.3 | 49.8 | 26.1 | $ 83 | $ 41 | $ 27 | $ 58 | $ 1,014 |
| Syrian Arab Rep. | LIC | $ 533 | $ 31.5 | $ 10.2 | $ 1.1 | 40 | 244 | 13% | 1% | 24.7 | 34.5 | 22.5 | $ 298 | $ 45 | $ 43 | $ 62 | $ 1,151 |
| Congo, Dem. Rep. | LIC | $ 577 | $ 31.6 | $ 10.3 | $ 1.2 | 31 | 212 | 23% | 17% | 15.2 | 46.2 | 24.0 | $ 41 | $ 45 | $ 33 | $ 67 | $ 880 |
| Niger | LIC | $ 591 | $ 31.7 | $ 10.3 | $ 1.2 | 34 | 234 | 18% | 19% | 14.7 | 67.4 | 22.9 | $ 119 | $ 45 | $ 61 | $ 69 | $ 953 |
| Eritrea | LIC | $ 614 | $ 31.7 | $ 10.4 | $ 1.2 | 31 | 181 | 29% | 22% | 20.3 | 46.2 | 27.0 | $ 127 | $ 48 | $ 38 | $ 75 | $ 873 |
| Malawi | LIC | $ 635 | $ 31.8 | $ 10.5 | $ 1.3 | 37 | 268 | 29% | 32% | 19.3 | 49.4 | 24.1 | $ 233 | $ 56 | $ 57 | $ 74 | $ 1,052 |
| Liberia | LIC | $ 676 | $ 31.9 | $ 10.6 | $ 1.3 | 35 | 248 | 22% | 28% | 19.3 | 43.4 | 22.4 | $ 36 | $ 47 | $ 111 | $ 78 | $ 982 |
| Chad | LIC | $ 686 | $ 31.9 | $ 10.6 | $ 1.3 | 38 | 294 | 21% | 24% | 15.8 | 64.5 | 22.5 | $ 122 | $ 46 | $ 61 | $ 80 | $ 1,082 |
| Yemen, Rep. | LIC | $ 702 | $ 31.9 | $ 10.7 | $ 1.4 | 18 | 148 | 15% | 19% | 19.6 | 54.8 | 24.6 | $ 110 | $ 50 | $ 61 | $ 70 | $ 520 |
| Sudan | LIC | $ 752 | $ 32.0 | $ 10.8 | $ 1.4 | 29 | 208 | 13% | 16% | 17.8 | 51.8 | 23.2 | $ 117 | $ 49 | $ 37 | $ 87 | $ 820 |
| Gambia, The | LIC | $ 772 | $ 32.1 | $ 10.8 | $ 1.4 | 40 | 328 | 21% | 23% | 18.3 | 39.2 | 21.2 | $ 359 | $ 54 | $ 28 | $ 90 | $ 1,134 |
| Guinea-Bissau | LIC | $ 795 | $ 32.1 | $ 10.9 | $ 1.5 | 40 | 302 | 34% | 39% | 19.7 | 45.6 | 25.4 | $ 78 | $ 46 | $ 123 | $ 92 | $ 1,132 |
| Rwanda | LIC | $ 822 | $ 32.2 | $ 11.0 | $ 1.5 | 37 | 290 | 24% | 18% | 19.3 | 46.1 | 22.2 | $ 274 | $ 60 | $ 113 | $ 95 | $ 1,040 |
| Mali | LIC | $ 874 | $ 32.3 | $ 11.1 | $ 1.6 | 41 | 327 | 16% | 19% | 16.2 | 65.6 | 20.7 | $ 92 | $ 55 | $ 62 | $ 102 | $ 1,172 |
| Uganda | LIC | $ 884 | $ 32.3 | $ 11.1 | $ 1.6 | 38 | 297 | 26% | 14% | 18.9 | 50.2 | 23.0 | $ 136 | $ 56 | $ 59 | $ 103 | $ 1,083 |
| Burkina Faso | LIC | $ 893 | $ 32.3 | $ 11.2 | $ 1.6 | 40 | 307 | 20% | 17% | 17.9 | 63.2 | 22.6 | $ 214 | $ 54 | $ 105 | $ 104 | $ 1,129 |
| Ethiopia | LIC | $ 925 | $ 32.4 | $ 11.2 | $ 1.6 | 34 | 484 | 26% | 15% | 19.1 | 48.3 | 22.2 | $ 279 | $ 58 | $ 48 | $ 107 | $ 961 |
| Togo | LIC | $ 973 | $ 32.5 | $ 11.4 | $ 1.7 | 38 | 285 | 23% | 11% | 18.6 | 45.3 | 24.0 | $ 231 | $ 65 | $ 104 | $ 112 | $ 1,070 |
| South Sudan | LIC | $ 1,072 | $ 32.6 | $ 11.6 | $ 1.8 | 17 | 101 | 24% | 17% | 20.1 | 59.8 | 22.6 | $ 157 | $ 63 | $ 57 | $ 125 | $ 486 |
| Zambia | LIC | $ 1,137 | $ 32.8 | $ 11.7 | $ 1.8 | 33 | 234 | 32% | 14% | 18.9 | 51.5 | 25.5 | $ 431 | $ 68 | $ 104 | $ 132 | $ 930 |
| Guinea | LIC | $ 1,189 | $ 32.8 | $ 11.9 | $ 1.9 | 45 | 360 | 26% | 14% | 18.2 | 55.3 | 21.5 | $ 194 | $ 63 | $ 88 | $ 138 | $ 1,268 |

*CFR = case fatality rate, GDP = gross domestic product (source: World Bank open data* [63]*), GI bleeding = gastro-intestinal bleeding, ICU = intensive care unit, PCR = polymerase chain reaction test, RDT = rapid diagnostic test (all testing costs here include both sample collection costs and medium test kit costs) LIC = low-income country, LMIC = lower middle income country, TB = tuberculosis, UMIC = upper middle income country, VT = venous thrombosis, YLL = year of life lost.*

** “Screen cost” corresponds to the costs of oral history as described in Section C.1 in S2 Appendix.*

## **C. Clinical screening and testing costs**

### **C.1. Clinical screening costs**

In order to treat at least some patients for COVID, they need to be clinically screened. Clinical screening for COVID is therefore only unnecessary if COVID is ignored entirely and only symptomatic treatment is provided (“testing and treating none”). However, even in this context, patients would be screened to assess whether they may have other diseases e.g., influenza or malaria, so some screening costs would still be incurred. The added costs when screening specifically for COVID would relate to signs/symptoms assessed in COVID screening but not relevant to other diseases that are routinely screened in the country. Given the range of diseases that can look like COVID in LMIC settings (e.g., influenza, pneumonia, RSV, TB, malaria, etc. [4]), it is unclear whether any added costs would be incurred at all. In this context, at baseline, we chose to use a $0 cost of screening while in sensitivity analysis, we used “oral history” costs as reflected in Torres-Rueda et al., 2021[64], using the average ratio of costs for Ethiopia, Pakistan and South Africa to estimated healthcare staff salaries [65] (0.0105) to extrapolate screening costs in other countries.

### **C.2. Testing costs**

There is a dearth of peer-reviewed studies having undertaken primary data collection on COVID RDT costs in healthcare settings. We have therefore used studies relying on either primary [66] or secondary [67] information (excluding personal communications). For PCR, we found five studies (from Brazil, Ethiopia, Ghana, India and Mozambique) with partial or comprehensive primary and secondary data [66–70]. All these studies are presented in S4 Table.

**S4 Table: Costing studies used to extrapolate costs**

| **Reference** | **Country** | **Income level** | **Health worker salary** | **% of health worker salary** | **Type** | **Cost in US$** | **Perspective** | **Staff** | **Supplies, tests** | **Test kit** | **Other supplies** | **Overhead + capital** |
| --- | --- | --- | --- | --- | --- | --- | --- | --- | --- | --- | --- | --- |
| Manjate et al., 2023 [66] | Mozambique | LIC | 136 | 1.396% | RDT | $11.4* ($8.9-13.0) | HS | 1.9 | 7 | 4.6 (2.1-6.2) | 2.4 (2.3-2.5) | 2.6 |
| Cedro et al., 2023 [67] | Brazil | UMIC | 810 | 0.720% | RDT | $14.18 | Provider | 5.83 | NA | NA | 0.6 | NA |
| Manjate et al., 2023 [66] | Mozambique | LIC | 136 | 1.984% | PCR | $39 | HS | 2.7 | 34.5 | 33.4 | 1.1 | 2 |
| Yigezu et al., 2022 [69] | Ethiopia | LIC | 212 | 1.447% | PCR | $5.24 | Partial costs** | 3.07 | 0.67 | NA | NA | 1.5 |
| Ismaila et al., 2021 [68] | Ghana | LMIC | 390 | NA | PCR | $63.50 | HS | NA | NA | NA | 0.6 | NA |
| Cedro et al., 2023 [67] | Brazil | UMIC | 810 | 0.720% | PCR | $37.63 | Provider | 5.83 | NA | NA | 0.6 | NA |
| Minhas et al., 2023 [70] | India | LMIC | 337 | 0.282% | PCR | $9.20 | Provider | 0.95 | 8.03 | 7.13 | 0.9 | 0.25 |

*Notes: HS = health system, LIC = low-income country, LMIC = lower-middle-income country, NA = not applicable, PCR = polymerase chain reaction, RDT = rapid diagnostic test, UMIC = upper-middle-income country. Health worker salaries were estimated based on Serje et al., 2018* [65]*. Not all papers provided the details of all the costs, hence only some of the costs were usable for extrapolations. Cost computations were made using data from 5 sites for Manjate et al., 4,305 diagnostic tests for Cedro et al., 73,955 tests for Yigezu et al., 56,318 tests for Minhas et al., while for Ismaila et al., estimates were protocol-based hence no patients were actually enrolled in the study.*

** Adjusted for a conversion mistake in the logistics costs for the COVIOS test. ** Yigezu et al. focused on sample collection, laboratory and contact tracing costs.*

We extrapolated RDT and PCR testing costs by dividing countries into two income groups: low- and lower-middle income, and upper-middle income countries (this ensured that there was at least one study in each country group), and proceeding as follows:

- We assumed that, within each country grouping, staff costs were proportional to estimated health worker salary costs [65]. We therefore computed the ratio of staff costs to estimated salary costs for studies with data, averaged these values (if more than one was available) within a given country income group, and used the resulting parameter for extrapolation to other countries.
- We assumed that ‘other supplies’ (excluding test kits) costs were constant in 2021$ (tradables). Costs were updated to 2021$ using the US deflator [63] then, similarly to what was done with staff costs, we averaged available values within the same income group (if more than one value was available) then used the resulting amount as the estimated cost of ‘other supplies’ in other countries.
- For test kit costs (treated as tradables like “other supplies”), we mobilized the sources presented in the table above as well as external sources:
  - For RDT test kits: we used a medium price of $2 and $0.6-6.2 range, reflecting the Global Fund list price [71] and spread of values identified in Mozambique [66] (median $4.6, with different tests costing $2.1, $4.6 after correction for a conversion mistake for the logistics costs of the COVIOS test, $5, $5 and $6.2), Peru [4] ($4.9), South Africa [72] ($2.9) and Thailand [73] ($1.6).
  - For PCR test kits: we used a lognormal distribution with average $27.2 and shape 0.79. This was set to reflect figures in different studies, including: $2.8 [74] from UNICEF, a $10-17 reference price [75], $7.5 in India [70], $34.9 in Mozambique [66] and around $50-60 in Ghana and Uganda [68,76].
- We would normally estimate capital and overhead costs (non-tradables costs) assuming they are proportional to GDP/capita PPP. However, extrapolating the only data point we had [66] for RDTs, to e.g., Germany would have resulted in $113 overhead/capital, in contrast with $4 found in a costing study [77]. We therefore chose to rather estimate overhead and capital costs as a proportion of “other testing costs” (29.5% in Mozambique and 12.7% in Germany), and used an average of 21.1% in this paper. Overhead/capital represented a smaller proportion of other PCR testing costs because of the high cost of test kits: 5.4% (excluding studies from a provider perspective). This share was applied to baseline kit costs.
- Finally, we estimated uncertainty around our predictions so that it would best reflect the differences between predicted and actual non-test kit costs in studies for which we have some data, and assuming that non-test kit costs followed a lognormal distribution. We consequently identified the shape parameter of the distribution (shape = 0.70).

The resulting testing costs are an acceptable fit for existing data from available studies but should be improved upon as more data become available.

## **D. Costs of generic treatment side effects**

### **D.1 Cost of bone fracture treatment**

Corticosteroids can lead to an increased risk of fracture, even when taken only for a short period of time (around 0.12% added risk following short-course treatment) [25]. The mechanisms through which they drive fracture risk may include increased osteoporosis and muscle weakness [27]. Associated costs are twofold: DALYs lost through increased death/disability, and fracture treatment costs. The latter are highly dependent on the country, type of fracture and severity. We sought to develop broad ranges that can help discuss the influence these costs have on the results of the model. Any country-specific discussion should use contextualized costs. To estimate costs:

- We computed an ‘expected’ value of fracture costs associated with corticosteroids by combining an estimate of the relative expected prevalence of different types of fractures (20.6% hip, 4.6% forearm, 33.8% vertebral 41.0% other among additional fractures associated with the corticosteroid doses involved in COVID treatment) [78] with the median costs for these fracture types by income range (low-, lower-middle- and upper-middle-income) - see S5 Table. We lack data for hip, forearm and spine fractures in low-income countries (LICs) hence have used lower-middle-income country median values instead. Estimated expected costs of fractures are $1,770 for LICs, $1,893 LMICs and $2,927 in UMICs respectively.
- We then assessed how results would change using the extreme ends of the ranges of the cost of fracture treatment at each income level. Cost ranges are: $31-749 per fracture in LICs, $90-5,938 in LMICs, and $105-14,764 in UMICs.

**S5 Table: Costs of different types of fractures across various low-and-middle-income countries**

| **Income level** | **Fracture type** | **Number of studies** | **Cost (median & range)** | **Countries and sources** |
| --- | --- | --- | --- | --- |
| Low income | Forearm | 0 | NA | NA |
| Low income | Hip | 0 | NA | NA |
| Low income | Vertebra | 0 | NA | NA |
| Low income | Other fracture | 4 | $ 497 [$31, $745] | Malawi [79,80], Uganda [81–83] |
| Lower middle income | Forearm | 2 | $ 794 [$567, $1,021] | Iran [84,85] |
| Lower middle income | Hip | 3 | $ 4,220 [$3,343, $4,389] | Cabo Verde [86], Iran [84,85] |
| Lower middle income | Vertebra | 2 | $ 1,905 [$1,255, $2,556] | Iran [84,85] |
| Lower middle income | Other fracture | 11 | $ 719 [$210, $14,640] | Cambodia [87], Iran [84,85], Kenya [88] , Nigeria [89,90], Papua New Guinea [91], Senegal [92], Tanzania [93–95] |
| Upper middle income | Forearm | 2 | $ 1,686 [$128, $3,244] | Brazil [96], Mexico [97] |
| Upper middle income | Hip | 12 | $ 6,348 [$1,328, $10,420] | Brazil [98,99], China [100–103], Mexico [97,104,105], South Africa [106], Thailand [107], Turkiye [108] |
| Upper middle income | Vertebra | 4 | $ 2,399 [$353, $4,321] | Brazil [96], China [101,102], Mexico [97] |
| Upper middle income | Other fracture | 6 | $ 1,586 [$78, $7,603] | Brazil [96], China [101,102], Mexico [97,109], Turkiye [110] |

### **D.2 Cost of gastro-intestinal bleeding treatment**

We searched for studies estimating the costs of treating gastro-intestinal (GI) bleeding in LMICs (S6 Table). In Thailand, the cost of treating upper GI bleeding in 2009-2010 was derived from inpatient medical expense forms and amounted to 2010 $561, with large differences between variceal ($1460) and non-variceal ($481) bleeding [111]. The cost of treating cirrhosis with GI bleeding vs. uncomplicated cirrhosis in the Congo in 1999-2000 [112] was 375.08 vs. 313.90 euros or an additional cost of 2000 $56 associated with GI bleeding treatment (approximately 80% of it likely being upper GI bleeding [113]). This is probably an under-estimate of the cost of treating an episode of GI bleeding without cirrhosis in the Congo as the number of days of hospitalization in the study is similar for uncomplicated cirrhosis vs. cirrhosis with GI bleeding, yet isolated episodes of GI bleeding are associated with several days of hospitalization [111]. Another study in Malaysia found a cost of treating upper GI bleeding of 2014 US$ 490 [114] but that study was looking into a range of effects and only one patient had GI bleeding. Finally, a Chinese study [115] found that treating major GI bleeding cost $ 2,962, likely an over-estimate of the cost of treating corticosteroid-induced GI bleeding because of the study’s exclusive focus on major episodes. The estimates of the costs of treating GI bleeding in the LMIC studies have further been limited to a single episode, while information from UK [116] and US [117] studies suggests that 16-40% of costs are incurred after the initial hospitalization (including through bleeding recurrence). LMIC estimates also relate exclusively or mostly to upper GI bleeding, even though lower GI bleeding is expected to represent around 20-30% of GI bleeds [31]. However, a United States modelling study [118] suggests that the average costs of upper and lower GI hemorrhage are similar. Finally, collected costs focused on direct costs, even though overhead costs may be substantial. For example, in (Bai and Zare, 2020) [119], overheads and capital represented 48% of total costs, so total costs are substantially under-estimated in LMIC studies.

**S6 Table: costs of gastrointestinal (GI) bleeding treatment in low-and-middle-income countries**

| **Source** | **Bleed type** | **Country** | **Income level** | **Type of costs included** | **Cost** | **Cost year** | **Comments** |
| --- | --- | --- | --- | --- | --- | --- | --- |
| Sangchan et al., 2012 [111] | UGIB | Thailand | UMIC | Inpatient costs | $ 561 | 2010 | Costs derived from inpatient medical expense forms |
| Atipo-Ibara et al., 2004 [112] | Cirrhosis bleeding* | Congo | LIC | In hospital direct costs | > $ 56 | 2000** | Difference between costs of cirrhosis with bleeding and cirrhosis without, data based on existing tariffs |
| Pok et al., 2018 [114] | UGIB | Malaysia | UMIC | Direct healthcare costs | $ 490 | 2014 | Primary data collection but one case only |
| Chang et al., 2018 [115] | Major GI bleeding | China | UMIC | In hospital direct costs | $ 2,962 | 2014 | Focuses on major bleeding episodes only |

*LIC = low-income country, LMIC = lower-middle-income country, UGIB = upper GI bleeding, UMIC = upper-middle-income country. * 80% are expected to be related to upper GI bleeding*[113]*. ** The year was not specified in the paper: this is the latest year of data collection. All other costs are specified in dollars of the year specified under “cost year”.*

We decided, for the purpose of giving a broad estimate of the cost of treating GI bleeding, to 1) consider treatment costs as non-tradables based on studies showing tradables represent a minority of total costs [115,116] for the purpose of translating costs from one country/year to another, 2) base our baseline country-specific cost-estimates on the Thai study [111], and use the Congo and Chinese studies to develop low and high country-specific estimates, 3) add 40%, 20% and 67% of initial hospitalization costs (i.e., 28.5%, 17% and 40% of total costs) to reflect costs incurred after the initial hospitalization (in particular readmission) in baseline, low and high estimates respectively, 4) add overhead costs, estimated at 92% of costs minus overheads or 48% of total costs.

### **D.3 Cost of venous thrombosis treatment**

We found three recent (data from 2015 or later) studies in LMICs assessing the cost of venous thrombosis treatment. These costs varied between 2017 US$ 4744-5572 in China [120], 2021 US$4061-6363 in Thailand [121,122] and 2021 US$989-933 in Ethiopia [123]. Treatment costs are dominated by hospitalization costs [120] hence we expressed the cost of treating one venous thrombosis case as a proportion of estimated hospital bed-day costs [64] in upper middle income countries, on the one hand (using China and Thailand as a basis for estimates), and low and lower-middle income countries, on the other hand (using Ethiopia results as a basis for estimates). We assumed that treatment costs followed a log-normal distribution and estimated within and between country variability in the ratio of treatment costs to estimated hospital bed-day costs to feed into uncertainty estimates.

### **D.4 Cost of sepsis treatment**

We first estimated immediate treatment costs for the sepsis episode. We used rough estimates based on:

- A systematic review (Salman et al., 2020 [124]) of the cost of sepsis in neonates and infants.
- A later study, which found on the cost of sepsis in neonates in Mozambique and South Africa [125].
- Two studies on the cost of sepsis in older patients in Vietnam (added costs of post-operative sepsis: 2018 US$724) [126] and Brazil (mean cost of treating sepsis: 2015 US$ 624) [127].

While the costs of treating neonates and adults are different, for the purpose of a broad order of magnitude, we combined child and adult estimates to produce country-specific costs and uncertainty intervals.

### **D.5 Cost of heart failure treatment**

We build an estimate of the costs of treating heart failure (HF) in LMICs using the following studies:

- A series of studies setting the lifetime cost of treating HF at 2021 US$ 307 to $4,761 in Thailand depending on the type of HF (with preserved or reduced ejection fraction, each of these representing around half of the cases [128]) and treatment choice[129,130]. Costs were discounted using a 3% discount rate. With preserved and reduced ejection fraction representing around half of cases each [128], the minimum cost of treating HF in Thailand is $473.2 per case.
- A series of studies estimating costs for patients with reduced ejection fraction only at: $3,671 and $4,377 in China, $3,887 in Colombia, $7,572 in Egypt [131–137].
- A study on yearly costs in Nigeria, which found 2016 US$2,343 HF treatment costs including patient costs (56% of all costs) [138] and a case fatality rate in the first year around 1 in 6[139]. Assuming as a rough estimate a life expectancy of 5.54 years [41] at the same cost, with 3% discounting, the corresponding lifetime cost would be around $14,000, 2021$7,263 from a healthcare perspective.

The cost of treating HF is highly dependent on the treatment chosen and it was difficult to assess how average costs may vary depending on a country’s income level. Hence, for the purpose of this paper, we used a flat lifetime cost of $4,023 per heart failure case at baseline for all LMICs, and explored a range of $473 to $7,572 in sensitivity analysis.

### **D.6 Costs of treating for the mild side effects of IL-6 receptor blockers (TCZ)**

Mild side effects of IL-6 receptor blockers range from nausea and diarrhea to (less commonly) URTIs [21]. Associated treatment may include, among others, paracetamol, antacids, antibiotics, at different doses, or no treatment and all. The cost of 10 days of treatment for an adult (using the higher end of the dose range) for some of the possible treatment options are:

- Anti-nausea medication (metoclopramide) costs per patient for 10 days (30 times 10 mg): $0.39 in the UK, $0.12 in South Africa and India, and $1.2 (generic) [57].
- Paracetamol costs per patient for 10 days (60 times 500 mg): $0.8-3.2 in Uganda [140], $ 0.3, 0.18, 0.24 and 0.96 in South Africa, India, the UK and for the generic price respectively [57].
- Antibiotic costs: see above (median: $0.7 and range $0.1-5).

For the purpose of this model, we assumed around half of the patients with nausea and most patients with URTI were treated and build overall treatment costs using their respective population among patients treated with TCZ.

## **E. Disease-specific corticosteroid side effects**

Corticosteroids are recommended against for patients with influenza [141], recommended for patients with pneumocystis pneumonia [142], tuberculosis of the central nervous system and pericardial tuberculosis [143], severe typhoid [144] and croup (parainfluenza) [145]. They have been determined to be either ineffective or detrimental when used to treat malaria [146], are not recommended to be routinely offered for community-acquired pneumonia [147,148] and are not indicated for hospitalized RSV patients [149]. Evidence for the use of corticosteroids with dengue [150], rhinovirus or adenovirus [147,151,152] is insufficient to draw guidelines. When impacts are proven or suspected, we provide available estimates in S7 Table. Given that corticosteroids are recommended for COVID patients with low oxygen, if oxygen levels are measured accurately and guidelines followed, corticosteroid administration to non-COVID patients would primarily concern diseases most likely to lead to low oxygen i.e., influenza, other respiratory infections, and pulmonary TB. Some additional illnesses may, in some cases, be associated with oxygen needs e.g. cerebral malaria [153,154] or severe sepsis [155]. However, this is not the most common presentation of the disease and the prevalence of those presentations among severe, COVID-like patients is hard to quantify with precision. In this paper, corticosteroid side-effect estimates were based on the assumption that COVID-like, non-COVID patients with severe presentations and oxygen needs would have upper- or lower-respiratory infections or tuberculosis (classified separately in the Global Burden of Disease list of causes of deaths or DALYs). These diseases were also the ones included in estimates of YLLs associated with non-COVID, COVID-like deaths. We undertook some sensitivity analyses to estimate how YLLs may change if we added patients with diseases sometimes associated with a respiratory presentation and a need for oxygen, even if this is uncommon (e.g., malaria, sepsis), and found that the difference would not be substantial in most countries (around 2% on average).

**S7 Table: Disease-specific side effects of corticosteroid use**

| **Disease** | **Direction of impact** | | **Details** |
| --- | --- | --- | --- |
| Influenza | Suspected negative | Adjusted OR of death: 2.46 (95% CI 1.49,4.06), aHR: 1.32 [0.95,1.85] [141]  Increase in length of hospital stay [141]: 3.15 [2.19 , 4.10] days (survivors), 1.54 [-0.02 , 3.11] (cases with respiratory failure, survivors)  Baseline risk of mortality for severe influenza: range from close to 0 to almost 30% [156,157]. | |
| Tuberculosis | Positive for some TB types | Lower mortality reported for specific types of TB:   - Lower incidence of mortality for tuberculosis of the CNS associated with dexamethasone use: OR 0.79 (95% CI 0.61 to 1.02) [143] (Note: TB of the CNS represents close to 10% of hospitalized TB cases, which have high mortality [158]). - Lower incidence of mortality for pericardial tuberculosis associated with corticosteroid use: OR 0.70 (95% CI 0.45 to 1.08) [143] (Note: pericardial TB has high mortality: 17-60% [159] but is rare overall [160]). | |
| Severe typhoid | Suspected positive | Estimated RR of mortality: 0.19 [0.02, 1.04] [161]  Baseline mortality: range: 35-55% [161] | |
| Croup | Positive | Glucocorticoids standard treatment for croup, reduces croup score [162]  Reduced hospital stay by: 8.49 [1.76 , 3.23] hours in children [163] | |
| Pneumocystis pneumonia | Positive | Part of standard treatment: OR of death: 0.54 [0.38, 0.79] for HIV pneumocystis pneumonia, 0.63 [0.41-0.95] for non-HIV pneumocystis pneumonia with hypoxemia and 0.69 [0.47, 1.01] for non-HIV pneumocystis pneumonia with respiratory failure [164]^28^  Baseline mortality: in [165], around 10% for treated HIV pneumocystis pneumonia patients and 30% if the patients are untreated. | |

**aHR = adjusted hazard ratio, CI = confidence interval, CNS = central nervous system, HIV = human immunodeficiency virus, OR = odds ratio, RR = risk ratio, TB = tuberculosis.*

In this paper, we further chose to focus on the impact of combined COVID and influenza transmission, including the possibility of a “twindemic” [166,167] of influenza and COVID, and did not focus on the potential impact of corticosteroids on other lower- and upper-respiratory infections or tuberculosis. This was motivated by the fact that influenza was the most commonly mentioned “COVID-like” disease in consultations with LMIC experts [4], the magnitude of suspected health impacts, and the relative ease in quantifying those impacts. GBD datasets on deaths and YLL per disease [168] suggest that influenza may represent 10% to 66% of COVID-like respiratory deaths (including lower- and upper-respiratory infections and tuberculosis deaths) in any given year. Data on influenza prevalence among patients with COVID-like illness in LMICs is limited, but it was, for example, 14.5% [12, 17%] (116/732) in Peru between June 1- Sept 30 2023 [169], confirming that influenza can be common in patients with COVID-like illness.. In this paper, we have decided to consider a range of 0-30% influenza prevalence among COVID-like, non-COVID severe patients, and have focused on two main scenarios in the main manuscript: 1% and 10% influenza in severe COVID-like non-COVID patients. We provide results for further scenarios in Section A in S3 appendix. We assumed a baseline risk of death among severe influenza cases needing oxygen (those are the ones that may be treated with corticosteroids) of 15%, and a risk ratio of mortality in influenza cases treated with corticosteroids of 1.67 (approximately a mid-point between available estimates). This leads to an added risk of mortality for an influenza patient treated with corticosteroids of 10 percentage points. In contexts in which disease-specific side-effects on other diseases may be important, however, the estimates presented in this paper may still be used by recognizing that what drives the results is the added mortality risk in severe COVID-like, non-COVID patients given corticosteroids. The scenarios that were described as a 1%, 10% or 30% influenza are indeed equivalent to an added mortality risk of 0.1%, 1% and 3% in severe non-COVID, COVID-like patients treated with corticosteroids, whatever the disease driving that increase may be.

## **F. Post-COVID syndrome**

Post-COVID is taking a substantial toll on former COVID patients. Should the use of corticosteroids and/or TCZ in severe COVID patients change the probability or severity of long-COVID, it may change the cost-effectiveness of testing. However, a quick literature review found mixed results (S8 Table). It is plausible that the impact that COVID treatment has on later symptoms may differ according to the nature of symptoms: for example, some studies found reduced odds of neurosensory impairment [170] or cardiovascular symptoms [171], while Huang et al. 2022 [172] found increased risks of muscle weakness though Bek et al., 2022 [173] found decreased risks. Treatment impacts could also be affected by the timing of the assessment (the time point at which symptoms were assessed in the table ranges from 6 weeks to 2 years, with post-COVID syndrome definition generally requiring symptoms for at least 3 months), and practices in terms of corticosteroid use [174]. In particular, the two studies with the largest, negative impact of corticosteroid use on long-COVID [174,175] reflect high levels of corticosteroid misuse (corticosteroid treatment of patients not needing any form of oxygen supplementation, a practice advised against in current guidelines [11]). Finally, in integrating long-COVID within estimates, it will be important to ensure there is no double counting between the generic side effects of corticosteroids estimated above and the disease-specific impact of corticosteroids on long-COVID.

**S8 Table: Link between corticosteroid use and long-COVID**

| **COVID cases in analysis** | **Severity of the target population**  **(respiratory support = O_2_, hospitalization = H)** | **Long-COVID cases** | **Number treated with steroids** | **Impact of corticosteroid use during the acute COVID phase (adjusted odds ratio)** | **Delay from diagnosis or discharge to assessment** | **Source** |
| --- | --- | --- | --- | --- | --- | --- |
| 1192 | 897 O_2_, 1192 H | 650 | 295 | 1.19 [0.99, 1.43] | 2-year | Huang et al., 2022 [172] |
| 504 | 217 H, 40 ICU | 227 | 102 | 2.13 [1.16, 3.98] | 12 weeks | Alghamdi et al., 2022 [176] |
| 396 | 74 O_2_,40 H | 347 | 179 | 4.08 [1.42, 11.70]* | Varied | Abdelhafiz et al., 2022 [174] |
| 123 | 123 H | 33 | 73 | 0.32 [0.11, 0.90] | 6 months | Davelaar et al., 2023 [177] |
| 120 | 49 O_2_, 120 H | 73 | 60 | 4.43 [1.9, 10.28]** | 6 weeks | Nair et al., 2023 [175] |
| 1966 | 542 moderate, 343 severe | 728 | ? | Lower symptom duration with steroids & TCZ*** | 1 year | Caguana Vélez et al., 2022 [178] |
| 572 |  | NA | 323 | Lower performance |  | Mastrorosa et al., 2023 [179] |

** The authors commented that the results may be driven by widespread unnecessary use of corticosteroids. Note that this study relied on an online survey for data collection.*

*** Results may be driven by mild patients treated with corticosteroids as all but two moderate/severe patients received corticosteroids.*

**** Results presented as a conference abstract, some information is missing.*

At this stage, and given ambiguity on results, we have chosen not to reflect the impact of COVID treatment on long-COVID at baseline. We however include it in sensitivity analysis, exploring the impact of an increase or reduction in the DALYs associated with long-COVID by 20% following treatment by either corticosteroids and/or corticosteroids + TCZ.

Such an impact could be significant. Bowe et al., 2023 [22] estimated the burden of long-COVID in formerly hospitalized COVID patients as 0.6428 (95% CI: 0.5969–0.6893) DALYs per patient (cumulative DALYs 2-years post COVID). Since symptoms persisted in at least some patients at the 2-year mark, with 59.4 DALYs incurred during the last 180 days of the 720 days period of assessment, total DALYs associated with post-COVID symptoms are likely to be higher, though it is unclear by how much. The study included patients treated in US hospitals from 1 March to 31 December 2020, who may or may not have received corticosteroids. To assess the baseline DALY weight of long-COVID in patients who did not receive corticosteroids or TCZ, we need to know what proportions were treated. TCZ was not much used during the period (5-6% during March-May[180] with a drop in June leading to a likely use in less than 1% of patients [181]). With TCZ not approved as a COVID treatment until 2021, we assumed we could neglect its use in the study cohort during the period. On the other hand, corticosteroid use was already important during March-May 2020 (21.5% over the period[180]), had grown sufficiently by June to strain supply [182], and was used in 75% of UK cases after its approval [183]. Assuming 54% use in June and 65-85% use afterward in the US, and accounting for the relative number of cases in each month [12], we estimated corticosteroid use with the cohort in Bowe et al., 2023, at 65% [57%, 73%] [22].

Cutler, 2022 [184] suggests that, in the US, medical costs for long-COVID may amount to around $5,700/person. Studies in Israel and the UK have however suggested more modest health system cost impacts: in Israel [185], post-COVID has been estimated to lead to excess spending of around $100/patient, and in the UK, a study of non-hospitalized COVID patients suggests that long-COVID costs may amount to £2.44/patient/year [186]. Limited information is available in LMICs, but we would assume that medical costs are likely to be substantially lower, as most patients may remain untreated. We therefore chose to neglect those costs. They could be added should more information become available and are integrated into the model code (with current value fixed at $0).

## **G. Estimated years of life lost per COVID death**

Arolas et al., 2021 [20] have estimated the number of years of life lost per COVID death in 81 countries at different income levels. To build estimates of YLLs per COVID death for other countries for the purpose of this model, we fitted a regression model using Arolas et al.’s YLL figures (dependent variable) and the following predictive variables: share of population in different age ranges (from World Population Prospects [187]), and GDP per capita (as per World Bank data [63]). The final model is as follows:

COVID YLL = 50.02 - 132.6 × (share of 0-4 years old population) - 44.4 × (share of 30-34 years old population) – 81.14 × (share of 50-59 years old population) – 84.31 × (share of 70+ years old population) – 0.00005455 × GDP/capita

## **H. Choice of sensitivity analyses**

**S9 Table: Options explored in sensitivity analysis**

| **Area** | **Changes in sensitivity analysis** | **Rationale** |
| --- | --- | --- |
| Treatment availability | Mechanical ventilation (MV) unavailable | Insufficient MV availability for many LMIC care centers [4]. |
| Treatment side effects | Treatment side effects/severe side effects ignored | Comparison with options in which side effects are included, to highlight the role that side effects play in the cost-effectiveness of testing |
| Treatment side effects | Low/high estimates for the costs of “generic” severe corticosteroid treatment side effects explored | Uncertainty and need for country-specific values to guide country decision-making. |
| Treatment side effects | Disease-specific corticosteroid side effects included (1% and 10% influenza prevalence in severe COVID-like, non-COVID patients) | Illustrate the impact of concurrent COVID and influenza waves (see Section E in S2 Appendix for more details). |
| Treatment side effects | Positive and negative impacts of COVID treatment on post-COVID DALYs | Post-COVID may be worsened or improved by the use of COVID treatment in severe patients, though the existence, direction and magnitude of that impact are unclear (see Section F in S2 Appendix). |
| Treatment costs | Wide range of TCZ costs explored and estimates of the maximum cost for which TCZ treatment would still be cost-effective | Importance of TCZ prices on treatment accessibility in LMICs. |
| Screening and testing costs | Clinical screening cost differential between testing options included | If standard clinical screening is insufficient to determine if a patient is a suspected COVID case, identification of patients who should be offered a test will involve additional screening costs (see 2.3.1 for the justification). |
| Screening and testing costs | Varying the cost of RDT test kits | Using low and high estimates of the cost of RDT test kits. This is the element of cost decision-makers are most likely to be able to choose or influence. We also chose to assess results should test kits be free (e.g., paid for/highly subsidized by donors), to explore whether this could substantially shift model results for poorer countries. |
| Test sensitivity | Lower and higher RDT sensitivity (60%, 40% and 90% vs. 80% at baseline) | RDT test sensitivity should be ≥ 80% (WHO guidance). However, individual test sensitivity varies. Further, changing variants have been associated with different sensitivity [188], suggesting that the choice of a given test sensitivity today does not guarantee that it will hold true with future variants. |
| Share not treated | Fewer positives effectively treated (70% & 40% vs. 95% at baseline) | Experts' feedback highlighting high treatment refusal (estimated at 60%) in at least one country at the height of the pandemic. |
| Health discounting | Discounting of health impacts (3% vs. 0% at baseline) | As per WHO guidelines [189]. |

*DALY = disability adjusted live years, LMIC = low and middle income country, RDT = rapid diagnostic test, TCZ = tocilizumab, WHO = World Health Organization*

# **References**

1. Bresser M, Erhardt RM, Shanaube K, Simwinga M, Mahlatsi PA, Belus J, et al. Evaluation of COVID-19 antigen rapid diagnostic tests for self-testing in Lesotho and Zambia. PLoS One. 2024 Feb 29;19(2):e0280105.

2. Qi J, Tan JN, Hui SH, Lim NC, Lau T, Haroon S. The Implementation and Role of Antigen Rapid Test for COVID-19 in Hemodialysis Units. Int J Environ Res Public Health. 2022;19(22).

3. Prazuck T, Gravier A, Pires-Roteira D, Theillay A, Pallay S, Colin M, et al. Evaluation of a new “all in one” SARS-CoV-2 antigen-detecting rapid diagnostic test and self-test: Diagnostic performance and usability in child and adult populations. J Med Virol. 2022 Sep 1;94(9):4097–106.

4. Bonnet G, Bimba J, Chavula C, Chifamba HN, Divala T, Lescano AG, et al. “We usually see a lot of delay in terms of coming for or seeking care”: an expert consultation on COVID testing and care pathways in seven low- and middle-income countries. BMC Health Serv Res. 2023 Nov 23;23(1):1288.

5. Use of SARS-CoV-2 antigen-detection rapid diagnostic tests for COVID-19 self-testing - Interim guidance [Internet]. Geneva: World Health Organization; 2022 [cited 2023 Jul 5]. Report No.: WHO/2019-nCoV/Ag-RDTs/Self_testing/2022.1. Available from: https://www.who.int/publications/i/item/WHO-2019-nCoV-Ag-RDTs-Self_testing-2022.1

6. Bekliz M, Adea K, Olha P, Perez-Rodriguez Francisco, Marques Melancia Stéfane, Baggio Stephanie, et al. Analytical Sensitivity of Eight Different SARS-CoV-2 Antigen-Detecting Rapid Tests for Omicron-BA.1 Variant. Microbiol Spectr. 2022 Aug 8;10(4):e00853-22.

7. Kostoulas P, Eusebi P, Hartnack S. Diagnostic Accuracy Estimates for COVID-19 Real-Time Polymerase Chain Reaction and Lateral Flow Immunoassay Tests With Bayesian Latent-Class Models. Am J Epidemiol. 2021;190(8):1689–95.

8. Mistry DA, Wang JY, Moeser ME, Starkey T, Lee LYW. A systematic review of the sensitivity and specificity of lateral flow devices in the detection of SARS-CoV-2. BMC Infect Dis. 2021 Aug 18;21(1):828.

9. Cornette M, Decaesteker B, Martens GA, Vandecandelaere P, Jonckheere S. From Delta to Omicron SARS-CoV-2 variant: switch to saliva sampling for higher detection rate. J Clin Virol Plus. 2022 Aug;2(3):100090.

10. Whittaker C, Watson OJ, Alvarez-Moreno C, Angkasekwinai N, Boonyasiri A, Carlos Triana L, et al. Understanding the Potential Impact of Different Drug Properties on Severe Acute Respiratory Syndrome Coronavirus 2 (SARS-CoV-2) Transmission and Disease Burden: A Modelling Analysis. Clin Infect Dis. 2022 Aug 24;75(1):e224–33.

11. Therapeutics and COVID-19: Living guideline, 13 January 2023. [Internet]. Geneva: World Health Organization; [cited 2023 May 7]. Available from: https://www.who.int/publications/i/item/WHO-2019-nCoV-therapeutics-2023.1

12. Institute for Health Metrics and Evaluation (IHME). COVID-19 Projections [Internet]. University of Washington, Seattle, USA; 2022 [cited 2022 Jan 6]. Available from: https://covid19.healthdata.org

13. Update to living systematic review on drug treatments for covid-19. BMJ. 2022 Jul 13;378:o1717.

14. Chang R, Elhusseiny KM, Yeh YC, Sun WZ. COVID-19 ICU and mechanical ventilation patient characteristics and outcomes-A systematic review and meta-analysis. PLoS One. 2021;16(2):e0246318.

15. Rees EM, Nightingale ES, Jafari Y, Waterlow NR, Clifford S, B Pearson CA, et al. COVID-19 length of hospital stay: a systematic review and data synthesis. BMC Med. 2020 Sep 3;18(1):270.

16. Alimohamadi Y, Yekta EM, Sepandi M, Sharafoddin M, Arshadi M, Hesari E. Hospital length of stay for COVID-19 patients: a systematic review and meta-analysis. Multidiscip Respir Med. 2022 Jan 12;17(1):856.

17. Salomon JAP, Vos TP, Hogan DRP, Gagnon MMS, Naghavi MP, Mokdad AP, et al. Common values in assessing health outcomes from disease and injury: disability weights measurement study for the Global Burden of Disease Study 2010. Lancet, The. 2012;380(9859):2129–43.

18. Liu Y, Sandmann FG, Barnard RC, Pearson CAB, Pastore R, Pebody R, et al. Optimising health and economic impacts of COVID-19 vaccine prioritisation strategies in the WHO European Region: a mathematical modelling study. The Lancet regional health Europe. 2022;12:100267–100267.

19. Haagsma JA, Maertens de Noordhout C, Polinder S, Vos T, Havelaar AH, Cassini A, et al. Assessing disability weights based on the responses of 30,660 people from four European countries. (1478-7954 (Print)).

20. Pifarré i Arolas H, Acosta E, López-Casasnovas G, Lo A, Nicodemo C, Riffe T, et al. Years of life lost to COVID-19 in 81 countries. Sci Rep. 2021 Feb 18;11(1):3504.

21. GBD 2019 Diseases and Injuries Collaborators. Global burden of 369 diseases and injuries in 204 countries and territories, 1990–2019: a systematic analysis for the Global Burden of Disease Study 2019. The Lancet (British edition). 2020;396(10258):1204–22.

22. Bowe B, Xie Y, Al-Aly Z. Postacute sequelae of COVID-19 at 2 years. Nature Medicine. 2023 Sep 1;29(9):2347–57.

23. Gallant C, Kenny P. Oral glucocorticoids and their complications. A review. Journal of the American Academy of Dermatology. 1986/02/01 ed. 1986 Feb;14(2 Pt 1):161–77.

24. Richards RN. Side effects of short-term oral corticosteroids. Journal of cutaneous medicine and surgery. 2008/03/19 ed. 2008 Mar;12(2):77–81.

25. Waljee AK, Rogers MAM, Lin P, Singal AG, Stein JD, Marks RM, et al. Short term use of oral corticosteroids and related harms among adults in the United States: population based cohort study. BMJ (Online). 2017;357:j1415–j1415.

26. Yao TC, Huang YW, Chang SM, Tsai SY, Wu AC, Tsai HJ. Association Between Oral Corticosteroid Bursts and Severe Adverse Events : A Nationwide Population-Based Cohort Study. Ann Intern Med. 2020;173(5):325–30.

27. Briot K, Roux C. Glucocorticoid-induced osteoporosis. (2056-5933 (Print)).

28. Haagsma JA, Graetz N, Bolliger I, Naghavi M, Higashi H, Mullany EC, et al. The global burden of injury: incidence, mortality, disability-adjusted life years and time trends from the Global Burden of Disease study 2013. Injury prevention : journal of the International Society for Child and Adolescent Injury Prevention. 2015/12/05 ed. 2016 Feb;22(1):3–18.

29. Rajan SS, Sawe HR, Iyullu AJ, Kaale DA, Olambo NA, Mfinanga JA, et al. Profile and outcome of patients with upper gastrointestinal bleeding presenting to urban emergency departments of tertiary hospitals in Tanzania. BMC Gastroenterol. 2019/12/12 ed. 2019 Dec 10;19(1):212.

30. Aljarad Z, Mobayed BB. The mortality rate among patients with acute upper GI bleeding (with/without EGD) at Aleppo University Hospital: A retrospective study. Annals of Medicine and Surgery. 2021 Nov 1;71:102958.

31. Amin SK, Antunes C. Lower Gastrointestinal Bleeding. In: StatPearls [Internet]. Treasure Island (FL): StatPearls Publishing Copyright © 2023, StatPearls Publishing LLC.; 2023 [cited 2023 Jul 5]. Available from: Guidelines for treatment of drug-susceptible tuberculosis and patient care, 2017 update

32. Beckman MG, Hooper WC, Critchley SE, Ortel TL. Venous thromboembolism: a public health concern. American journal of preventive medicine. 2010/04/02 ed. 2010 Apr;38(4 Suppl):S495-501.

33. Rudd KE, Johnson SC, Agesa KM, Shackelford KA, Tsoi D, Kievlan DR, et al. Global, regional, and national sepsis incidence and mortality, 1990–2017: analysis for the Global Burden of Disease Study. The Lancet. 2020 Jan 18;395(10219):200–11.

34. Prescott HC, Angus DC. Enhancing Recovery From Sepsis: A Review. JAMA. 2018 Jan 2;319(1):62–75.

35. Prescott HC, Osterholzer JJ, Langa KM, Angus DC, Iwashyna TJ. Late mortality after sepsis: propensity matched cohort study. BMJ. 2016 May 17;353:i2375.

36. Taylor CJ, Ordóñez-Mena JM, Roalfe AK, Lay-Flurrie S, Jones NR, Marshall T, et al. Trends in survival after a diagnosis of heart failure in the United Kingdom 2000-2017: population based cohort study. BMJ. 2019 Feb 13;364:l223.

37. Lee JM, Kim ES, Chun HJ, Hwang YJ, Lee JH, Kang SH, et al. Discharge hemoglobin and outcome in patients with acute nonvariceal upper gastrointestinal bleeding. Endosc Int Open. 2016 Aug;4(8):E865-869.

38. Farmakis IT, Barco S, Mavromanoli AC, Agnelli G, Cohen AT, Giannakoulas G, et al. Cost-of-Illness Analysis of Long-Term Health Care Resource Use and Disease Burden in Patients With Pulmonary Embolism: Insights From the PREFER in VTE Registry. J Am Heart Assoc. 2022 Oct 18;11(20):e027514.

39. Salomon JAP, Vos TP, Hogan DRP, Gagnon MMS, Naghavi MP, Mokdad AP, et al. Common values in assessing health outcomes from disease and injury: disability weights measurement study for the Global Burden of Disease Study 2010. Lancet, The. 2012;380(9859):2129–43.

40. Yan T, Zhu S, Yin X, Xie C, Xue J, Zhu M, et al. Burden, Trends, and Inequalities of Heart Failure Globally, 1990 to 2019: A Secondary Analysis Based on the Global Burden of Disease 2019 Study. Journal of the American Heart Association. 2023 Mar 21;12(6):e027852.

41. Alter DA, Ko DT, Tu JV, Stukel TA, Lee DS, Laupacis A, et al. The average lifespan of patients discharged from hospital with heart failure. J Gen Intern Med. 2012 Sep;27(9):1171–9.

42. Schiff MH, Kremer JM, Jahreis A, Vernon E, Isaacs JD, van Vollenhoven RF. Integrated safety in tocilizumab clinical trials. Arthritis Res Ther. 2011 Sep 1;13(5):R141.

43. Cantini F, Nannini C, Niccoli L, Petrone L, Ippolito G, Goletti D. Risk of Tuberculosis Reactivation in Patients with Rheumatoid Arthritis, Ankylosing Spondylitis, and Psoriatic Arthritis Receiving Non-Anti-TNF-Targeted Biologics. Mediators of inflammation. 2017/07/01 ed. 2017;2017:8909834.

44. Tiemersma EW, van der Werf MJ, Borgdorff MW, Williams BG, Nagelkerke NJD. Natural history of tuberculosis: duration and fatality of untreated pulmonary tuberculosis in HIV negative patients: a systematic review. PLoS One. 2011 Apr 4;6(4):e17601.

45. Center for Disease Control. Treatment for TB Disease [Internet]. 2023 [cited 2023 Jul 13]. Available from: https://www.cdc.gov/tb/topic/treatment/tbdisease.htm

46. Guidelines for treatment of drug-susceptible tuberculosis and patient care, 2017 update [Internet]. Geneva: World Health Organization; 2017. Available from: https://apps.who.int/iris/bitstream/handle/10665/255052/9789241550000-eng.pdf

47. Atif M, Sulaiman SAS, Shafie AA, Babar ZU. Duration of treatment in pulmonary tuberculosis: are international guidelines on the management of tuberculosis missing something? Public Health. 2015 Jun;129(6):777–82.

48. Aljayyoussi G, Jenkins VA, Sharma R, Ardrey A, Donnellan S, Ward SA, et al. Pharmacokinetic-Pharmacodynamic modelling of intracellular Mycobacterium tuberculosis growth and kill rates is predictive of clinical treatment duration. Scientific Reports. 2017 Mar 29;7(1):502.

49. Lestari BW, McAllister S, Hadisoemarto PF, Afifah N, Jani ID, Murray M, et al. Patient pathways and delays to diagnosis and treatment of tuberculosis in an urban setting in Indonesia. The Lancet Regional Health – Western Pacific [Internet]. 2020 Dec 1 [cited 2023 Jul 10];5. Available from: https://doi.org/10.1016/j.lanwpc.2020.100059

50. Peri AM, Bernasconi DP, Galizzi N, Matteelli A, Codecasa L, Giorgio V, et al. Determinants of patient and health care services delays for tuberculosis diagnosis in Italy: a cross-sectional observational study. BMC Infectious Diseases. 2018 Dec 20;18(1):690.

51. Diallo A, Combary A, Veronese V, Dahourou DL, Ouédraogo S, Traoré IT, et al. Delays in TB Diagnosis and Treatment Initiation in Burkina Faso during the COVID-19 Pandemic. Tropical Medicine and Infectious Disease. 2022;7(9).

52. Alene M, Assemie MA, Yismaw L, Gedif G, Ketema DB, Gietaneh W, et al. Patient delay in the diagnosis of tuberculosis in Ethiopia: a systematic review and meta-analysis. BMC Infectious Diseases. 2020 Oct 27;20(1):797.

53. Global Tuberculosis Report 2022 [Internet]. Geneva: World Health Organization; 2022 [cited 2023 Jul 14]. Available from: https://www.who.int/teams/global-tuberculosis-programme/tb-reports/global-tuberculosis-report-2022

54. Abate KH, Abu-Raddad LJ, Adetokunboh O, Afshin A, Agrawal S, Alkaabi JM, et al. Global, regional, and national disability-adjusted life-years (DALYs) for 333 diseases and injuries and healthy life expectancy (HALE) for 195 countries and territories, 1990–2016: a systematic analysis for the Global Burden of Disease Study 2016. The Lancet (British edition). 2017;390(10100):1260–344.

55. Torres-Rueda S, Sweeney S, Bozzani F, Naylor NR, Baker T, Pearson C, et al. Stark choices: exploring health sector costs of policy responses to COVID-19 in low-income and middle-income countries. BMJ Glob Health. 2021;6(12):e005759.

56. Wang J, Levi J, Ellis L, Hill A. Minimum Manufacturing Costs, National Prices, and Estimated Global Availability of New Repurposed Therapies for Coronavirus Disease 2019. Open Forum Infectious Diseases. 2022;9(1):ofab581.

57. Hill Andrew M, Barber Melissa J, Gotham Dzintars. Estimated costs of production and potential prices for the WHO Essential Medicines List. BMJ Global Health. 2018 Jan 1;3(1):e000571.

58. Shrestha P, Cooper BS, Coast J, Oppong R, Do Thi Thuy N, Phodha T, et al. Enumerating the economic cost of antimicrobial resistance per antibiotic consumed to inform the evaluation of interventions affecting their use. Antimicrobial resistance and infection control. 2018/08/18 ed. 2018;7:98.

59. Gautham M, Miller R, Rego S, Goodman C. Availability, Prices and Affordability of Antibiotics Stocked by Informal Providers in Rural India: A Cross-Sectional Survey. Antibiotics (Basel, Switzerland). 2022/04/24 ed. 2022 Apr 14;11(4).

60. Siapka M, Vassall A, Cunnama L, Pineda C, Cerecero D, Sweeney S, et al. Cost of tuberculosis treatment in low- and middle-income countries: systematic review and meta-regression. The international journal of tuberculosis and lung disease : the official journal of the International Union against Tuberculosis and Lung Disease. 2020/09/12 ed. 2020 Aug 1;24(8):802–10.

61. Ochalek J, Lomas J, Claxton K. Estimating health opportunity costs in low-income and middle-income countries: a novel approach and evidence from cross-country data. BMJ Glob Health. 2018/11/30 ed. 2018;3(6):e000964.

62. World Health Organization. WHO guide for standardization of economic evaluations of immunization programmes. Second edition. [Internet]. Geneva; 2019 Oct [cited 2023 Jul 27]. Available from: https://apps.who.int/iris/bitstream/handle/10665/329389/WHO-IVB-19.10-eng.pdf

63. The World Bank. World Bank Open Data [Internet]. The World Bank; 2023 [cited 2023 Jul 15]. Available from: https://data.worldbank.org

64. Torres-Rueda S, Sweeney S, Bozzani F, Naylor NR, Baker T, Pearson C, et al. Stark choices: exploring health sector costs of policy responses to COVID-19 in low-income and middle-income countries. BMJ Glob Health. 2021;6(12):e005759.

65. Serje J, Bertram MY, Brindley C, Lauer JA. Global health worker salary estimates: an econometric analysis of global earnings data. Cost Eff Resour Alloc. 2018/03/16 ed. 2018;16:10.

66. Manjate NJ, Sitoe N, Sambo J, Guimarães E, Canana N, Chilaúle J, et al. Testing for SARS-CoV-2 in resource-limited settings: A cost analysis study of diagnostic tests using different Ag-RDTs and RT-PCR technologies in Mozambique. PLOS Glob Public Health. 2023;3(6):e0001999.

67. Cedro VQM, de Lima Gomes S, Simões ACCD, Sverzut T do VL, Bertti KCX, Tristão MT, et al. Cost-effectiveness analysis of COVID-19 tests in the unified health system. Cost Eff Resour Alloc. 2023 Sep 13;21(1):64.

68. Ismaila H, Asamani JA, Lokossou VK, Oduro-Mensah E, Nabyonga-Orem J, Akoriyea SK. The cost of clinical management of SARS-COV-2 (COVID-19) infection by level of disease severity in Ghana: a protocol-based cost of illness analysis. BMC Health Services Research. 2021 Oct 18;21(1):1115.

69. Yigezu A, Zewdie SA, Mirkuzie AH, Abera A, Hailu A, Agachew M, et al. Cost-analysis of COVID-19 sample collection, diagnosis, and contact tracing in low resource setting: The case of Addis Ababa, Ethiopia. PLOS ONE. 2022;17(6):e0269458.

70. Minhas N, Gurav YK, Sambhare S, Potdar V, Choudhary ML, Bhardwaj SD, et al. Cost-analysis of real time RT-PCR test performed for COVID-19 diagnosis at India’s national reference laboratory during the early stages of pandemic mitigation. PLoS One. 2023;18(1):e0277867.

71. The Global Fund. Pooled Procurement Mechanism Reference Pricing: COVID-19 diagnostics [Internet]. [cited 2024 Mar 15]. Available from: https://www.theglobalfund.org/media/10233/covid19_diagnosticsreferenceprices_table_en.pdf

72. Thompson A. You can finally buy Covid-19 home tests in South Africa - here’s what you need to know. 2022 Nov 23 [cited 2023 Jul 13]; Available from: https://www.news24.com/news24/bi-archive/you-can-now-buy-at-home-covid-19-tests-in-south-africa-for-r50-a-test-2022-11

73. The Guardian. How much does a Covid test cost around the world? 2022 Nov 2 [cited 2023 Jul 13]; Available from: https://www.theguardian.com/world/2022/feb/11/how-much-does-a-covid-test-cost-around-the-world

74. UNICEF. Boosting the availability of affordable, quality-assured COVID-19 tests [Internet]. 2022 [cited 2023 Oct 25]. Available from: https://www.unicef.org/supply/stories/boosting-availability-affordable-quality-assured-covid-19-tests

75. The Global Fund. Pooled Procurement Mechanism Reference Pricing: COVID-19 diagnostics [Internet]. [cited 2024 Mar 15]. Available from: https://www.theglobalfund.org/media/10233/covid19_diagnosticsreferenceprices_table_en.pdf

76. Bogere N, Bongomin F, Katende A, Ssebambulidde K, Ssengooba W, Ssenfuka H, et al. Performance and cost-effectiveness of a pooled testing strategy for SARS-CoV-2 using real-time polymerase chain reaction in Uganda. Int J Infect Dis. 2021 Dec;113:355–8.

77. Hurtado AV, Nguyen HT, Schenkel V, Wachinger J, Seybold J, Denkinger CM, et al. The economic cost of implementing antigen-based rapid diagnostic tests for COVID-19 screening in high-risk transmission settings: evidence from Germany. Health Economics Review. 2022 Feb 14;12(1):15.

78. Van Staa TP, Leufkens HGM, Abenhaim L, Zhang B, Cooper C. Use of Oral Corticosteroids and Risk of Fractures. Journal of Bone and Mineral Research. 2000 Jun 1;15(6):993–1000.

79. Mustafa Diab M, Shearer DW, Kahn JG, Wu HH, Lau B, Morshed S, et al. The Cost of Intramedullary Nailing Versus Skeletal Traction for Treatment of Femoral Shaft Fractures in Malawi: A Prospective Economic Analysis. World journal of surgery. 2018/08/11 ed. 2019 Jan;43(1):87–95.

80. Chokotho L, Donnelley CA, Young S, Lau BC, Wu HH, Mkandawire N, et al. Cost utility analysis of intramedullary nailing and skeletal traction treatment for patients with femoral shaft fractures in Malawi. Acta orthopaedica. 2021;92(4):436–42.

81. Werner K, Lin TK, Risko N, Osiro M, Kalanzi J, Wallis L. The costs of delivering emergency care at regional referral hospitals in Uganda: a micro-costing study. BMC health services research. 2021/03/18 ed. 2021 Mar 16;21(1):232.

82. Nwanna–Nzewunwa O, Agwang E, Carvalho M, Ajiko MM, Oke R, Yoon C, et al. A cost-effectiveness analysis of surgical care delivery in Eastern Uganda-a societal perspective. BMC Health Services Research. 2023 Mar 15;23(1):256.

83. Nwanna–Nzewunwa O, Oke R, Agwang E, Ajiko MM, Yoon C, Carvalho M, et al. The societal cost and economic impact of surgical care on patients’ households in rural Uganda; a mixed method study. BMC Health Services Research. 2021 Jun 9;21(1):568.

84. Rajabi M, Ostovar A, Sari AA, Sajjadi-Jazi SM, Fahimfar N, Larijani B, et al. Direct costs of common osteoporotic fractures (Hip, Vertebral and Forearm) in Iran. BMC Musculoskeletal Disorders. 2021 Jul 31;22(1):651.

85. Ostovar A, Mousavi A, Sajjadi-Jazi SM, Rajabi M, Larijani B, Fahimfar N, et al. The economic burden of osteoporosis in Iran in 2020. Osteoporosis International. 2022 Nov 1;33(11):2337–46.

86. Nunes AM, Canhão H, Rodrigues TL. Enabling Cape Verde to Perform Total Hip Replacement: Cost-Benefit Study. Acta medica portuguesa. 2019/01/27 ed. 2018 Dec 28;31(12):738–41.

87. Gosselin RA, Heitto M, Zirkle L. Cost-effectiveness of replacing skeletal traction by interlocked intramedullary nailing for femoral shaft fractures in a provincial trauma hospital in Cambodia. International orthopaedics. 2009/05/14 ed. 2009 Oct;33(5):1445–8.

88. DM Kamau, LN Gakuu, EM Gakuya, Sang E. Comparison of closed femur fracture: Skeletal traction and intramedullary nailing cost-effectiveness. East African Orthopaedic Journal. 2014;8(1).

89. Famurewa BA, Aregbesola SB, Alade OT, Akinniyi TA. Treatment costs of mandibular fractures in a Nigerian hospital. Oral and Maxillofacial Surgery. 2022 Sep 1;26(3):417–22.

90. Akhiwu BI, Suleiman HH, Muktar M, Amole IO. Cost of illness in patients with mandibular fracture following road traffic crash and its socioeconomic implications in KANO STATE, NIGERIA. (2276-6944 (Print)).

91. Umo I, James K, Didilemu F, Sinen B, Borchem I, Inaido D, et al. The direct medical cost of trauma aetiologies and injuries in a resource limited setting of Papua New Guinea: A prospective cost of illness study. The Lancet Regional Health – Western Pacific [Internet]. 2022 [cited 2023 Jun 1];20. Available from: https://doi.org/10.1016/j.lanwpc.2021.100379

92. Diémé CB. Economic cost of the treatment of fractures among old people: a preliminary study in dakar teaching hospital. Geriatric orthopaedic surgery & rehabilitation. 2014/11/02 ed. 2014 Sep;5(3):127–30.

93. Roberts HJ, Donnelley CA, Haonga BT, Kramer E, Eliezer EN, Morshed S, et al. Intramedullary nailing versus external fixation for open tibia fractures in Tanzania: a cost analysis. OTA international : the open access journal of orthopaedic trauma. 2021/11/09 ed. 2021 Sep;4(3):e146.

94. Haonga BT, Areu MMM, Challa ST, Liu MB, Elieza E, Morshed S, et al. Early treatment of open diaphyseal tibia fracture with intramedullary nail versus external fixator in Tanzania: Cost effectiveness analysis using preliminary data from Muhimbili Orthopaedic Institute. Sicot-j. 2019/06/18 ed. 2019;5:20.

95. Kramer EJ, Shearer DW, Marseille E, Haonga B, Ngahyoma J, Eliezer E, et al. The Cost of Intramedullary Nailing for Femoral Shaft Fractures in Dar es Salaam, Tanzania. World journal of surgery. 2016/03/18 ed. 2016 Sep;40(9):2098–108.

96. Melione LP, Mello-Jorge MH. [Unified National Health System costs in São José dos Campos, São Paulo State, Brazil, for hospital admissions due to external causes]. Cadernos de saude publica. 2008/08/19 ed. 2008 Aug;24(8):1814–24.

97. Carlos F, Clark P, Galindo-Suárez RM, Chico-Barba LG. Health care costs of osteopenia, osteoporosis, and fragility fractures in Mexico. Archives of Osteoporosis. 2013 Mar 23;8(1):125.

98. Oliveira CC, Borba VZC. Epdemiology of femur fractures in the elderly and cost to the State of Paraná, Brazil. Acta ortopedica brasileira. 2017/09/29 ed. 2017 Jul;25(4):155–8.

99. Loures FB, Chaoubah A, Maciel VS, Paiva EP, Salgado PP, Netto Á C. Cost-effectiveness of surgical treatment for hip fractures among the elderly in Brazil. Revista brasileira de ortopedia. 2015/08/01 ed. 2015 Jan;50(1):38–42.

100. Yilin Wang, Haoran Cui, Dianying Zhang, Peixun Zhang. Hospitalisation cost analysis on hip fracture in China: a multicentre study among 73 tertiary hospitals. BMJ Open. 2018 Apr 1;8(4):e019147.

101. Qu B, Ma Y, Yan M, Wu HH, Fan L, Liao DF, et al. The economic burden of fracture patients with osteoporosis in western China. Osteoporosis international : a journal established as result of cooperation between the European Foundation for Osteoporosis and the National Osteoporosis Foundation of the USA. 2014/04/03 ed. 2014 Jul;25(7):1853–60.

102. Wu J, Qu Y, Wang K, Chen Y. Healthcare Resource Utilization and Direct Medical Costs for Patients With Osteoporotic Fractures in China. Value in Health Regional Issues. 2019 May 1;18:106–11.

103. Hou X l, Liu J y, Fan X h, Zhang N, Cao G l, Guo Z b, et al. Secular trends of incidence and hospitalization cost of hip fracture in Tangshan, China. Osteoporosis International. 2022 Jan 1;33(1):89–96.

104. Quevedo-Tejero Edel C, Zavala-González MA, Hernández-Gamas Adel C, Hernández-Ortega HM. [Hip fracture in older adults: prevalence and costs in two hospitals. Tabasco, Mexico, 2009]. Revista peruana de medicina experimental y salud publica. 2011/11/17 ed. 2011 Jul;28(3):440–5.

105. Pech-Ciau BA, Lima-Martínez EA, Espinosa-Cruz GA, Pacho-Aguilar CR, Huchim-Lara O, Alejos-Gómez RA. [Hip fracture in the elderly: epidemiology and costs of care]. Acta ortopedica mexicana. 2022/02/10 ed. 2021 Jul;35(4):341–7.

106. Mafirakureva N, Paruk F, Cassim B, Lukhele M, Gregson CL, Noble SM. The healthcare system costs of hip fracture care in South Africa. Osteoporosis international : a journal established as result of cooperation between the European Foundation for Osteoporosis and the National Osteoporosis Foundation of the USA. 2023/01/28 ed. 2023 Apr;34(4):803–13.

107. Woratanarat P, Wajanavisit W, Lertbusayanukul C, Loahacharoensombat W, Ongphiphatanakul B. Cost analysis of osteoporotic hip fractures. Journal of the Medical Association of Thailand = Chotmaihet thangphaet. 2006/07/28 ed. 2005 Oct;88 Suppl 5:S96-104.

108. Tanriover MD, Oz SG, Tanriover A, Kilicarslan A, Turkmen E, Guven GS, et al. Hip fractures in a developing country: osteoporosis frequency, predisposing factors and treatment costs. Archives of gerontology and geriatrics. 2009/06/02 ed. 2010 May;50(3):e13-8.

109. Velasco-Murillo V, Navarrete-Hernández E, Pozos-Cavanzo JL, Ojeda-Mijares RI, Camacho-Rodríguez Mde L. [Fractures in postmenopausal women in the IMSS: frequency and costs of hospital care]. Gaceta medica de Mexico. 2003/11/26 ed. 2003 Sep;139(5):453–8.

110. Köksal A, Çimen O, Öner A, Aycan OE, Abul K, Akgün H, et al. Intramedullary nailing versus minimally invasive plating in the treatment of distal tibial extra-articular fractures: Comparison of cost analysis in Turkey. Turkish Journal of Trauma and Emergency Surgery. 2021;27(5):558–64.

111. Sangchan A, Sawadpanitch K, Mairiang P, Chunlertrith K, Sukeepaisarnjaroen W, Sutra S, et al. Hospitalized incidence and outcomes of upper gastrointestinal bleeding in Thailand. J Med Assoc Thai. 2012 Jul;95 Suppl 7:S190-195.

112. Atipo-Ibara BI, Ondele-Ngoli A, Deby G, Ibara JR, Okouo M, Ngoma-Kadoulou P, et al. [Cost of of hospital management of cirrhoses and its complications at the University Hospital of Brazzaville]. Medecine tropicale : revue du Corps de sante colonial. 2004/07/01 ed. 2004;64(1):50–2.

113. Chait MM. Lower GI Bleeding in Patients With Cirrhosis: 3034. Official journal of the American College of Gastroenterology | ACG [Internet]. 2018;113. Available from: https://journals.lww.com/ajg/fulltext/2018/10001/lower_gi_bleeding_in_patients_with_cirrhosis__3034.3033.aspx

114. Pok LSL, Shabaruddin FH, Dahlui M, Sockalingam S, Mohamed Said MS, Rosman A, et al. Clinical and economic implications of upper gastrointestinal adverse events in Asian rheumatological patients on long-term non-steroidal anti-inflammatory drugs. International journal of rheumatic diseases. 2018/01/10 ed. 2018 May;21(5):943–51.

115. Chang SS, Wu JH, Liu Y, Zhang T, Du X, Dong JZ, et al. In-hospital direct costs for thromboembolism and bleeding in Chinese patients with atrial fibrillation. Chronic Diseases and Translational Medicine. 2018 Jun 1;4(2):127–34.

116. Campbell HE, Stokes EA, Bargo D, Logan RF, Mora A, Hodge R, et al. Costs and quality of life associated with acute upper gastrointestinal bleeding in the UK: cohort analysis of patients in a cluster randomised trial. BMJ Open. 2015;5(4):e007230.

117. Cryer BL, Wilcox CM, Henk HJ, Zlateva G, Chen L, Zarotsky V. The economics of upper gastrointestinal bleeding in a US managed-care setting: a retrospective, claims-based analysis. Journal of Medical Economics. 2010 Mar 1;13(1):70–7.

118. Whelan CT, Chen C, Kaboli P, Siddique J, Prochaska M, Meltzer DO. Upper versus lower gastrointestinal bleeding: a direct comparison of clinical presentation, outcomes, and resource utilization. J Hosp Med. 2010 Mar;5(3):141–7.

119. Bai G, Zare H. Hospital Cost Structure and the Implications on Cost Management During COVID-19. J Gen Intern Med. 2020 Sep;35(9):2807–9.

120. Yang L, Wu J. Cost-effectiveness of rivaroxaban compared with enoxaparin plus warfarin for the treatment of hospitalised acute deep vein thrombosis in China. BMJ open. 2020/08/02 ed. 2020 Jul 30;10(7):e038433.

121. Sargin M, Erdogan SB, Bastopcu M, Arslanhan G, Tasdemir MM, Orhan G. Cost of Healthcare Associated With Deep Vein Thrombosis in Patients Treated With Warfarin in Turkey: 2010-2013 Database Analysis of a Tertiary Care Center. Value in Health Regional Issues. 2019;19:81–6.

122. Niyomsri S, Nimworapan M, Wongcharoen W, Dilokthornsakul P. Economic Evaluation of Direct Oral Anticoagulants Compared to Warfarin for Venous Thromboembolism in Thailand: A Cost-Utility Analysis. International journal of environmental research and public health. 2023/02/26 ed. 2023 Feb 11;20(4).

123. Derseh MT, Solomon K, Tamene W, Beneberu W, Yayehrad AT, Ambaye AS. A Cost Effectiveness Analysis of Rivaroxaban Compared to Warfarin for Deep Vein Thrombosis (DVT) Treatment in Ethiopia. ClinicoEconomics and outcomes research : CEOR. 2021/09/25 ed. 2021;13:821–34.

124. Salman O, Procter SR, McGregor C, Paul P, Hutubessy R, Lawn JE, et al. Systematic Review on the Acute Cost-of-illness of Sepsis and Meningitis in Neonates and Infants. The Pediatric Infectious Disease Journal [Internet]. 2020;39(1). Available from: https://journals.lww.com/pidj/Fulltext/2020/01000/Systematic_Review_on_the_Acute_Cost_of_illness_of.7.aspx

125. Aerts C, Leahy S, Mucasse H, Lala S, Bramugy J, Tann CJ, et al. Quantifying the Acute Care Costs of Neonatal Bacterial Sepsis and Meningitis in Mozambique and South Africa. Clinical infectious diseases : an official publication of the Infectious Diseases Society of America. 2021/11/03 ed. 2022 Jan 20;74(Suppl_1):S64-s69.

126. Bui MH, Khuong QL, Le PA, Nguyen TA, Doan QH, Duong TD, et al. Cost of postoperative sepsis in Vietnam. Scientific Reports. 2022 Mar 22;12(1):4876.

127. Quintano Neira RA, Hamacher S, Japiassú AM. Epidemiology of sepsis in Brazil: Incidence, lethality, costs, and other indicators for Brazilian Unified Health System hospitalizations from 2006 to 2015. PLOS ONE. 2018;13(4):e0195873.

128. Alicja Jasinska-Piadlo, Patricia Campbell. Management of patients with heart failure and preserved ejection fraction. Heart. 2023 Jun 1;109(11):874.

129. Rezapour A, Azari S, Arabloo J, Kolivand P, Behzadifar M, Omidi N, et al. Cost-Effectiveness of Sacubitril/Valsartan Compared with Enalapril in Patients with Heart Failure with Reduced Ejection Fraction: A Systematic Review. (1735-5370 (Print)).

130. Krittayaphong R, Permsuwan U. Cost-Utility Analysis of Combination Empagliflozin and Standard Treatment Versus Standard Treatment Alone in Thai Heart Failure Patients with Reduced or Preserved Ejection Fraction. American Journal of Cardiovascular Drugs. 2022 Sep 1;22(5):577–90.

131. Ogah OS, Stewart S, Onwujekwe OE, Falase AO, Adebayo SO, Olunuga T, et al. Economic burden of heart failure: investigating outpatient and inpatient costs in Abeokuta, Southwest Nigeria. (1932-6203 (Electronic)).

132. Rezapour A, Tashakori-Miyanroudi M, Haghjoo M, Barzegar M, Tatarpour P, Souresrafil AAO, et al. Cost Effectiveness of Adding Dapagliflozin to Standard Care in Heart Failure Patients with Reduced Ejection Fraction: A Systematic Review. (1179-187X (Electronic)).

133. Krittayaphong R, Permsuwan U. Cost-utility analysis of add-on dapagliflozin treatment in heart failure with reduced ejection fraction. Int J Cardiol [Internet]. 2021;322. Available from: https://doi.org/10.1016/j.ijcard.2020.08.017

134. Abdelhamid M, Elsisi GH, Seyam A. Dapagliflozin cost-effectiveness analysis in heart failure patients in Egypt. J Med Econ [Internet]. 2022;25. Available from: https://doi.org/10.1080/13696998.2022.2054226

135. Gil-Rojas Y, Lasalvia P, Garcia A. Cost-utility of dapagliflozin plus standard treatment compared to standard treatment for the management of heart failure with reduced ejection fraction in Colombia. Expert Rev Pharmacoecon Outcomes Res [Internet]. 2022;22. Available from: https://doi.org/10.1080/14737167.2022.1997595

136. Jiang Y, Zheng R, Sang H. Cost-effectiveness of adding SGLT2 inhibitors to standard treatment for heart failure with reduced ejection fraction patients in China. Front Pharmacol [Internet]. 2021;12. Available from: https://doi.org/10.3389/fphar.2021.733681

137. Yao Y, Zhang R, An T. Cost-effectiveness of adding dapagliflozin to standard treatment for heart failure with reduced ejection fraction patients in China. ESC Heart Fail [Internet]. 2020;7. Available from: https://doi.org/10.1002/ehf2.12844

138. Lesyuk W, Kriza C, Kolominsky-Rabas P. Cost-of-illness studies in heart failure: a systematic review 2004–2016. BMC Cardiovascular Disorders. 2018 May 2;18(1):74.

139. Karaye KM, Dokainish H, ElSayed A, Mondo C, Damasceno A, Sliwa K, et al. Clinical Profiles and Outcomes of Heart Failure in Five African Countries: Results from INTER-CHF Study. Glob Heart. 2021;16(1):50.

140. Armstrong-Hough M, Sharma S, Kishore SP, Akiteng AR, Schwartz JI. Variation in the availability and cost of essential medicines for non-communicable diseases in Uganda: A descriptive time series analysis. PloS one. 2020/12/29 ed. 2020;15(12):e0241555.

141. World Health Organization. Guidelines for the clinical management of severe illness from influenza virus infections [Internet]. Geneva; [cited 2023 Jul 27]. Available from: https://apps.who.int/iris/bitstream/handle/10665/352453/9789240040816-eng.pdf?sequence=1&isAllowed=y

142. National Institute for Health and Care Excellence. Pneumocystis pneumonia, treatment summaries [Internet]. 2023 [cited 2023 Jul 31]. Available from: https://bnf.nice.org.uk/treatment-summaries/pneumocystis-pneumonia/

143. National Institute for Health and Care Excellence. Tuberculosis, NICE guideline [Internet]. 2019 [cited 2023 Jul 31]. Available from: https://www.nice.org.uk/guidance/ng33/resources/tuberculosis-pdf-1837390683589

144. National Institute for Communicable Diseases. Typhoid: NICD recommendations for diagnosis, management and public health response [Internet]. 2016 [cited 2023 Jul 31]. Available from: https://www.nicd.ac.za/assets/files/Guidelines_typhoid_20160125.pdf

145. National Institute for Health and Care Excellence. Croup [Internet]. 2022 May [cited 2023 Jul 31]. Available from: https://cks.nice.org.uk/topics/croup/

146. World Health Organization. Guidelines for the treatment of malaria, Third edition [Internet]. 2015 [cited 2023 Jul 31]. (Global, regional, and national disability-adjusted life-years (DALYs) for 359 diseases and injuries and healthy life expectancy). Available from: https://apps.who.int/iris/bitstream/handle/10665/162441/9789241549127_eng.pdf

147. Principi N, Bianchini S, Baggi E, Esposito S. No evidence for the effectiveness of systemic corticosteroids in acute pharyngitis, community-acquired pneumonia and acute otitis media. European Journal of Clinical Microbiology & Infectious Diseases. 2013 Feb 1;32(2):151–60.

148. National Institute for Health and Care Excellence. Pneumonia in adults: diagnosis and management, clinical guideline [Internet]. 2022. Available from: https://www.nice.org.uk/guidance/cg191/resources/pneumonia-in-adults-diagnosis-and-management-pdf-35109868127173

149. Lee FEH, Walsh EE, Falsey AR. The effect of steroid use in hospitalized adults with respiratory syncytial virus-related illness. Chest. 2011 Nov;140(5):1155–61.

150. Bandara SMR, Herath HMMTB. Effectiveness of corticosteroid in the treatment of dengue - A systemic review. Heliyon. 2018 Sep;4(9):e00816.

151. Jartti T, Nieminen R, Vuorinen T, Lehtinen P, Vahlberg T, Gern J, et al. Short- and long-term efficacy of prednisolone for first acute rhinovirus-induced wheezing episode. J Allergy Clin Immunol. 2015 Mar;135(3):691-698.e9.

152. Hayward G, Thompson MJ, Perera R, Del Mar CB, Glasziou PP, Heneghan CJ. Corticosteroids for the common cold. The Cochrane database of systematic reviews. 2015/10/16 ed. 2015 Oct 13;2015(10):Cd008116.

153. Orimadegun A, Ogunbosi B, Orimadegun B. Hypoxemia predicts death from severe falciparum malaria among children under 5 years of age in Nigeria: the need for pulse oximetry in case management. Afr Health Sci. 2014 Jun;14(2):397–407.

154. Cabrales P, Martins YC, Ong PK, Zanini GM, Frangos JA, Carvalho LJ. Cerebral tissue oxygenation impairment during experimental cerebral malaria. Virulence. 2013 Nov 15;4(8):686–97.

155. Wang DH, Jia HM, Zheng X, Xi XM, Zheng Y, Li WX. Attributable mortality of ARDS among critically ill patients with sepsis: a multicenter, retrospective cohort study. BMC Pulmonary Medicine. 2024 Mar 4;24(1):110.

156. Lansbury L, Rodrigo C, Leonardi-Bee J, Nguyen-Van-Tam J, Lim WS. Corticosteroids as adjunctive therapy in the treatment of influenza. The Cochrane database of systematic reviews. 2019/02/25 ed. 2019 Feb 24;2(2):Cd010406.

157. Lansbury LE, Rodrigo C, Leonardi-Bee J, Nguyen-Van-Tam J, Shen Lim W. Corticosteroids as Adjunctive Therapy in the Treatment of Influenza: An Updated Cochrane Systematic Review and Meta-analysis. (1530-0293 (Electronic)).

158. Navarro-Flores A, Fernandez-Chinguel JE, Pacheco-Barrios N, Soriano-Moreno DR, Pacheco-Barrios K. Global morbidity and mortality of central nervous system tuberculosis: a systematic review and meta-analysis. Journal of neurology. 2022/03/16 ed. 2022 Jul;269(7):3482–94.

159. Pasipanodya JG, Mubanga M, Ntsekhe M, Pandie S, Magazi BT, Gumedze F, et al. Tuberculous Pericarditis is Multibacillary and Bacterial Burden Drives High Mortality. EBioMedicine. 2015 Nov;2(11):1634–9.

160. Mayosi BM, Burgess LJ, Doubell AF. Tuberculous Pericarditis. Circulation. 2005 Dec 6;112(23):3608–16.

161. Aberdein J, Singer M. Clinical review: A systematic review of corticosteroid use in infections. Critical Care. 2005 Nov 22;10(1):203.

162. Aregbesola A, Tam C, Kothari A, Le ML, Ragheb M, Klassen T. Glucocorticoids for croup in children. Cochrane Database of Systematic Reviews [Internet]. 2023;(1). Available from: https://doi.org//10.1002/14651858.CD001955.pub5

163. Fernandes RM, Oleszczuk M, Woods CR, Rowe BH, Cates CJ, Hartling L. The Cochrane Library and safety of systemic corticosteroids for acute respiratory conditions in children: an overview of reviews. Evid Based Child Health. 2014 Sep;9(3):733–47.

164. Ding L, Huang H, Wang H, He H. Adjunctive corticosteroids may be associated with better outcome for non-HIV Pneumocystis pneumonia with respiratory failure: a systemic review and meta-analysis of observational studies. (2110-5820 (Print)).

165. Briel M, Boscacci R, Furrer H, Bucher HC. Adjunctive corticosteroids for Pneumocystis jiroveci pneumonia in patients with HIV infection: a meta-analysis of randomised controlled trials. BMC Infectious Diseases. 2005 Nov 7;5(1):101.

166. Rubin R. The Dreaded “Twindemic” of Influenza and COVID-19 Has Not Yet Materialized—Might This Be the Year? JAMA. 2022 Oct 18;328(15):1488–9.

167. Bellizzi S, Panu Napodano CM, Pinto S, Pichierri G. COVID-19 and seasonal influenza: The potential 2021-22 “Twindemic”. Vaccine. 2022 May 26;40(24):3286–7.

168. GBD 2017 DALYs and Hale Collaborators. Global, regional, and national disability-adjusted life-years (DALYs) for 359 diseases and injuries and healthy life expectancy (HALE) for 195 countries and territories, 1990–2017: a systematic analysis for the Global Burden of Disease Study 2017. The Lancet. 2018 Nov 10;392(10159):1859–922.

169. Acevedo-Rodriguez JG, Zamudio C, Kojima N, Krapp F, Tsukayama P, Sal Y Rosas Celi VG, et al. Influenza incidence, lineages, and vaccine effectiveness estimates in Lima, Peru, 2023. Lancet Microbe. 2024 Apr;5(4):e308–9.

170. Gentilotti E, Górska A, Tami A, Gusinow R, Mirandola M, Rodríguez Baño J, et al. Clinical phenotypes and quality of life to define post-COVID-19 syndrome: a cluster analysis of the multinational, prospective ORCHESTRA cohort. EClinicalMedicine. 2023 Aug;62:102107.

171. Özcan S, İnce O, Güner A, Katkat F, Dönmez E, Tuğrul S, et al. Long-Term Clinical Consequences of Patients Hospitalized for COVID-19 Infection. Anatol J Cardiol. 2022 Apr;26(4):305–15.

172. Huang L, Li X, Gu X, Zhang H, Ren L, Guo L, et al. Health outcomes in people 2 years after surviving hospitalisation with COVID-19: a longitudinal cohort study. Lancet Respir Med. 2022 Sep;10(9):863–76.

173. Bek LM, Berentschot JC, Heijenbrok-Kal MH, Huijts S, van Genderen ME, Vlake JH, et al. Symptoms persisting after hospitalisation for COVID-19: 12 months interim results of the CO-FLOW study. ERJ Open Res. 2022 Oct;8(4):00355–2022.

174. Abdelhafiz AS, Ali A, Maaly AM, Mahgoub MA, Ziady HH, Sultan EA. Predictors of post-COVID symptoms in Egyptian patients: Drugs used in COVID-19 treatment are incriminated. PLOS ONE. 2022 Mar 31;17(3):e0266175.

175. Nair CV, Moni M, Edathadathil F, A A, Prasanna P, Pushpa Raghavan R, et al. Incidence and Characterization of Post-COVID-19 Symptoms in Hospitalized COVID-19 Survivors to Recognize Syndemic Connotations in India: Single-Center Prospective Observational Cohort Study. JMIR Form Res. 2023 Apr 18;7:e40028.

176. Alghamdi SA, Alfares MA, Alsulami RA, Alghamdi AF, Almalawi AM, Alghamdi MS, et al. Post-COVID-19 Syndrome: Incidence, Risk Factor, and the Most Common Persisting Symptoms. Cureus. 2022 Nov;14(11):e32058.

177. Davelaar J, Jessurun N, Schaap G, Bode C, Vonkeman H. The effect of corticosteroids, antibiotics, and anticoagulants on the development of post-COVID-19 syndrome in COVID-19 hospitalized patients 6 months after discharge: a retrospective follow up study. Clinical and Experimental Medicine [Internet]. 2023 Aug 8; Available from: https://doi.org/10.1007/s10238-023-01153-7

178. O A Caguana Vélez, M C Cumpli Gargallo, M Comas serrano, M Posso Rivera, X Duran Jordà, E Balcells Vilarnau, et al. PERSISTING SYMPTOMS IN A COHORT OF 1966 SUBJECTS WITH SARS-COV-2 INFECTION: 1 YEAR FOLLOW-UP. Eur Respir J. 2022 Sep 4;60(suppl 66):1341.

179. Mastrorosa I, Del Duca G, Pinnetti C, Lorenzini P, Vergori A, Brita AC, et al. What is the impact of post-COVID-19 syndrome on health-related quality of life and associated factors: a cross-sectional analysis. Health and Quality of Life Outcomes. 2023 Mar 22;21(1):28.

180. Clancy CJ, Nguyen MH. A First Draft of the History of Treating Coronavirus Disease 2019: Use of Repurposed Medications in United States Hospitals. Open Forum Infect Dis. 2021 Feb;8(2):ofaa617.

181. Acosta AM, Mathis AL, Budnitz DS, Geller AI, Chai SJ, Alden NB, et al. COVID-19 Investigational Treatments in Use Among Hospitalized Patients Identified Through the US Coronavirus Disease 2019-Associated Hospitalization Surveillance Network, March 1-June 30, 2020. Open Forum Infect Dis. 2020 Nov;7(11):ofaa528.

182. Kansteiner F. With dexamethasone’s sudden COVID-19 blessing, U.S. steroid supplies plummet. Fierce Pharma [Internet]. 2020 Jun 25 [cited 2023 Oct 16]; Available from: https://www.fiercepharma.com/manufacturing/dexamethasone-s-rise-u-s-steroid-supplies-plummet

183. Närhi F, Moonesinghe SR, Shenkin SD, Drake TM, Mulholland RH, Donegan C, et al. Implementation of corticosteroids in treatment of COVID-19 in the ISARIC WHO Clinical Characterisation Protocol UK: prospective, cohort study. The Lancet Digital Health. 2022 Apr 1;4(4):e220–34.

184. Cutler D. The Economic Cost of Long COVID: An Update [Internet]. Harvard University; 2022 [cited 2023 Oct 20]. Available from: https://scholar.harvard.edu/files/cutler/files/long_covid_update_7-22.pdf

185. Wolff Sagy Y, Feldhamer I, Brammli-Greenberg S, Lavie G. Estimating the economic burden of long-Covid: the additive cost of healthcare utilisation among COVID-19 recoverees in Israel. BMJ Glob Health. 2023 Jul;8(7).

186. Tufts J, Zemedikun DT, Subramanian A, Guan N, Gokhale K, Puja Myles, et al. The cost of primary care consultations associated with long COVID in non-hospitalised adults: a retrospective cohort study using UK primary care data. BMC Prim Care. 2023 Nov 20;24(1):245.

187. United Nations, Department of Economic and Social Affairs, Population Division. World Population Prospects 2019, Online Edition. Rev. 1. 2019.

188. Bekliz Meriem, Adea Kenneth, Puhach Olha, Perez-Rodriguez Francisco, Marques Melancia Stéfane, Baggio Stephanie, et al. Analytical Sensitivity of Eight Different SARS-CoV-2 Antigen-Detecting Rapid Tests for Omicron-BA.1 Variant. Microbiol Spectr. 2022 Aug 8;10(4):e00853-22.

189. World Health Organization. WHO methods and data sources for global burden of disease estimates 2000-2019 [Internet]. 2020 [cited 2023 Nov 12]. Available from: https://cdn.who.int/media/docs/default-source/gho-documents/global-health-estimates/ghe2019_daly-methods.pdf
