# Supplementary material for: Cost-effectiveness of COVID rapid diagnostic tests for patients with severe/critical illness in low- and middle-income countries: A modeling study
Source: PLoS Med. 2024 Jul 18;21(7):e1004429. doi: 10.1371/journal.pmed.1004429 (PMC11293649; doi:10.1371/journal.pmed.1004429)
Supplement: S3 Appendix — (DOCX) [file pmed.1004429.s003.docx]

# **S3 Appendix: Additional figures and tables – most cost-effective options in different scenarios**

This appendix systematically reviews all results in the sensitivity analysis which were not detailed in the main manuscript.

## **A. Impact of treatment side-effects and implications of different influenza prevalence levels**

### **A.1 Most cost-effective option when no or only part of treatment side-effects are accounted for**

S1 Figure shows model outcomes when the only available COVID treatment is corticosteroids, while no side effect of corticosteroids are accounted for: testing is never cost-effective because it is more expensive than treatment (corticosteroids) so giving treatment presumptively to all suspected cases is both cheaper and more effective (health-wise) than testing to guide treatment, at all prevalence levels. At very low prevalence levels, however, as the cost of giving corticosteroids largely to all suspected cases even though very few actually have COVID, presumptive treatment is less cost-effective than treating all suspected patients as non-COVID.

**S1 Figure: Proportion of countries in which a given option is the most cost-effective, if corticosteroid side effects are not accounted for**

**
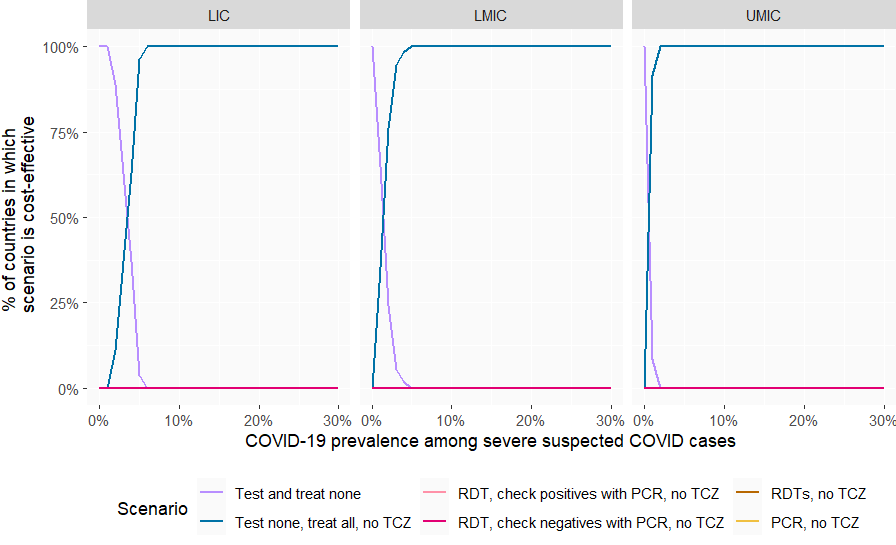
**

** PCR = polymerase chain reaction, RDT = rapid diagnostic tests, TCZ = tocilizumab*

In S2 figure, “generic” side-effects (i.e., those that apply to all patients) are accounted for, but not any disease-specific side-effect. This corresponds to a hypothetical scenario in which influenza prevalence is zero. In this context, testing is cost-effective in upper-middle-income countries for almost any SARS-CoV-2 prevalence value among severe COVID-like patients, but it is almost never cost-effective in low-income countries. Figure 2 however shows that the share of countries in which testing is cost-effective is quite different even at a low 1% influenza prevalence.

**S2 figure: Proportion of countries in which a given option is the most cost-effective, if only generic corticosteroid side effects are accounted for or when influenza prevalence is 0% in severe suspected COVID-like patients**


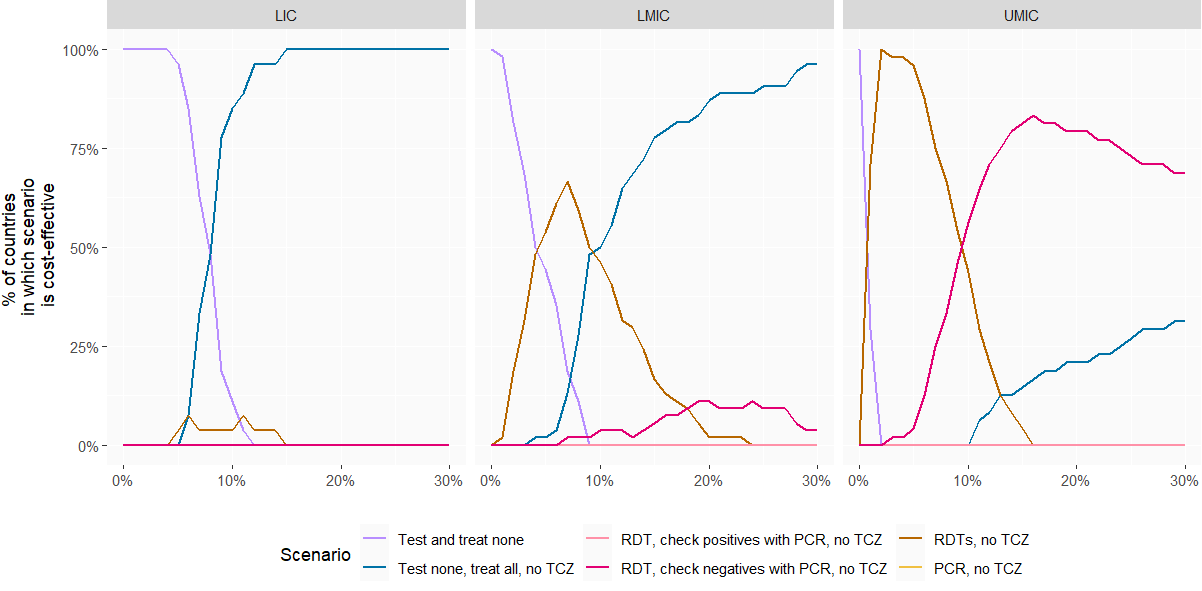


** PCR = polymerase chain reaction, RDT = rapid diagnostic tests, TCZ = tocilizumab*

### **A.2 Net monetary benefits – baseline scenario with all side-effects accounted for and 1% influenza prevalence**

S10 Table shows the average net monetary benefit associated with each testing/treatment option and country income levels, using baseline estimates for all parameters and 1% influenza prevalence (a “low influenza prevalence” scenario). Options with positive net monetary benefits are more cost-effective than the “no testing or treatment” option (reference scenario), even when they are not the most cost-effective option. Hence, RDT testing is (on average) more cost-effective than the reference scenario in LICs at COVID prevalence 8% or above, even though presumptive treatment with corticosteroids is even more cost-effective at high prevalence. In upper-middle-income countries, testing is more cost-effective than doing nothing at any prevalence in the table except when there is no COVID (0% prevalence). In line with intuition, confirming positives with PCR appears more cost-effective on average than confirming negatives at low prevalence rates, while confirming negatives is the most cost-effective option at higher prevalence levels.

**S10 Table: Average net monetary benefit associated with each testing/treatment option across country income ranges (reference: no testing or treatment, influenza prevalence in severe COVID-like, non-COVID patients: 1%)**

| **Income level** | **Testing/treatment scenario** | **COVID-19 prevalence among severe "COVID-like" patients (suspected cases)** | | | | | | | | | | | | | | | |
| --- | --- | --- | --- | --- | --- | --- | --- | --- | --- | --- | --- | --- | --- | --- | --- | --- | --- |
|  |  | 0% | 2% | 4% | 6% | 8% | 10% | 12% | 14% | 16% | 18% | 20% | 22% | 24% | 26% | 28% | 30% |
| All levels | No test, no treatment | 0 | 0 | 0 | 0 | 0 | 0 | 0 | 0 | 0 | 0 | 0 | 0 | 0 | 0 | 0 | 0 |
| LIC | No test, treat presumptively (no TCZ) | -19 | -16 | -12 | -9 | -6 | -3 | 1 | 4 | 7 | 10 | 13 | 17 | 20 | 23 | 26 | 30 |
| LIC | RDT (no TCZ) | -9 | -6 | -4 | -2 | 0 | 3 | 5 | 7 | 10 | 12 | 14 | 16 | 19 | 21 | 23 | 26 |
| LIC | RDT, PCR for negatives (no TCZ) | -40 | -37 | -33 | -30 | -27 | -23 | -20 | -17 | -13 | -10 | -7 | -4 | 0 | 3 | 6 | 10 |
| LIC | RDT, PCR for positives (no TCZ) | -9 | -7 | -6 | -4 | -3 | -1 | 1 | 2 | 4 | 5 | 7 | 8 | 10 | 12 | 13 | 15 |
| LIC | PCR (no TCZ) | -32 | -29 | -26 | -24 | -21 | -19 | -16 | -13 | -11 | -8 | -6 | -3 | -1 | 2 | 5 | 7 |
| LIC | No test, treat presumptively (TCZ) | -837 | -828 | -819 | -810 | -801 | -791 | -782 | -773 | -764 | -755 | -746 | -736 | -727 | -718 | -709 | -700 |
| LIC | RDT (TCZ) | -19 | -25 | -31 | -37 | -43 | -48 | -54 | -60 | -66 | -72 | -78 | -84 | -89 | -95 | -101 | -107 |
| LIC | RDT, PCR for negatives (TCZ) | -61 | -67 | -74 | -80 | -87 | -93 | -100 | -106 | -113 | -119 | -126 | -132 | -139 | -145 | -152 | -158 |
| LIC | RDT, PCR for positives (TCZ) | -9 | -15 | -21 | -27 | -33 | -39 | -45 | -51 | -57 | -63 | -69 | -75 | -81 | -87 | -93 | -99 |
| LIC | PCR (TCZ) | -42 | -49 | -55 | -62 | -68 | -75 | -82 | -88 | -95 | -102 | -108 | -115 | -122 | -128 | -135 | -142 |
| LMIC | No test, treat presumptively (no TCZ) | -73 | -58 | -43 | -28 | -13 | 2 | 17 | 32 | 47 | 62 | 77 | 92 | 107 | 122 | 137 | 152 |
| LMIC | RDT (no TCZ) | -13 | -2 | 9 | 19 | 30 | 41 | 52 | 63 | 74 | 85 | 96 | 107 | 117 | 128 | 139 | 150 |
| LMIC | RDT, PCR for negatives (no TCZ) | -48 | -34 | -20 | -6 | 8 | 22 | 35 | 49 | 63 | 77 | 91 | 105 | 119 | 133 | 147 | 161 |
| LMIC | RDT, PCR for positives (no TCZ) | -13 | -3 | 6 | 15 | 25 | 34 | 43 | 53 | 62 | 71 | 81 | 90 | 99 | 108 | 118 | 127 |
| LMIC | PCR (no TCZ) | -35 | -23 | -11 | 2 | 14 | 26 | 39 | 51 | 63 | 76 | 88 | 100 | 113 | 125 | 137 | 150 |
| LMIC | No test, treat presumptively (TCZ) | -892 | -858 | -824 | -790 | -756 | -722 | -688 | -654 | -619 | -585 | -551 | -517 | -483 | -449 | -415 | -381 |
| LMIC | RDT (TCZ) | -24 | -11 | 3 | 16 | 29 | 42 | 56 | 69 | 82 | 95 | 108 | 122 | 135 | 148 | 161 | 175 |
| LMIC | RDT, PCR for negatives (TCZ) | -69 | -52 | -35 | -18 | -1 | 16 | 33 | 50 | 67 | 84 | 101 | 118 | 135 | 151 | 168 | 185 |
| LMIC | RDT, PCR for positives (TCZ) | -13 | -2 | 10 | 21 | 32 | 43 | 55 | 66 | 77 | 89 | 100 | 111 | 122 | 134 | 145 | 156 |
| LMIC | PCR (TCZ) | -46 | -31 | -16 | -1 | 14 | 29 | 44 | 59 | 74 | 89 | 104 | 119 | 134 | 149 | 164 | 179 |
| UMIC | No test, treat presumptively (no TCZ) | -219 | -167 | -116 | -64 | -13 | 39 | 90 | 142 | 193 | 245 | 296 | 348 | 399 | 451 | 502 | 554 |
| UMIC | RDT (no TCZ) | -13 | 25 | 62 | 100 | 138 | 176 | 214 | 251 | 289 | 327 | 365 | 402 | 440 | 478 | 516 | 553 |
| UMIC | RDT, PCR for negatives (no TCZ) | -50 | -3 | 44 | 91 | 138 | 184 | 231 | 278 | 325 | 372 | 419 | 466 | 513 | 560 | 607 | 654 |
| UMIC | RDT, PCR for positives (no TCZ) | -11 | 23 | 57 | 90 | 124 | 158 | 191 | 225 | 259 | 292 | 326 | 360 | 393 | 427 | 461 | 494 |
| UMIC | PCR (no TCZ) | -38 | 5 | 48 | 91 | 134 | 177 | 219 | 262 | 305 | 348 | 391 | 433 | 476 | 519 | 562 | 605 |
| UMIC | No test, treat presumptively (TCZ) | -1038 | -931 | -824 | -717 | -610 | -503 | -396 | -289 | -181 | -74 | 33 | 140 | 247 | 354 | 461 | 568 |
| UMIC | RDT (TCZ) | -24 | 46 | 115 | 184 | 254 | 323 | 392 | 462 | 531 | 600 | 670 | 739 | 808 | 878 | 947 | 1017 |
| UMIC | RDT, PCR for negatives (TCZ) | -71 | 15 | 101 | 187 | 272 | 358 | 444 | 530 | 616 | 701 | 787 | 873 | 959 | 1045 | 1130 | 1216 |
| UMIC | RDT, PCR for positives (TCZ) | -11 | 51 | 113 | 176 | 238 | 300 | 362 | 424 | 486 | 548 | 610 | 672 | 734 | 797 | 859 | 921 |
| UMIC | PCR (TCZ) | -48 | 31 | 109 | 188 | 266 | 345 | 424 | 502 | 581 | 659 | 738 | 816 | 895 | 974 | 1052 | 1131 |

** PCR = polymerase chain reaction, RDT = rapid diagnostic tests, TCZ = tocilizumab*

### **A.3 Impact of influenza prevalence on net monetary benefits and the most cost-effective option**

S11 Table highlights the impact of influenza on the cost-effectiveness of different options, focusing on low-income countries and comparing presumptive treatment and RDT testing for low to moderate influenza prevalence, between 0-5%. RDT testing is cost-effective at all influenza prevalence levels for COVID-19 prevalence in severe COVID-like patients around 10% or higher. The impact of influenza prevalence on the net monetary benefit of RDT testing is relatively minor as testing helps substantially reduce the number of influenza cases unnecessarily treated with corticosteroids hence the negative impact of disease-specific side-effects. On the other hand, presumptive treatment is associated with a positive net monetary benefit at very low influenza prevalence, when it is higher than the net monetary benefit associated with RDT testing (hence presumptive treatment is more cost-effective) but its net monetary benefit quickly declines with influenza prevalence and is negative at all SARS-CoV-2 prevalence values except 30% when influenza prevalence reaches 5%. In this context, presumptive treatment is then worse than ignoring COVID. These results suggest that, when influenza prevalence is low but there is uncertainty around its value (a plausible situation in low-income countries in which surveillance systems are often not well-resourced) RDT testing is a less risky option than presumptive treatment.

**S11 Table: Average net monetary benefit associated with RDT testing and presumptive treatment in low-income countries (reference: no testing or treatment) for different, low influenza prevalence levels between 0 and 5% of severe COVID-like, non-COVID patients**

| Scenario | Influenza prevalence* | COVID-19 prevalence among severe "COVID-like" patients (suspected cases) | | | | | | | | | | | | | | | |
| --- | --- | --- | --- | --- | --- | --- | --- | --- | --- | --- | --- | --- | --- | --- | --- | --- | --- |
|  |  | 0% | 2% | 4% | 6% | 8% | 10% | 12% | 14% | 16% | 18% | 20% | 22% | 24% | 26% | 28% | 30% |
| Presumptive treatment | 0% | -9 | -6 | -3 | 0 | 3 | 6 | 9 | 12 | 15 | 18 | 21 | 24 | 27 | 30 | 33 | 36 |
| Presumptive treatment | 1% | -19 | -16 | -12 | -9 | -6 | -3 | 1 | 4 | 7 | 10 | 13 | 17 | 20 | 23 | 26 | 30 |
| Presumptive treatment | 2% | -28 | -25 | -21 | -18 | -14 | -11 | -8 | -4 | -1 | 3 | 6 | 9 | 13 | 16 | 20 | 23 |
| Presumptive treatment | 3% | -37 | -34 | -30 | -27 | -23 | -19 | -16 | -12 | -9 | -5 | -1 | 2 | 6 | 9 | 13 | 17 |
| Presumptive treatment | 4% | -47 | -43 | -39 | -35 | -31 | -28 | -24 | -20 | -16 | -13 | -9 | -5 | -1 | 3 | 6 | 10 |
| Presumptive treatment | 5% | -56 | -52 | -48 | -44 | -40 | -36 | -32 | -28 | -24 | -20 | -16 | -12 | -8 | -4 | 0 | 4 |
| RDT testing | 0% | -9 | -6 | -4 | -2 | 1 | 3 | 5 | 7 | 10 | 12 | 14 | 17 | 19 | 21 | 23 | 26 |
| RDT testing | 1% | -9 | -6 | -4 | -2 | 0 | 3 | 5 | 7 | 10 | 12 | 14 | 16 | 19 | 21 | 23 | 26 |
| RDT testing | 2% | -9 | -7 | -4 | -2 | 0 | 3 | 5 | 7 | 9 | 12 | 14 | 16 | 19 | 21 | 23 | 25 |
| RDT testing | 3% | -9 | -7 | -4 | -2 | 0 | 3 | 5 | 7 | 9 | 12 | 14 | 16 | 19 | 21 | 23 | 25 |
| RDT testing | 4% | -9 | -7 | -4 | -2 | 0 | 2 | 5 | 7 | 9 | 12 | 14 | 16 | 18 | 21 | 23 | 25 |
| RDT testing | 5% | -9 | -7 | -5 | -2 | 0 | 2 | 5 | 7 | 9 | 11 | 14 | 16 | 18 | 21 | 23 | 25 |

** among severe COVID-like, non-COVID patients. RDT = rapid diagnostic tests.*

When influenza prevalence increases further, the most cost-effective testing options change. S3 Figure compares, on the same graph, 5% influenza, 10% influenza, 20% influenza and 30% influenza prevalence among severe, COVID-like non-COVID patients. In low-income countries, the most cost-effective options are the same across all moderate to high influenza prevalence levels: at low SARS-CoV-2 prevalence, treating patients as non-COVID (in which case they may be given oxygen supplementation and symptomatic treatment as per need, and may be assessed for other diseases) is the most cost-effective option, while at high prevalence testing patients with RDTs to inform treatment is the most cost-effective. In upper-middle-income countries, testing is the most cost-effective option at virtually all SARS-CoV-2 prevalence levels. At high prevalence, the most cost-effective option is to test with RDTs and check negative test results with PCR, while at low prevalence levels, testing with RDTs (the most cost-effective option at 5% flu), is progressively replaced by more specific testing algorithms (confirming positive test results with PCR, and at very high prevalence, PCR alone) as influenza prevalence increases.

**S3 Figure: Impact of increasing values of influenza prevalence on cost-effectiveness**


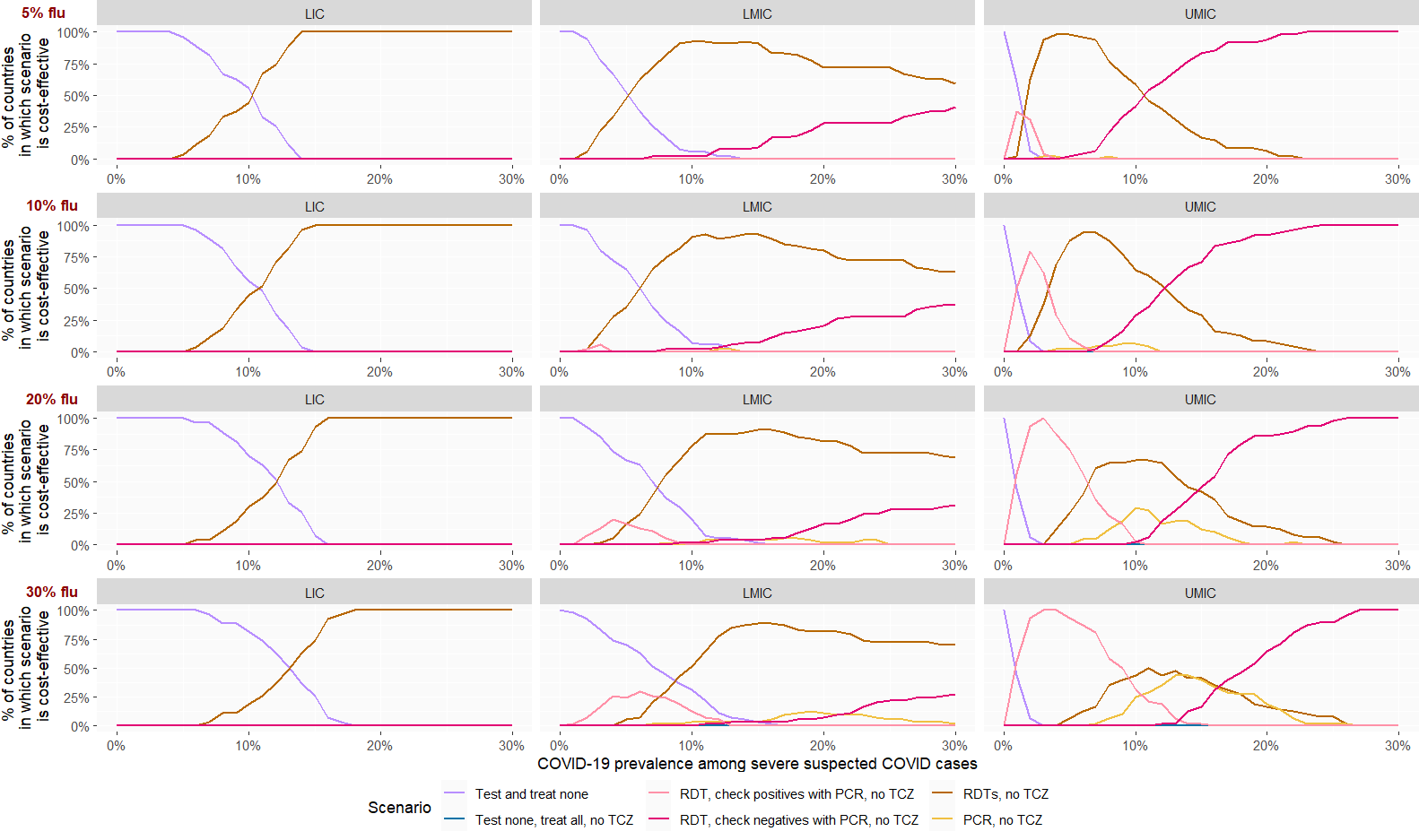


** PCR = polymerase chain reaction, RDT = rapid diagnostic tests, TCZ = tocilizumab*

### **A.4 Influenza and SARS-CoV-2 prevalence thresholds associated with changes in the most cost-effective option**

The cost-effectiveness of testing is highly influenced by two parameters: SARS-CoV-2 prevalence in the target population (patients with severe COVID-like disease), and influenza prevalence in this population.

S4 Figure shows that testing is the most cost-effective option (for at least certain values of SARS-CoV-2 prevalence) in all countries when influenza in the target population is at least 2.6%. The threshold willingness to pay at which testing is the most cost-effective option increases with decreasing influenza prevalence.

**S4 Figure: Minimum influenza prevalence in patients with severe COVID-like, non-COVID disease beyond which testing is the most cost-effective option for at least some SARS-CoV-2 prevalence levels, as a function of the cost-effectiveness threshold (each dot represents a country)**

** LIC = low-income country, LMIC = lower-middle-income country, UMIC = upper-middle-income country*

Confirming negative test results with PCR is cost-effective at high SARS-CoV-2 prevalence in upper-middle-income countries and some lower-middle-income countries (S2 and S3 Figure). S5 Figure shows the impact of a country’s cost-effectiveness threshold on whether confirming negative test results is the most cost-effective option at high SARS-CoV-2 prevalence. Confirming negatives with PCR becomes cost-effective when the cost-effectiveness threshold exceeds a value around $950, a value almost independent of influenza prevalence.

**S5 Figure: Cost-effectiveness of confirming negative test results when SARS-CoV-2 prevalence is high as a function of a country’s cost-effectiveness threshold (in US$)*, for 1% influenza in patients with COVID-like non-COVID disease**

**
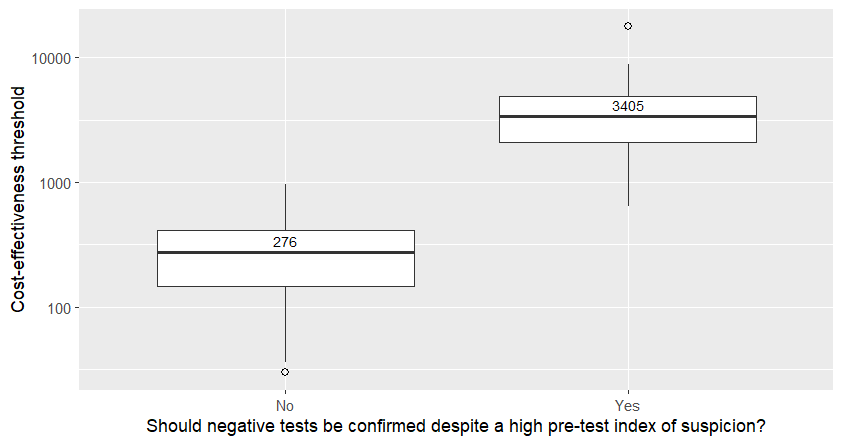
**

**The y-axis is on a logarithmic scale*

Confirming positive test results with a low index of suspicion is cost-effective, mostly in upper-middle-income countries and when there is moderate to high influenza prevalence. S6 Figure shows that this option becomes cost-effective at different influenza prevalence levels depending on a country’s cost-effectiveness threshold. The median of the influenza prevalences on the graph is 10%. Confirming test results becomes cost-effective at a higher influenza prevalence for a lower cost-effectiveness threshold. Note that all countries on the graph have a cost-effectiveness threshold above $800: indeed, confirming positive test results is never the most cost-effective option in countries with a cost-effectiveness threshold below that value.

**S6 Figure: Minimum influenza prevalence in patients with COVID-like (non-COVID) severe disease beyond which patients with a low initial level of suspicion for COVID who test positive on RDTs should be confirmed by PCR, as a function of the cost-effectiveness threshold (each dot represents a country)**

** LIC = low-income country, LMIC = lower-middle-income country, UMIC = upper-middle-income country.*

In most low- and lower-middle-income countries, the two primary options at any influenza prevalence are: treating patients as if they did not have COVID, and testing patients with RDTs to inform treatment. S7 Figure shows that the SARS-CoV-2 prevalence threshold beyond which RDT testing becomes cost-effective is roughly the inverse of a country’s cost-effectiveness threshold. In upper-middle-income countries, testing is cost-effective in virtually any context (SARS-CoV-2 prevalence in COVID-like patients below 3%), whereas the index of suspicion needs to be much larger to justify testing in low-income countries.

**S7 Figure: Minimum SARS-CoV-2 prevalence in suspected cases beyond which testing for COVID becomes cost-effective (for 10% influenza prevalence), as a function of the cost-effectiveness threshold, in $ (each dot represents a country)**

** LIC = low-income country, LMIC = lower-middle-income country, UMIC = upper-middle-income country.*

Finally, S8 Figure shows how the COVID thresholds beyond which negative test results should be confirmed with PCR. In line with S5 Figure, countries with low cost-effectiveness thresholds (roughly below $900) do not appear on this graph as confirming negative test results with PCR is not the most cost-effective option in those countries. The SARS-CoV-2 prevalence threshold for confirming negative test results ranges from 7% (for the country with the highest cost-effectiveness threshold) to almost 30%, with a median value of 14%.

**S8 Figure: Minimum SARS-CoV-2 prevalence among severe COVID-like patients beyond which negative COVID RDT test results should be confirmed with PCR, for 10% influenza prevalence (each dot represents a country)**

** LIC = low-income country, LMIC = lower-middle-income country, UMIC = upper-middle-income country*

The threshold SARS-CoV-2 prevalence beyond which which confirming negative SARS-CoV-2 tests with PCR is the most cost-effective option increases with decreasing willingness to pay. Low-income countries do not appear on this graph because confirming negative test results is not cost-effective in those contexts.

## **B. Impact of TCZ costs on the cost-effectiveness of testing and estimates of the maximum cost of a TCZ treatment course allowing for its use in severe/critical COVID patients to be cost-effective**

The use of TCZ supported by testing is never cost-effective in low-income countries at median TCZ costs (Figure 5 of the main paper). Here, we first explore whether, using the extremes of the range of cost values found within the literature, the use of TCZ (supported by testing) may be more/less likely to be cost-effective. We find that the main change between the different panels takes place in the lower-middle-income country (LMIC) category, with limited-to-no change for low-income and upper-middle-income countries.

**S9 Figure: Comparison of scenarios with different TCZ costs ($411 – panel A, $861.5 – Panel B, and $1207 – Panel C)**


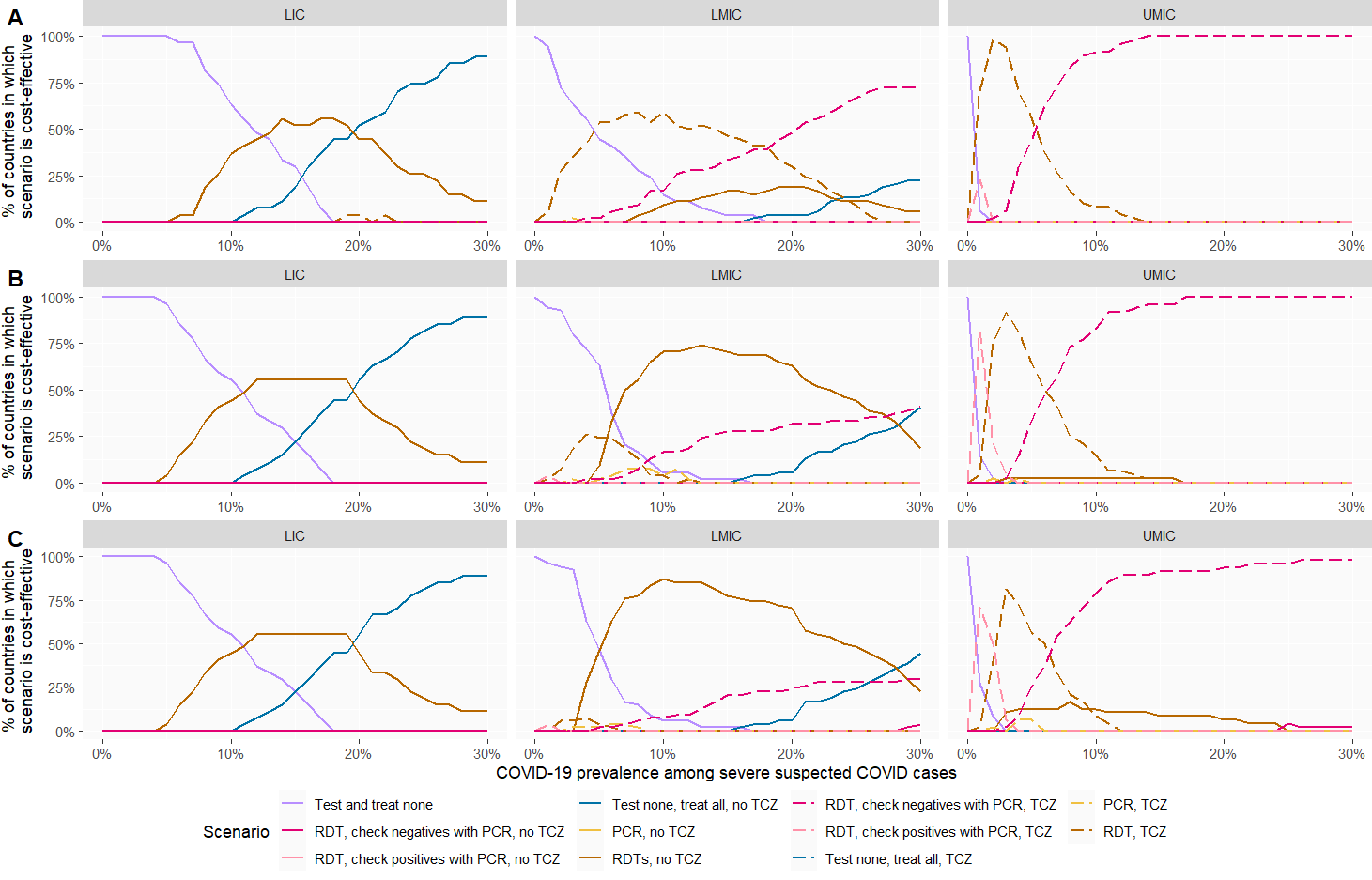


** PCR = polymerase chain reaction, RDT = rapid diagnostic tests, TCZ = tocilizumab*

We have therefore calculated what costs of a TCZ treatment course would allow its use to be cost-effective (under any testing scenarios) in a given country and for a given prevalence level. These values have been obtained based on the results of our model at baseline TCZ costs. We used the net monetary benefit and numbers treated with TCZ associated with each testing/treatment scenario. We calculated which TCZ treatment cost would allow for the net monetary benefit of any one of the options associated with TCZ use to equate the maximum net monetary benefit across all testing scenarios not involving TCZ. This is the maximum TCZ cost allowing for the use of TCZ to be cost-effective. Those results are presented in S10 Figure below. The TCZ treatment costs for which TCZ use would be cost-effective increase with prevalence as more people benefit from treatment for each person tested. At high prevalence, acceptable TCZ cost plateau, as the cost of testing becomes negligible as compared to the cost of treatment and the model is actually assessing whether TCZ treatment is cost-effective.

**S10 Figure: Maximum tocilizumab (TCZ) treatment cost allowing its use to be cost-effective (median values across country income groups)**

## **C. Other sensitivity analyses not presented in the main paper**

This section includes the graphs not presented in the main paper and representing the most cost-effective options under different scenarios. As per our methodology, the “most cost-effective option” is the option most likely to be cost-effective based on Monte Carlo simulation of 1000 parameter sets. Results are shown as a share of countries at a given income level for which a specific scenario is the most cost-effective.

### **S11 Figure: Share of countries for which specific options are the most cost-effective in the absence of PCR (TCZ unavailable)**


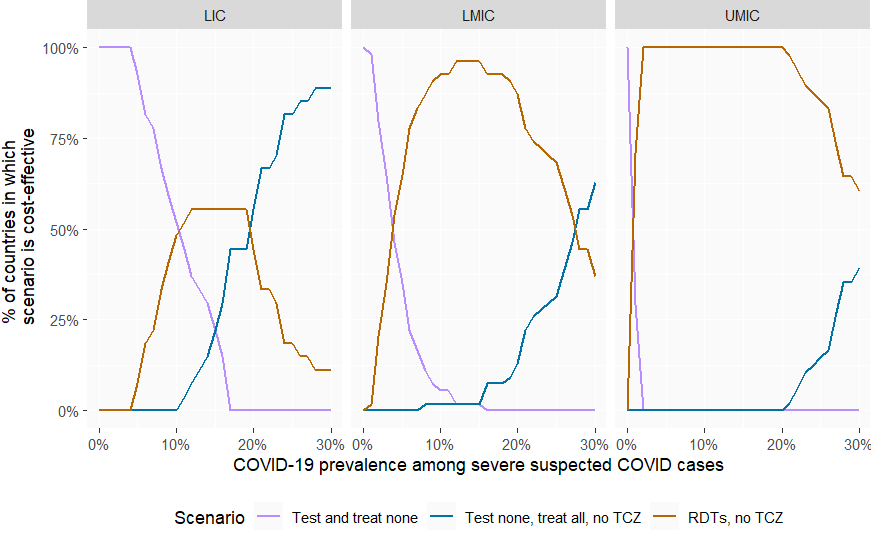


** PCR = polymerase chain reaction, RDT = rapid diagnostic tests, TCZ = tocilizumab*

### **S12 Figure: Share of countries for which specific options are the most cost-effective in the absence of mechanical ventilation (TCZ unavailable)**


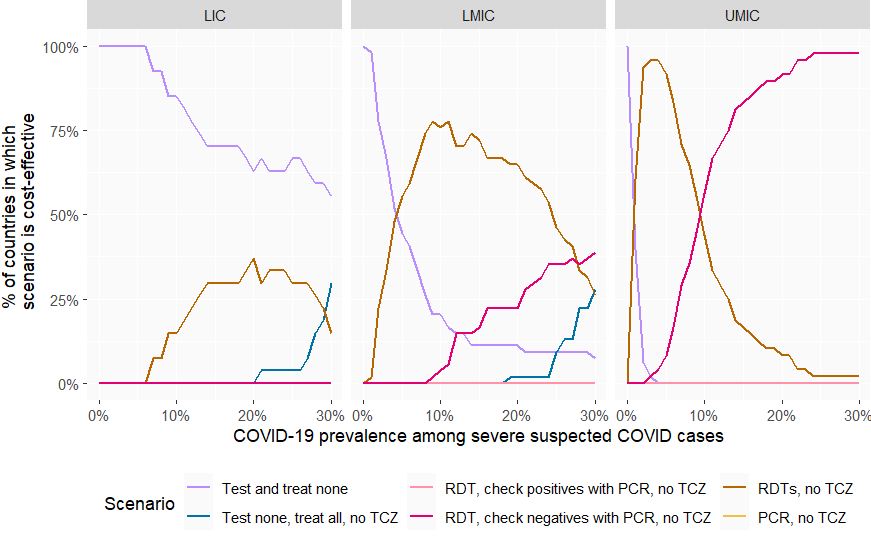


** PCR = polymerase chain reaction, RDT = rapid diagnostic tests, TCZ = tocilizumab*

### **S13 Figure: Proportion of countries in which a given option is the most cost-effective if the cost of treating corticosteroid side-effects is low (panel A) or high (panel B)**


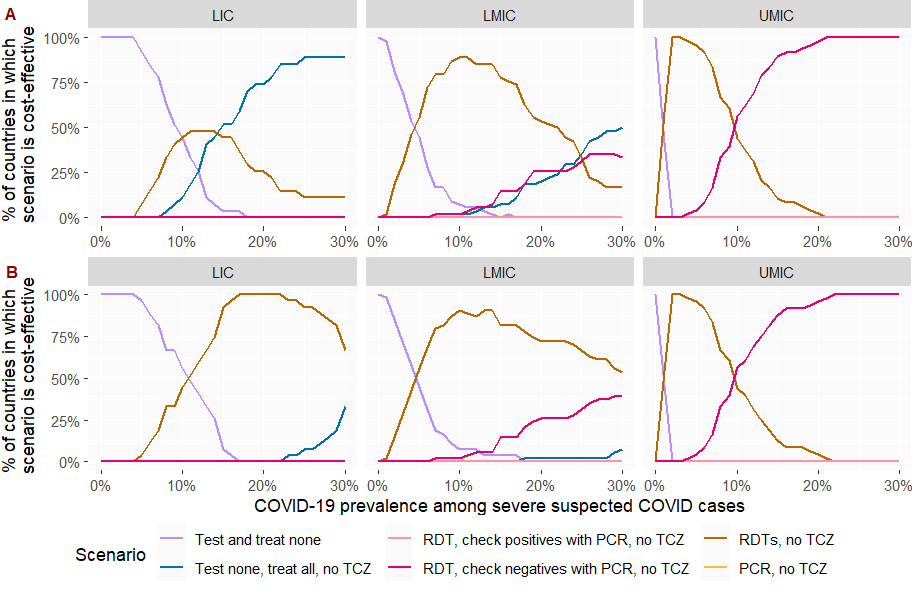


** PCR = polymerase chain reaction, RDT = rapid diagnostic tests, TCZ = tocilizumab*

### **S14 Figure: Proportion of countries in which a given option is the most cost-effective accounting for 10% influenza prevalence, if TCZ are available**

**
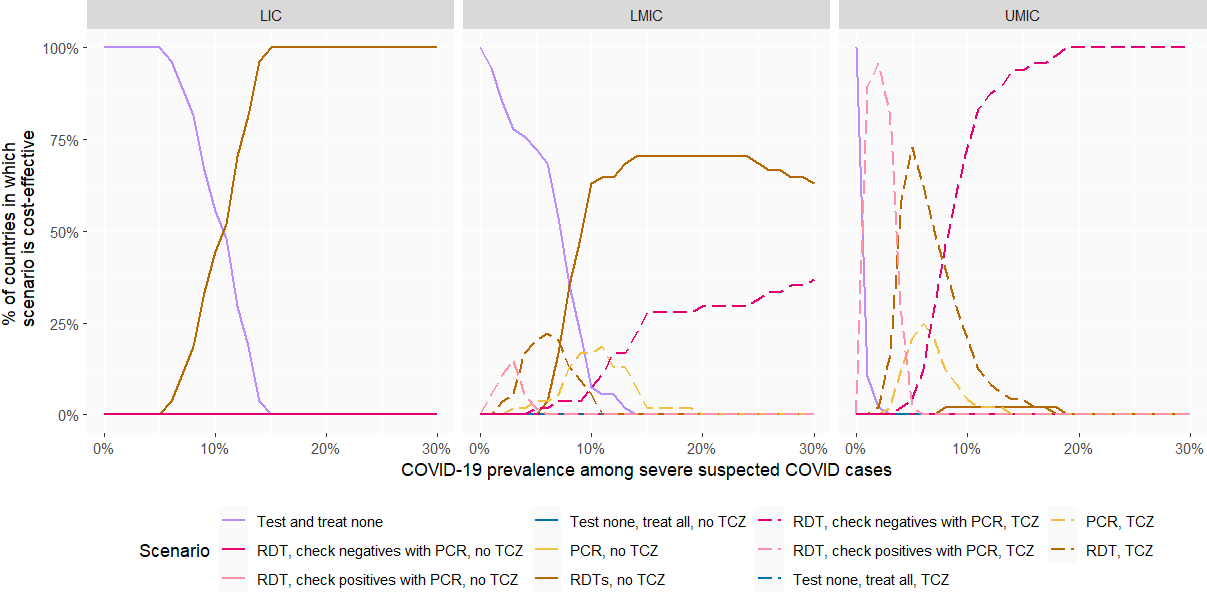
**

** PCR = polymerase chain reaction, RDT = rapid diagnostic tests, TCZ = tocilizumab*

### **S15 Figure: Proportion of countries in which a given option is the most cost-effective if treatment changes post-COVID likelihood and/or severity, by a 20% decrease (panel A) or increase (panel B) in associated DALYs per surviving patient, if TCZ is not available**

**
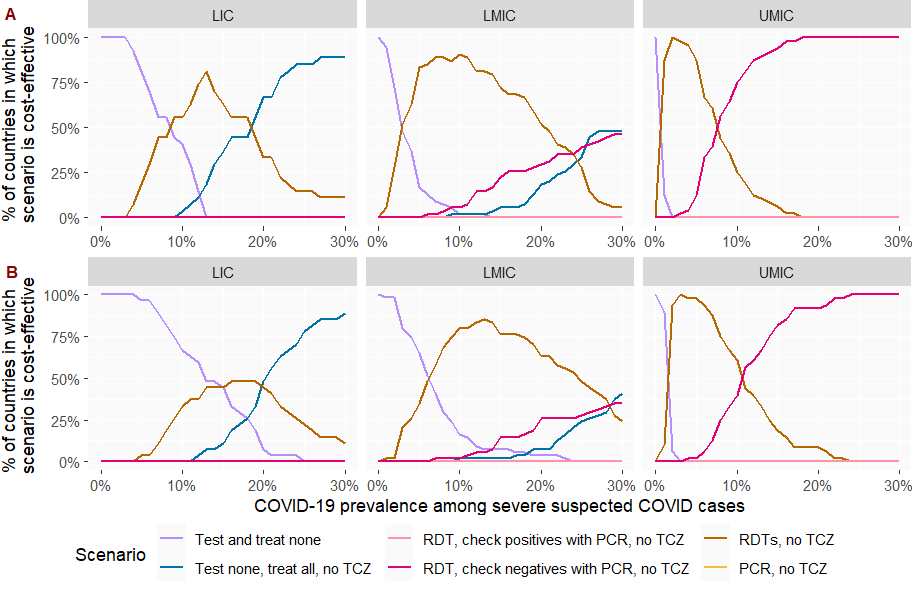
**

** PCR = polymerase chain reaction, RDT = rapid diagnostic tests, TCZ = tocilizumab*

### **S16 Figure: Proportion of countries in which a given option is the most cost-effective if treatment changes post-COVID likelihood and/or severity, by a 20% decrease (panel A) or increase (panel B) in associated DALYs per surviving patient, if TCZ is available**

**
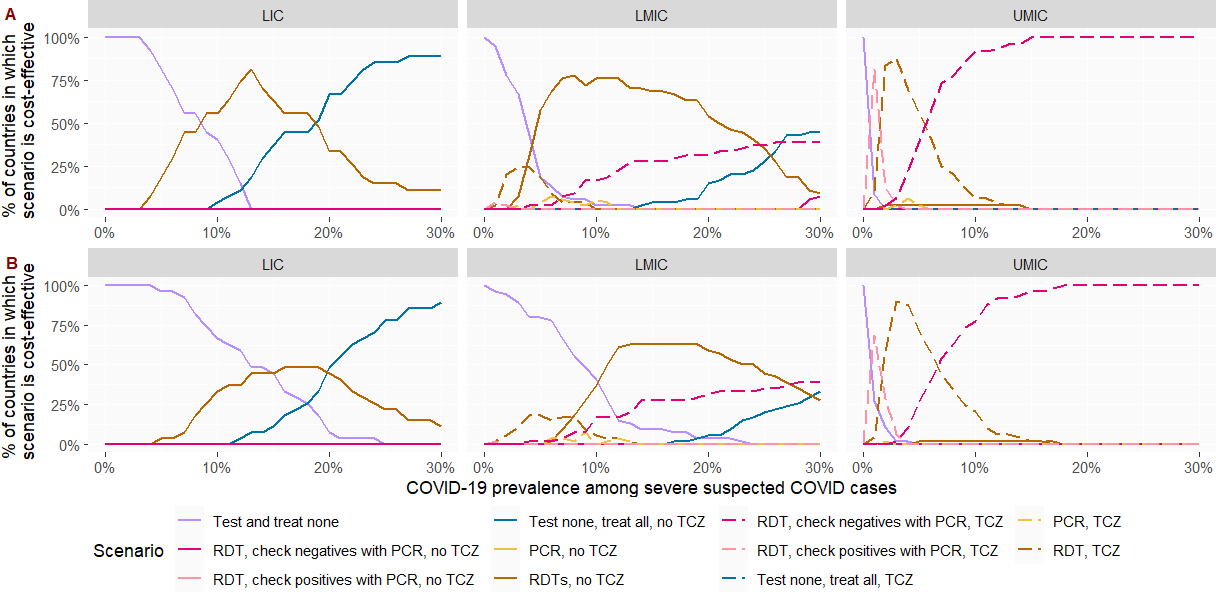
**

** PCR = polymerase chain reaction, RDT = rapid diagnostic tests, TCZ = tocilizumab*

### **S17 Figure: Proportion of countries in which a given option is the most cost-effective at $6.2 (panel A) or $0.6 (panel B) per RDT test kit (no TCZ)**

**
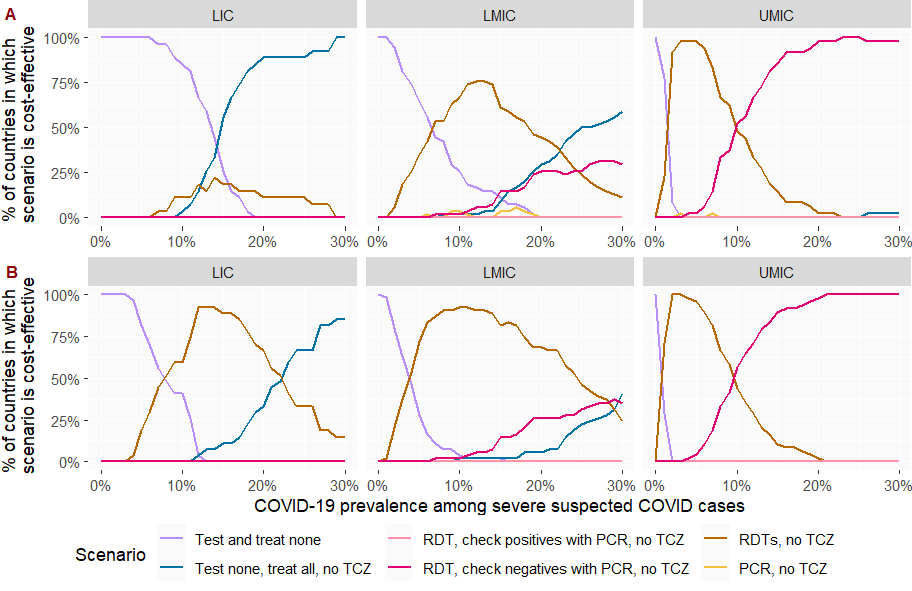
**

** PCR = polymerase chain reaction, RDT = rapid diagnostic tests, TCZ = tocilizumab*

S17 highlights that relatively more expensive test kits (up to $6.2 per kit) remain cost-effective in upper-middle-income countries, but testing would cease to be cost-effective, if RDTs are sold at that price, in most low-income countries, at least at low influenza prevalence levels (1% in the figure).

### **S18 Figure: Proportion of countries in which a given option is the most cost-effective at $6.2 (panel A) or $0.6 (panel B) per RDT test kit (TCZ available)**


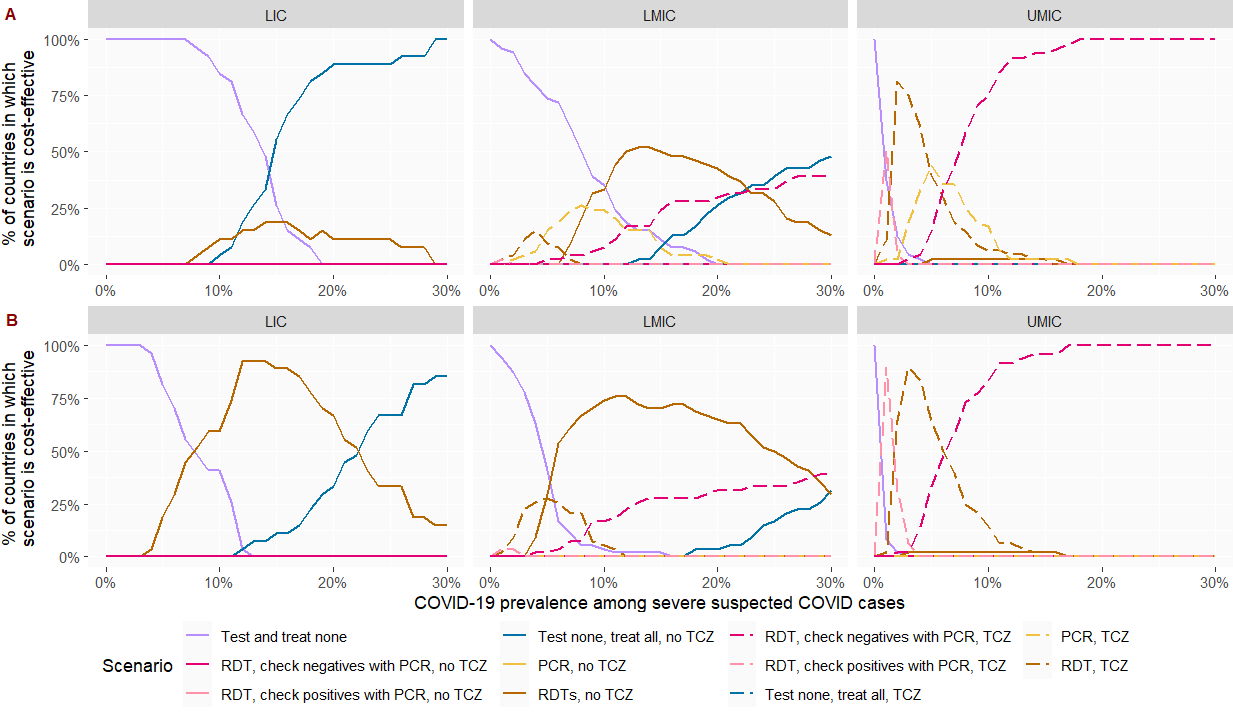


** PCR = polymerase chain reaction, RDT = rapid diagnostic tests, TCZ = tocilizumab. Dotted lines are used to represent options in which TCZ is used, whereas full lines represent options in which it is not.*

**S19 Figure: Proportion of countries in which a given option is the most cost-effective if RDT sensitivity is 90% (panel A), 60% (panel B) or 40% (panel C), no TCZ**

**
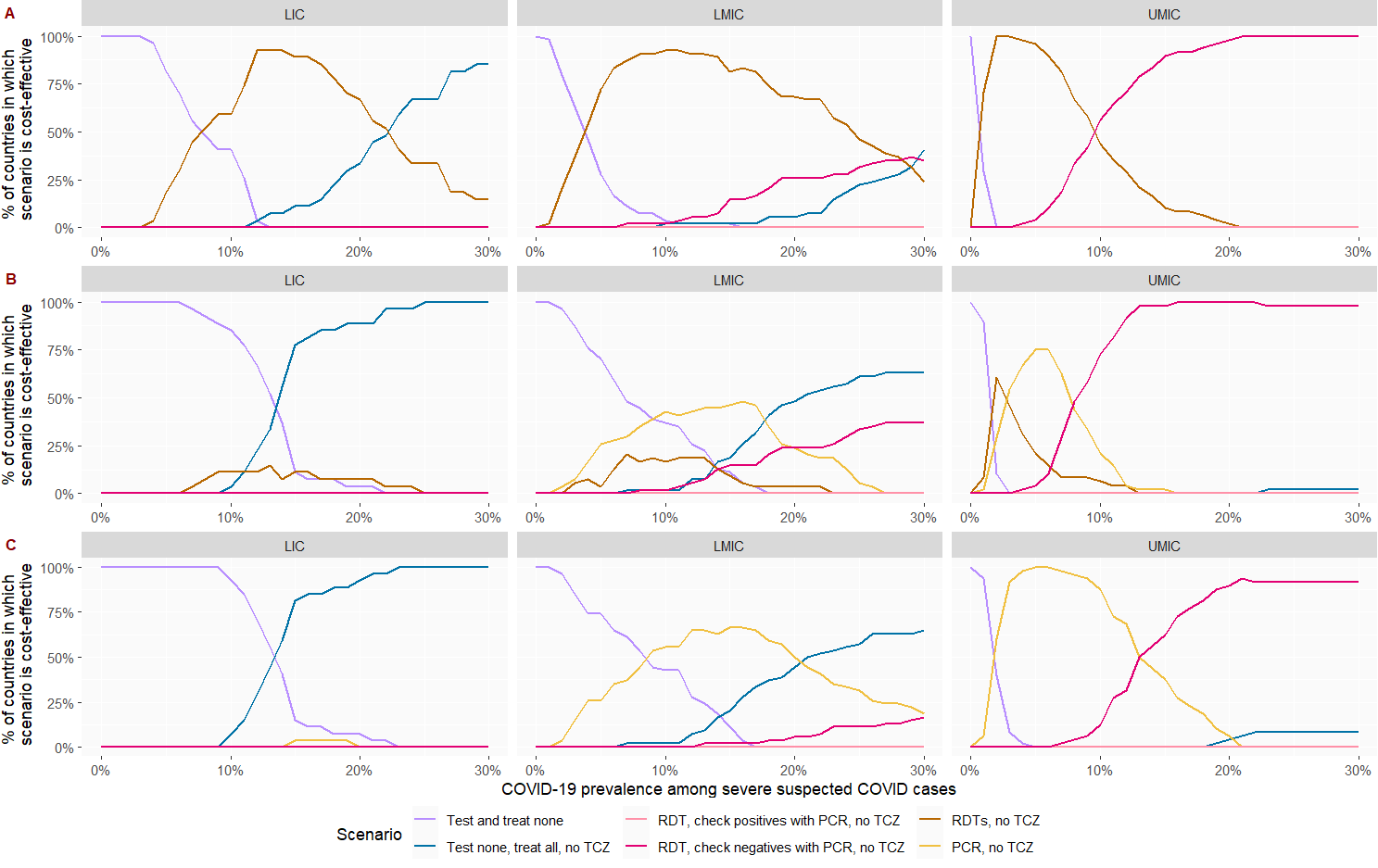
**

** PCR = polymerase chain reaction, RDT = rapid diagnostic tests, TCZ = tocilizumab*

S19 figure highlights the impact of test sensitivity on model outcomes. With higher sensitivity than at baseline (panel A), testing becomes cost-effective in a broader range of contexts. As test sensitivity declines, testing ceases to be cost-effective almost entirely (at 1% influenza prevalence) in low-income countries, while in countries with higher income levels that can afford it, the use of PCR instead of RDT becomes the most cost-effective option.

### **S20 Figure: Proportion of countries in which a given option is the most cost-effective if RDT sensitivity is 90% (panel A), 60% (panel B) or 40% (panel C), TCZ available**

**
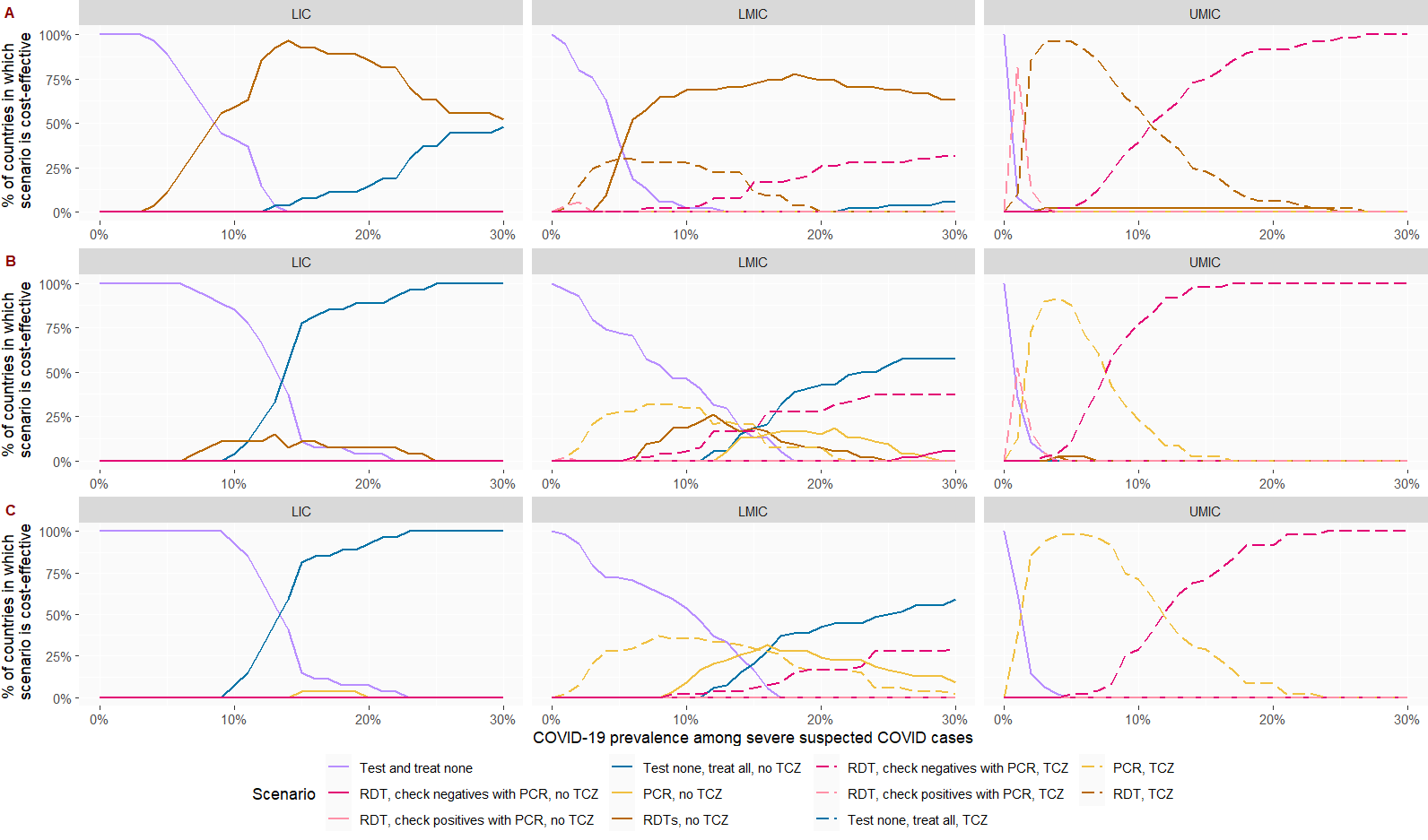
**

** PCR = polymerase chain reaction, RDT = rapid diagnostic tests, TCZ = tocilizumab. Dotted lines are used to represent options in which TCZ is used, whereas full lines represent options in which it is not.*

### **S21 Figure: Proportion of countries in which a given option is the most cost-effective if 30% (panel A) or 60% (panel B) of patients refuse treatment (no TCZ)**

**
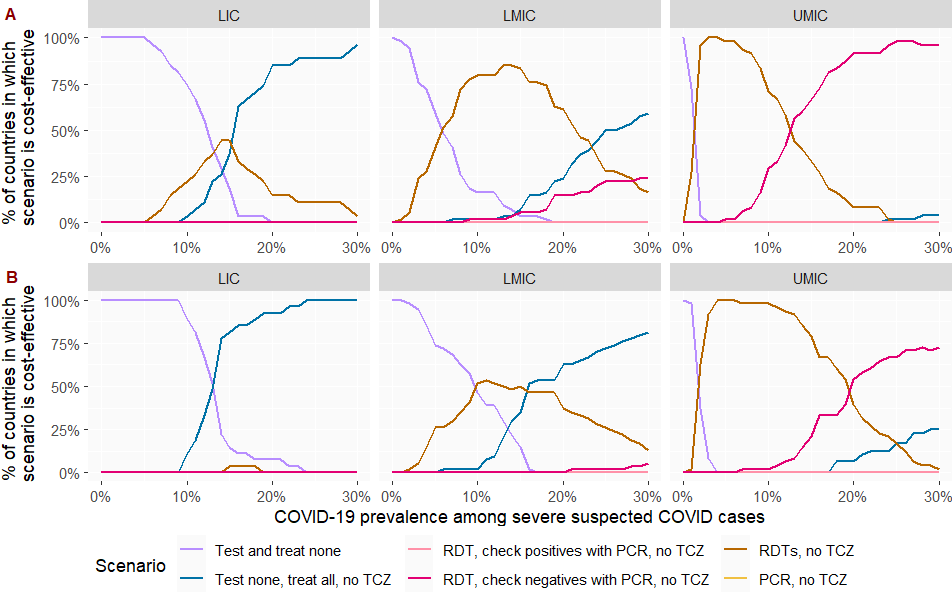
**

** PCR = polymerase chain reaction, RDT = rapid diagnostic tests, TCZ = tocilizumab*

### **S22 Figure: Proportion of countries in which a given option is the most cost-effective if 30% (panel A) or 60% (panel B) of patients refuse treatment (TCZ available)**

**
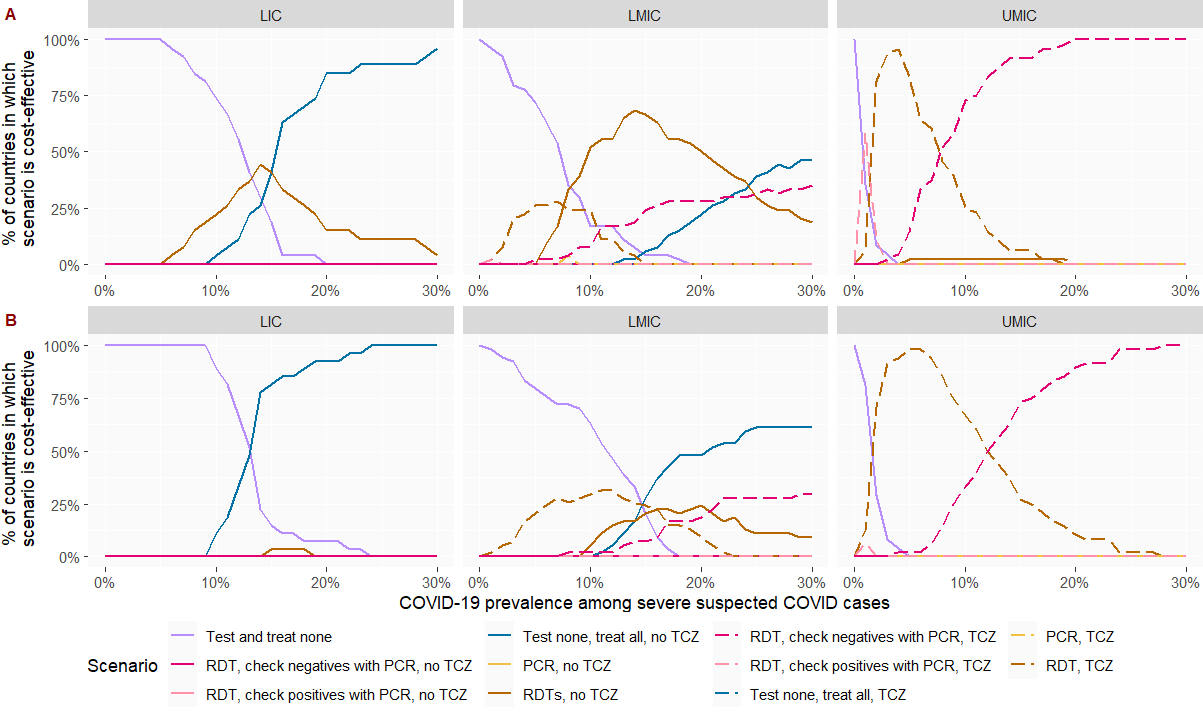
**

** PCR = polymerase chain reaction, RDT = rapid diagnostic tests, TCZ = tocilizumab. Dotted lines are used to represent options in which TCZ is used, whereas full lines represent options in which it is not.*

### **S23 Figure: Proportion of countries in which a given option is the most cost-effective if treatment involves important additional clinical screening costs (TCZ unavailable)**

**
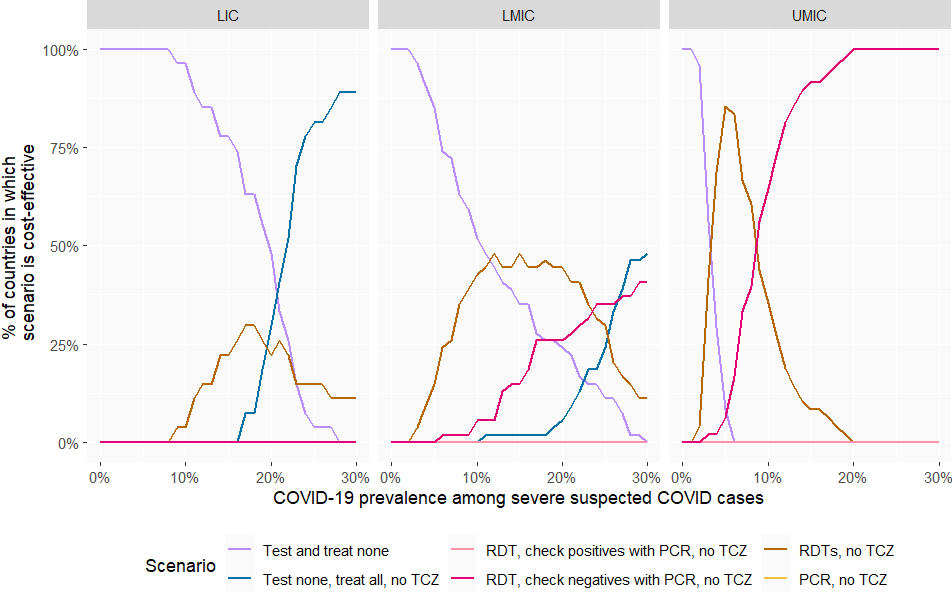
**

** PCR = polymerase chain reaction, RDT = rapid diagnostic tests, TCZ = tocilizumab*

### **S24 Figure: Proportion of countries in which a given option is the most cost-effective if treatment involves important additional clinical screening costs (TCZ available)**


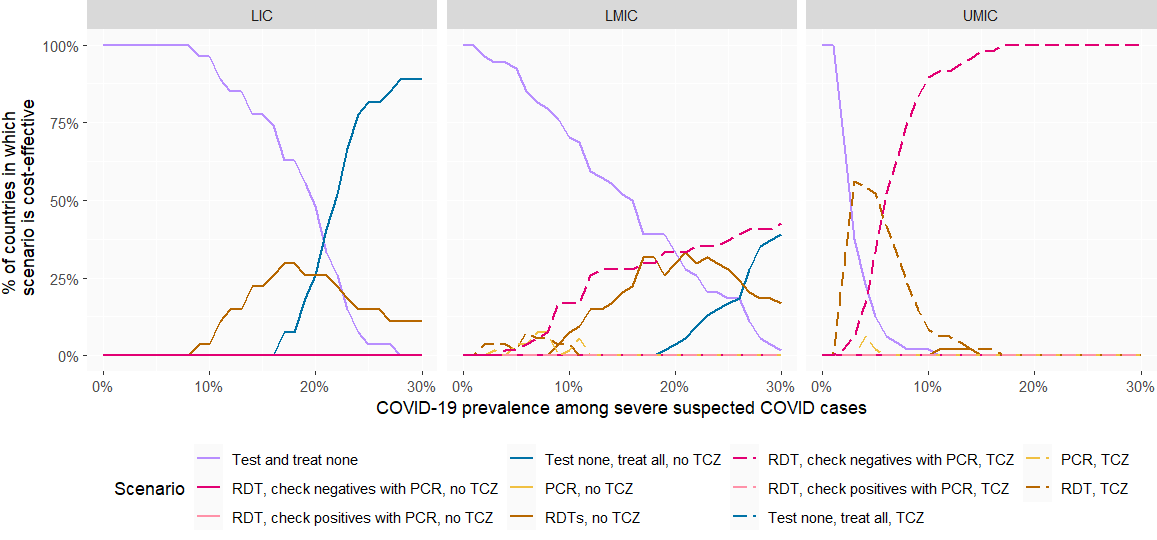


** PCR = polymerase chain reaction, RDT = rapid diagnostic tests, TCZ = tocilizumab. Dotted lines are used to represent options in which TCZ is used, whereas full lines represent options in which it is not.*

### **S37 Figure: Proportion of countries in which a given option is the most cost-effective with 3% health discounting (TCZ unavailable)**


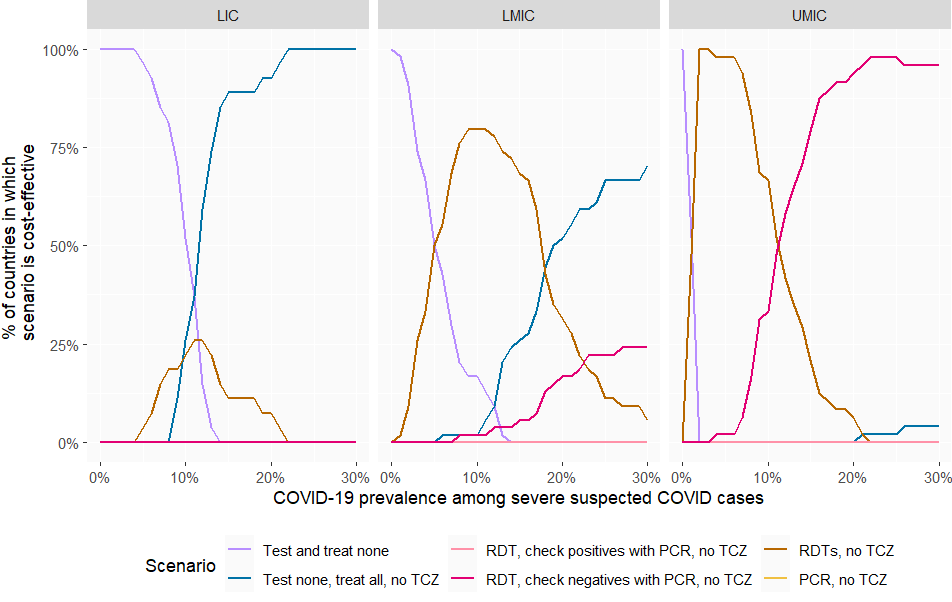


** PCR = polymerase chain reaction, RDT = rapid diagnostic tests, TCZ = tocilizumab*

### **S38 Figure: Proportion of countries in which a given option is the most cost-effective with 3% health discounting (TCZ available)**


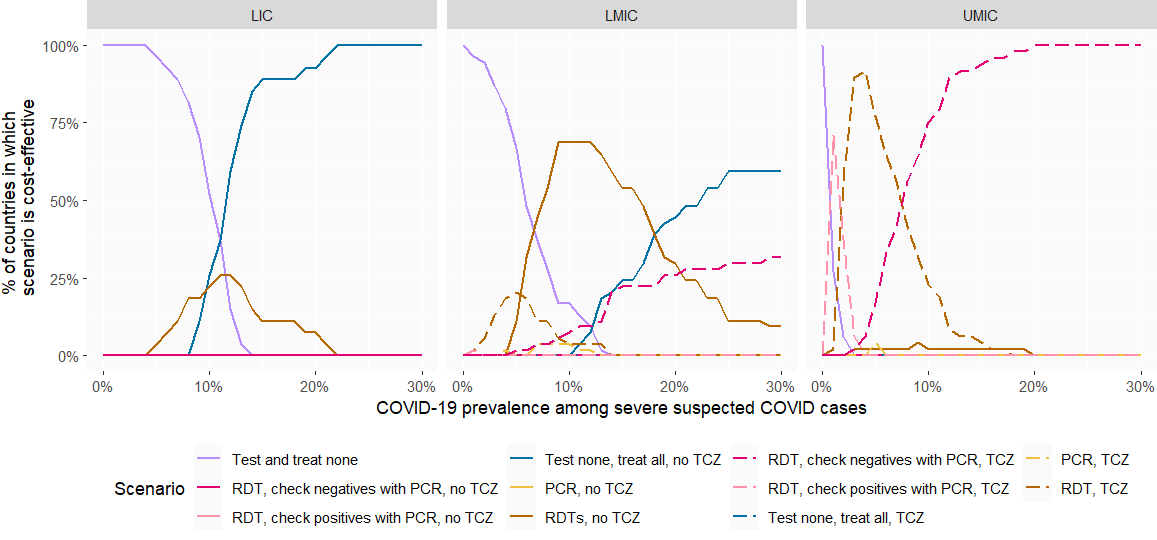


## ** PCR = polymerase chain reaction, RDT = rapid diagnostic tests, TCZ = tocilizumab. Dotted lines are used to represent options in which TCZ is used, whereas full lines represent options in which it is not.*

## **D. Tornado graph**

S39 Figure represents the impact of variability (i.e., difference in parameters across contexts, for example, the cost of a day in a hospital bed or the value of SARS-CoV-2 prevalence) and uncertainty (i.e., lack of precision in our knowledge of e.g., current case fatality rates with COVID) on net health benefit (defined as the net monetary benefit divided by the cost-effectiveness threshold). Each value represents the impact of a difference of one standard deviation in the corresponding parameter on the net health benefits of testing as compared to no testing (most cost effective option involving testing vs. most cost-effective option not involving testing). We have used the distributions defined in S1 appendix for all parameters, except for parameters which did not have a pre-defined distribution, such as SARS-CoV-2 prevalence, because we had explored a range of values. For such parameters, we have defined a distribution for the purpose of developing this graph. We have used uniform distributions for COVID and influenza prevalence, across the whole range that we explored (0-30%). Further, for variables such as TCZ cost (where we used a median value at baseline then explored a high and low value), we used a uniform distribution between the low/high boundary of the range. Triangular distributions were used for variables for which our baseline estimate was very far from the mid-point between the low and high values we explored (e.g., for the cost of RDT test kits). The impact of between-country variability in country-specific parameters is represented in dark blue on the graph, as is the variability in COVID and influenza prevalence across contexts. Meanwhile, the impact of the uncertainty in specific parameters on the uncertainty on net monetary benefits is represented in light blue.

The graph shows that the largest drivers of the variability in the net health benefits of testing relate to a country’s resource level, namely differences between countries in the cost of hospitalization – in the general ward and in ICU. The variability in SARS-CoV-2 prevalence is the next largest driver of variability in net health benefits of testing. Variability in the cost of treating corticosteroid side-effects is the next larger driver of between-country differences. Availability of TCZ is also a major driver.

The largest driver of uncertainty on the net health benefits of testing is uncertainty on COVID case fatality rates. While the analysis considered uncertainty on current case fatality rates, there could further be variability in the future in those rates as the virus and population immunity evolve. Other important sources of uncertainty include uncertainties on the impacts of corticosteroids and on the cost of a day in ICU.

**S39 figure: Tornado graph – impact of heterogeneity and uncertainty on parameters in indicator values on the variability in the net health benefit of testing compared to no testing**


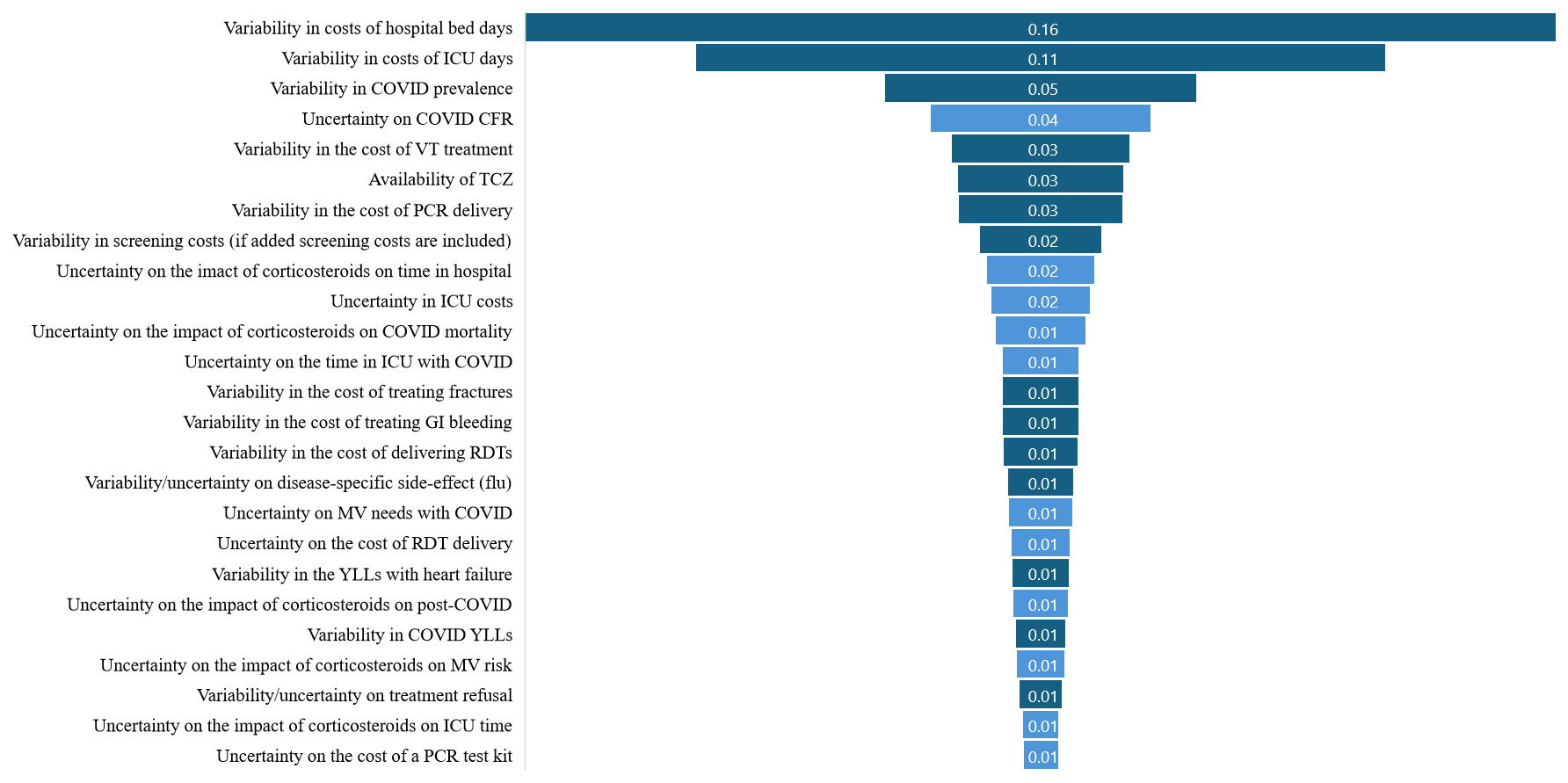


** CFR = case fatality rate, GI = gastrointestinal, ICU = intensive care unit, MV = mechanical ventilation, PCR =* *polymerase chain reaction, RDT = rapid diagnostic test, TCZ = tocilizumab, VT = venous thrombosis, YLL = year of life lost. The size of the bars represents the change in net health benefits associated with a one standard deviation change in each parameter (based on calculations on 1000 samples).*
