# Supplementary material for: Cost-effectiveness of COVID rapid diagnostic tests for patients with severe/critical illness in low- and middle-income countries: A modeling study
Source: PLoS Med. 2024 Jul 18;21(7):e1004429. doi: 10.1371/journal.pmed.1004429 (PMC11293649; doi:10.1371/journal.pmed.1004429)
Supplement: S4 Appendix — (DOCX) [file pmed.1004429.s004.docx]

# **S4 Appendix: Country-specific graphs**

In the graphs below, we show the probability, for each country, that each of the testing/treatment scenarios be the most cost-effective, depending on the prevalence of COVID-19 among severe patients with suspected COVID. As for other graphs, this is based on 1000 Monte Carlo samples using Latin Hypercube Sampling of the parameter distributions in S2 Appendix. Individual country names are not provided to avoid giving a false impression of accuracy when country-specific values of parameters such as RDT sensitivity, cost of treatment side effects and TCZ price should be identified and used.

**S40 Figure: Probability for each scenario to be the most cost-effective, by country, low-income countries (TCZ available)**


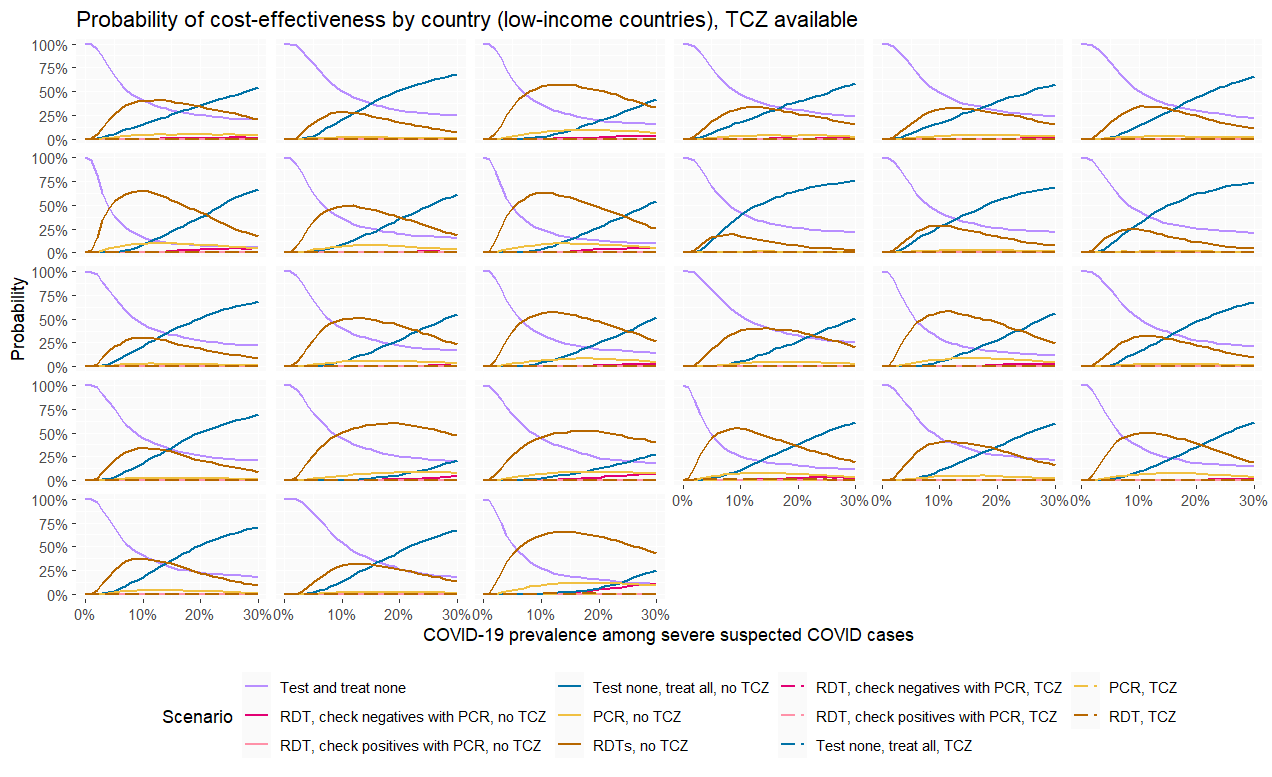


** PCR = polymerase chain reaction, RDT = rapid diagnostic tests, TCZ = tocilizumab. Dotted lines are used to represent options in which TCZ is used, whereas full lines represent options in which it is not.*

This graph is the same as the graph for situations when TCZ is not available (S42 Figure): indeed, at medium costs, the use of TCZ has a 0% chance of being cost-effective in low-income countries.

**S41 Figure: Probability for each scenario to be the most cost-effective, by country, lower-middle-income countries (TCZ available)**


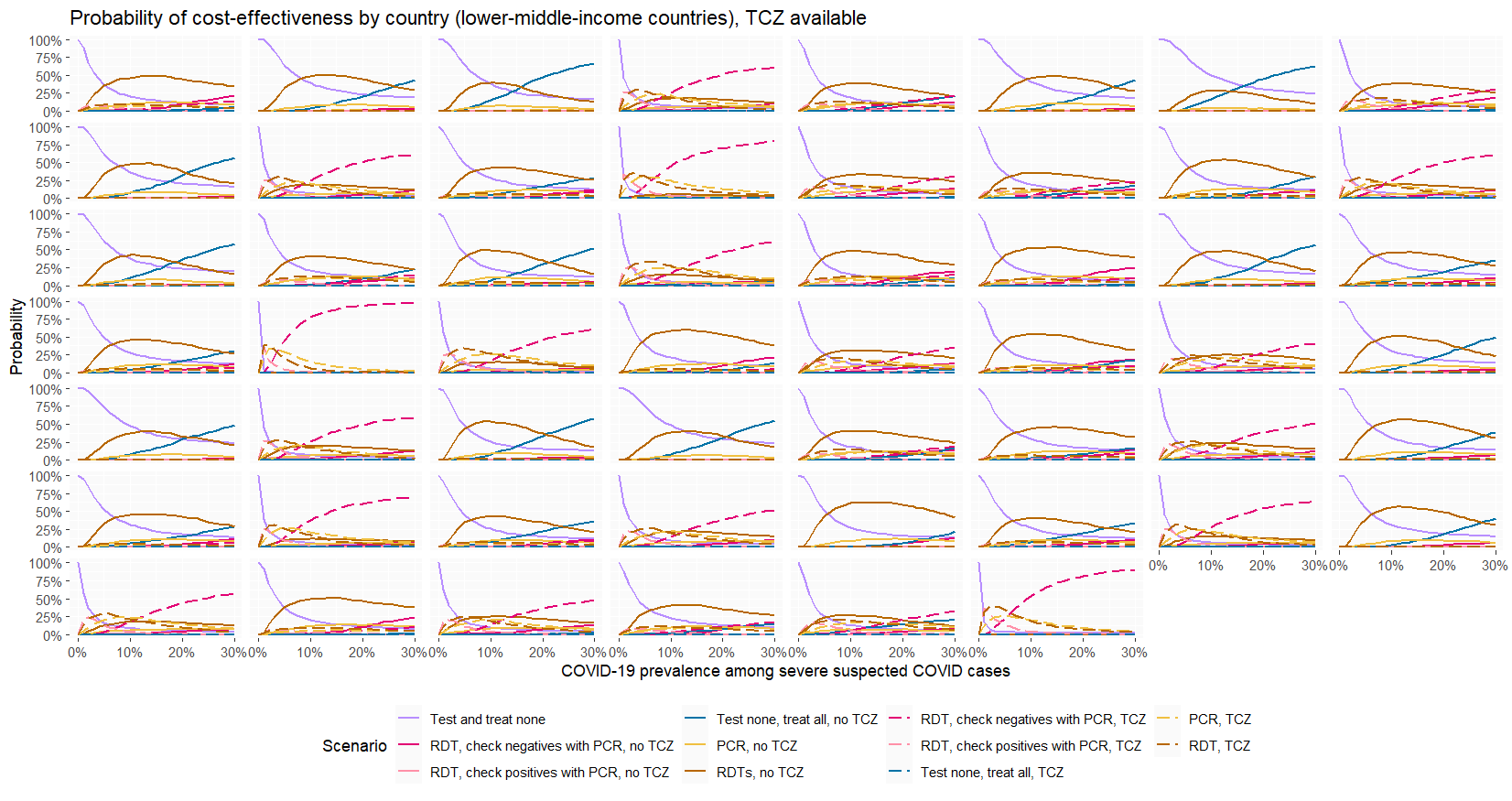


** PCR = polymerase chain reaction, RDT = rapid diagnostic tests, TCZ = tocilizumab. Dotted lines are used to represent options in which TCZ is used, whereas full lines represent options in which it is not.*

**S42 Figure: Probability for each scenario to be the most cost-effective, by country, upper-middle-income countries (TCZ available)**


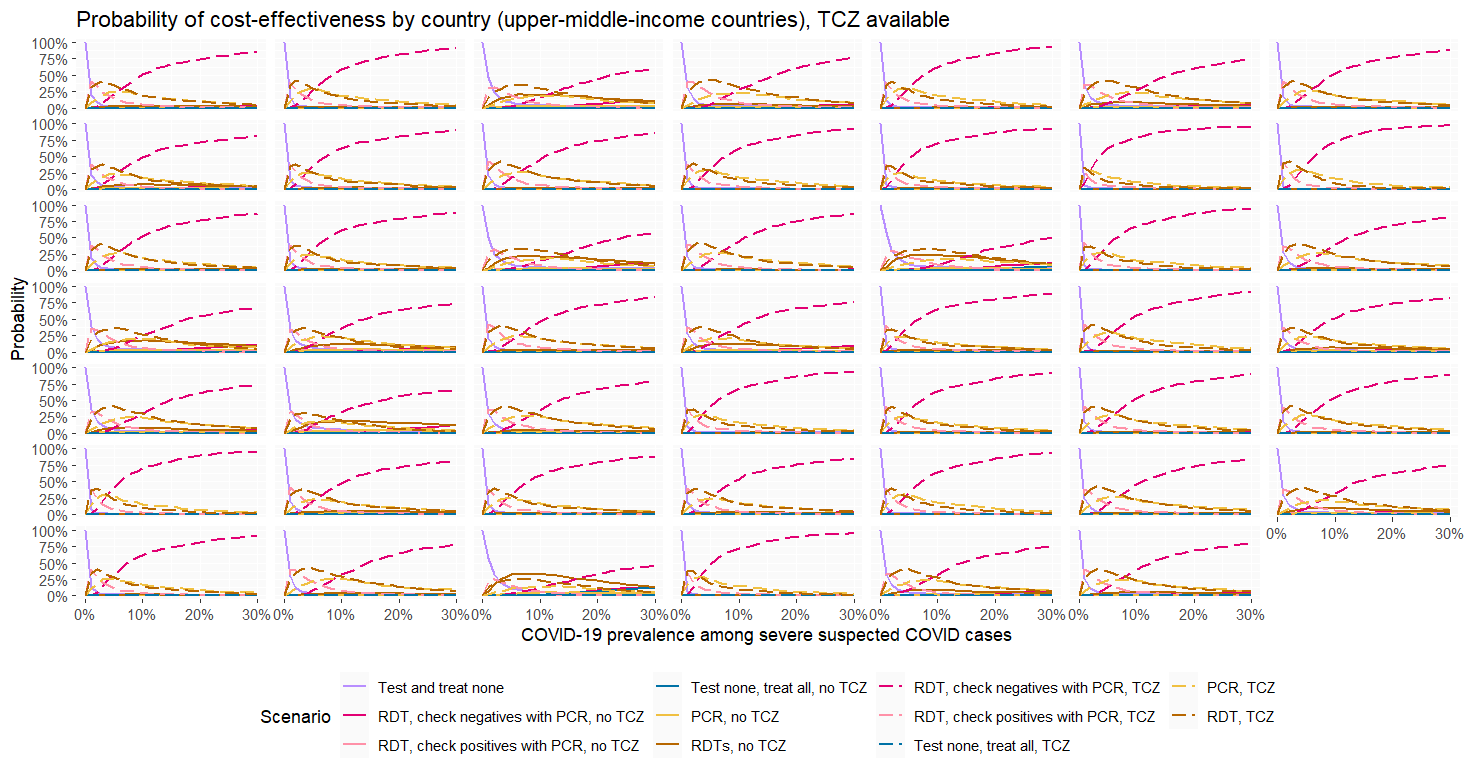


** PCR = polymerase chain reaction, RDT = rapid diagnostic tests, TCZ = tocilizumab. Dotted lines are used to represent options in which TCZ is used, whereas full lines represent options in which it is not.*

**S43 Figure: Probability for each scenario to be the most cost-effective, by country, low-income countries (TCZ unavailable)**


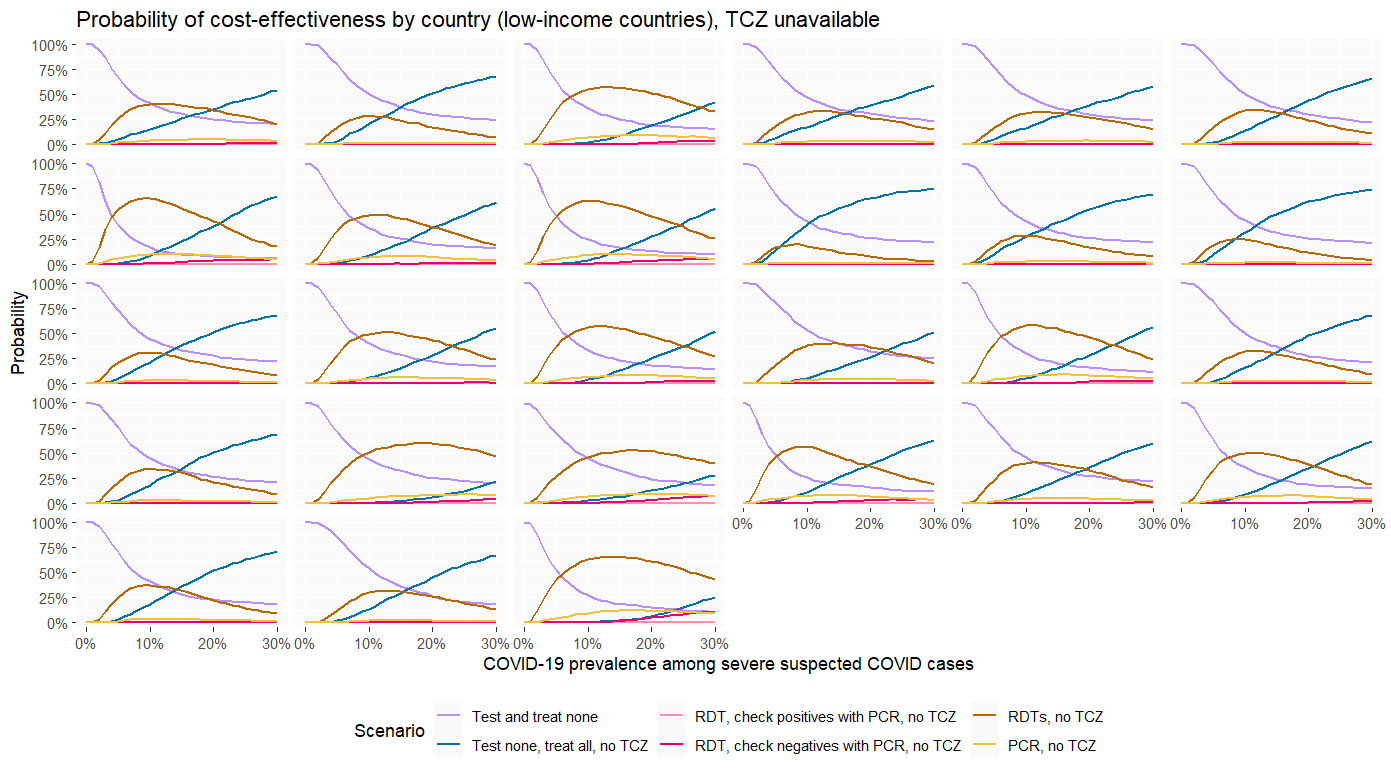


** PCR = polymerase chain reaction, RDT = rapid diagnostic tests, TCZ = tocilizumab*

**S44 Figure: Probability for each scenario to be the most cost-effective, by country, lower-middle-income countries (TCZ unavailable)**


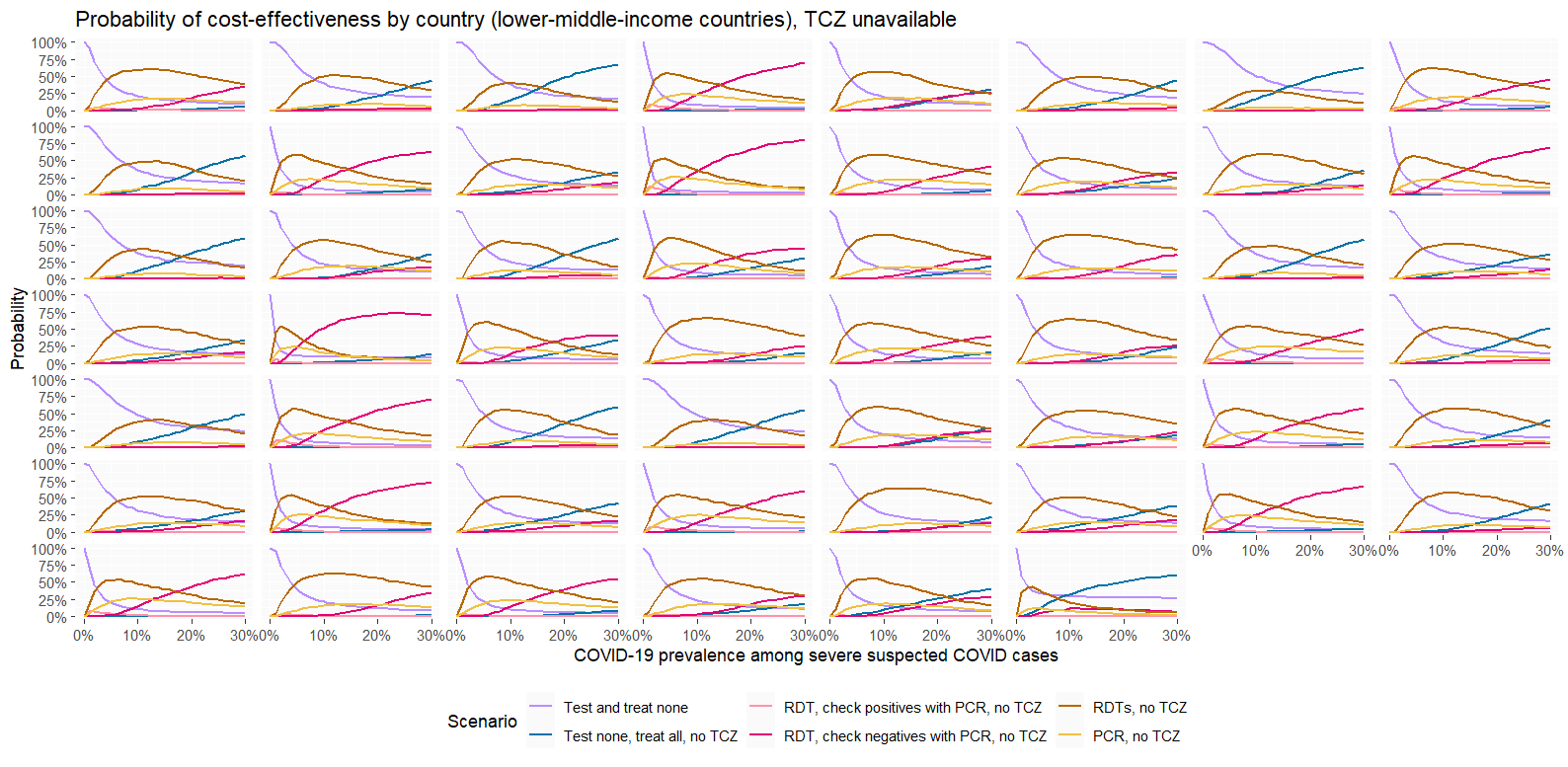


** PCR = polymerase chain reaction, RDT = rapid diagnostic tests, TCZ = tocilizumab*

**S45 Figure: Probability for each scenario to be the most cost-effective, by country, upper-middle-income countries (TCZ unavailable)**


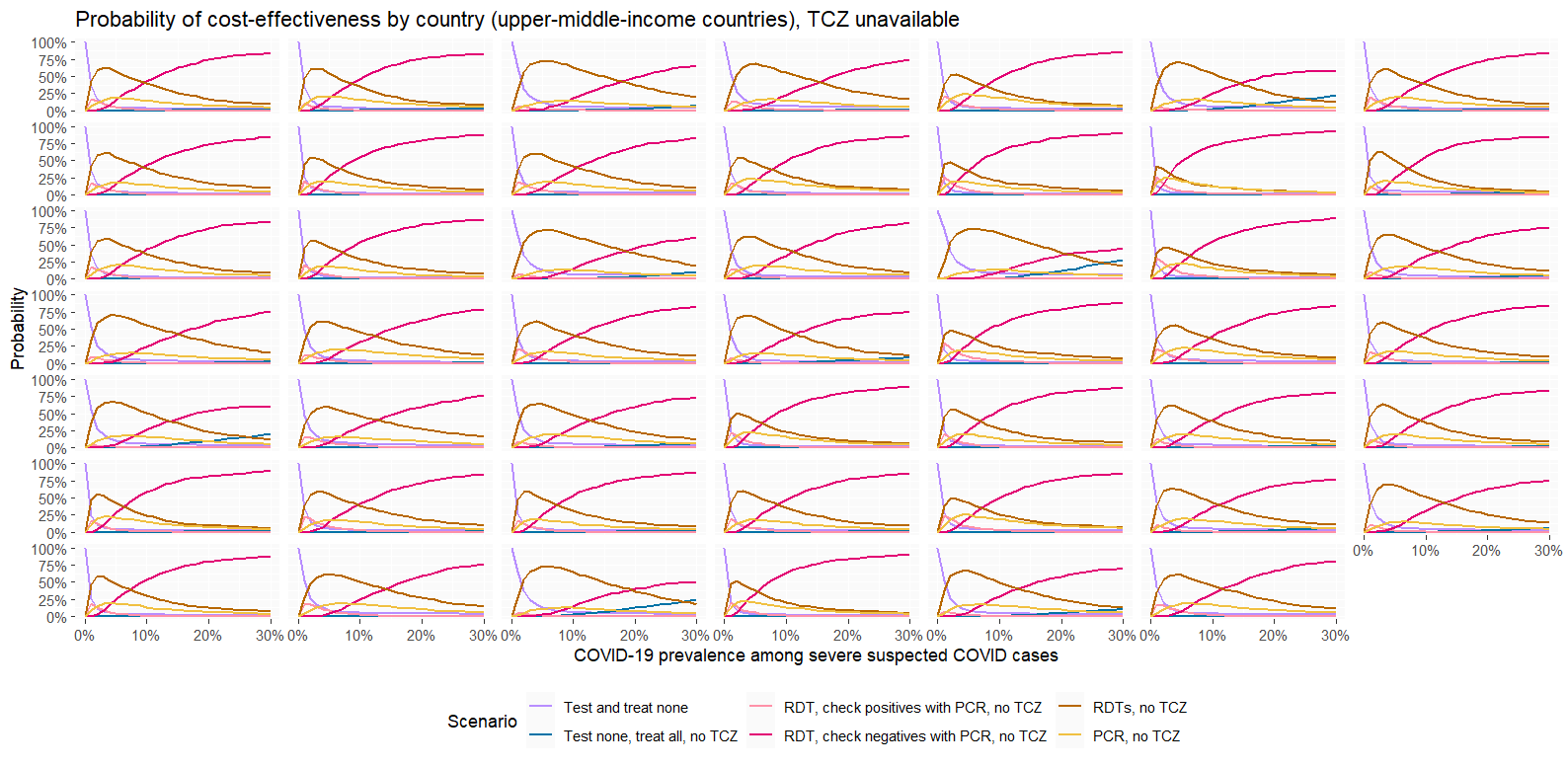


** PCR = polymerase chain reaction, RDT = rapid diagnostic tests, TCZ = tocilizumab*
