# Supplementary material for: Cost-effectiveness of COVID rapid diagnostic tests for patients with severe/critical illness in low- and middle-income countries: A modeling study
Source: PLoS Med. 2024 Jul 18;21(7):e1004429. doi: 10.1371/journal.pmed.1004429 (PMC11293649; doi:10.1371/journal.pmed.1004429)
Supplement: Attachment — Submitted filename: Requests from Editors reply.docx [file pmed.1004429.s006.docx]

Responses to the requests from Editors:

**ABSTRACT**
1) l.54: Please define “PCR” at first use. Done
2) l.69: Please write “IL-6” in full. Done

**AUTHOR SUMMARY**
1) l.85: Please define ‘LMIC’ at first use. Done
2) l.96: Please write ‘RDT’ in full. Done
3) l.98: Please write ‘PCR’ in full. Done

**INTRODUCTION**
l.69: Please define “IL-6” at first use. Done

METHODS AND RESULTS
1) l.139: We suggest that you include a reference here, or where you feel it is appropriate, to the list of countries included. We have added a reference to S2.2 appendix as this appendix provides the names of all included countries and corresponding parameter values.
2) Figure 1: What do the boxes with a dashed outline signify? The boxes indicate the elements that are reproduced elsewhere in the decision tree – see the explanations we have added in this regard in the figure caption.
3) l.228: Please check carefully whether S3.4 Appendix should be referenced here and not S3.3 Appendix. Ideally, both would be referenced, this has been corrected.
4) l.257: Please define “ICU” at first use or write in full. Done
5) Figure 2/3/5: In the figure description, please define “LIC”, “LMIC”, and “UMIC”. Also, we suggest including the numbers (of 129) that were included in the three different country groups (LIC, LMIC, UMIC). Done
6) Figure 4: Since you are displaying percentage points and not percentages, please remove the unit from the y-axis. Done
7) ll.345-351: For better comprehension of the figure, we suggest detailing one example, e.g. test kit costs, with the relevant numbers. For example (assuming we have interpreted the figure and data correctly): If RDT test kits prices were at $6.20, the share of countries in which testing would be cost-effective would decrease by 15 percentage points. On the other hand, if RDT test kit prices were at $0.60, the share of countries in which testing would be cost-effective would increase by 7.5 percentage points. Done
8) ll.350-351: “(see S3.2 Appendix for detailed figures associated with each of the scenarios presented in Fig 4).” – should this be S3.3 Appendix? You are right, this has been corrected.

Please carefully revise the manuscript for referencing the relevant supplementary materials and make sure that at minimum the main appendices are all referenced throughout the main manuscript (i.e. second level: S1.1, S1.2, etc.).We have added references to certain appendices (i.e., the second levels) when relevant, in particular, we have indicated that “The values of model parameters are listed in S2.1 (including details of parameter distributions and sources) and S2.2 (for country-specific parameters) and available in github (<https://github.com/gbn0931/tcov>)” at the start of the section on “Estimates of model parameters and cost-effectiveness”. We have further added a reference to S1.1 appendix on line 132 of the paper (at the end of the prior to last paragraph of the introduction), and quoted S2.8 appendix in the first paragraph of “sensitivity analyses”, in methods.

DISCUSSION
1) Table 1: In the table, you have marked the footnotes as *, **, *** (1,2, 3), whereas in the description you have listed the asterisks as **, ***, **** (2,3,4) - please revise. Please define “TCZ”.

Done

2) Please remove the “Conclusions” subheading. Done, we have added “In conclusion” to the beginning of the corresponding paragraph, though, as we felt this would make the transition with the prior paragraph smoother.

REFERENCES
Where website addresses are cited, when specifying the date of access, please use the word “accessed” instead of “cited” (e.g. [accessed: 10/04/2024]). This has been corrected in the paper and appendix.

SOCIAL MEDIA
To help us extend the reach of your research, please provide any X (formerly known as Twitter) handle(s) that would be appropriate to tag, including your own, your co-authors’, your institution, funder, or lab. Please enter in the submission form any handles you wish to be included when we post about this paper.
You can use this twitter handle: @AnnaVassall

**Comments from Reviewers:**

Reviewer #2: Thank you to the authors for resubmitting this paper.

The authors have sufficiently responded to each of my individual concerns (and look to addressed the concerns of the other reviewers too). The addition of the table with an overarching summary of the primary results is much appreciated, as is the reduction in the CEAC type figure to three panels - hopefully both of these additions will make it easier for the general reader to interpret the results.

Thanks a lot for your response and review of this paper!

Reviewer #3: Fantastic update to this paper and response to our comments and suggestions- well done :).

Thanks a lot for your kind response and reviewing work!

Any attachments provided with reviews can be seen via the following link:

------------------------------------------------------------

General Editorial Requests

1) We ask every co-author listed on the manuscript to fill in a contributing author statement. If any of the co-authors have not filled in the statement, we will remind them to do so when the paper is revised. If all statements are not completed in a timely fashion this could hold up the re-review process. Should there be a problem getting one of your co-authors to fill in a statement we will be in contact. YOU MUST NOT ADD OR REMOVE AUTHORS UNLESS YOU HAVE ALERTED THE EDITOR HANDLING THE MANUSCRIPT TO THE CHANGE AND THEY SPECIFICALLY HAVE AGREED TO IT.

All co-authors have filled in a conflict of interest statement. Please do tell us if they need to fill in any additional document.

2) Please ensure that the paper adheres to the PLOS Data Availability Policy (see [http://journals.plos.org/plosmedicine/s/data-availability](http://url.uk.m.mimecastprotect.com/s/Khp7C3wNDc8N909c2wlMO?domain=journals.plos.org)), which requires that all data underlying the study's findings be provided in a repository or as Supporting Information. For data residing with a third party, authors are required to provide instructions with contact information for obtaining the data. PLOS journals do not allow statements supported by "data not shown" or "unpublished results." For such statements, authors must provide supporting data or cite public sources that include it.

All data are provided in the appendices or in github or available in publicly available sources we referenced.

3) Please review your reference list to ensure that it is complete and correct. If you have cited papers that have been retracted, please include the rationale for doing so in the manuscript text, or remove these references and replace them with relevant current references. Any changes to the reference list should be mentioned in the rebuttal letter that accompanies your revised manuscript.

We have checked all papers cited against <http://retractiondatabase.org/>, and none of them has been retraced.
