# Supplementary material for: Cost-effectiveness of COVID rapid diagnostic tests for patients with severe/critical illness in low- and middle-income countries: A modeling study
Source: PLoS Med. 2024 Jul 18;21(7):e1004429. doi: 10.1371/journal.pmed.1004429 (PMC11293649; doi:10.1371/journal.pmed.1004429)
Supplement: Attachment — Submitted filename: Reviewer comments and feedback 5 May.docx [file pmed.1004429.s005.docx]

In revising the manuscript for further consideration, your revisions should address the specific points made by each reviewer and the editors. Please also check the guidelines for revised papers at [http://journals.plos.org/plosmedicine/s/revising-your-manuscript](http://url.uk.m.mimecastprotect.com/s/4_JJCKQDxUx6ZDjsMAtSd?domain=journals.plos.org) for any that apply to your paper. In your rebuttal letter you should indicate your response to the reviewers' and editors' comments, the changes you have made in the manuscript, and include either an excerpt of the revised text or the location (eg: page and line number) where each change can be found. Please submit a clean version of the paper as the main article file; a version with changes marked should be uploaded as a marked up manuscript.

Please use the following link to submit the revised manuscript: <https://www.editorialmanager.com/pmedicine/>

Your article can be found in the "Submissions Needing Revision" folder.

We expect to receive your revised manuscript by April 5 2024. However, if this deadline is not feasible, please contact me by email, and we can discuss a suitable alternative.

Don't hesitate to contact me directly with any questions ([aschaefer@plos.org](mailto:aschaefer@plos.org)). If you reply directly to this message, please be sure to 'Reply All' so your message comes directly to my inbox.

-----------------------------------------------------------

Requests from the editors (Please note: not all will apply to your paper, but please check each item carefully):

ACADEMIC EDITOR COMMENTS

The focus should be on making the manuscript comprehensible to the majority of readers. The reviewers all strongly recommended this. As I read through the results, I found myself looking for the **summary table that would tell me the outcomes of all the scenarios in simple terms**. I think the reliance on Figures 2 and 3 to convey the most important results might be challenging, as most readers will not understand these figures, or even be inclined to try to decipher them. I also found that I still don't know what the results mean for any actual, individual country or implementer. Specific case study examples would help a lot.

Thank you very much for these comments. We agree and have substantially revised the presentation of our results. We have:

- Reduced the complexity of Figures 2 and 3 for them to include only 3 panels (low-, lower-middle- and upper-middle-income for a medium scenario) rather than 9. The additional scenarios presented in the original panels are now included in the Supplementary Material, so the core message remains clear.
- Clearly summarized policy implications in the discussion section and added a summary table.

We also considered the suggestion to use case studies, but the reason why we did not use country names in the first place (and actually removed them from the final, country-specific graphs in Appendix) was that data was not sufficiently localized to do so, and we would prefer not to mention specific countries in the revised paper if at all acceptable to the reviewers and editors. There is a danger if we do so, that our results would appear more certain to specific contexts than they are. Country stakeholders are advised to start from the estimates for the country income group relevant to them, but also consider the sensitivity analyses that highlight sensitivity to other context-specific determinants of cost-effectiveness.

The comments that struck me as most important to respond to are:

Reviewer 1, second paragraph under "General" heading ("My main concern..."). This is well stated, and I agree entirely.

Reviewer 2, second paragraph ("My first issue...), which reiterates the concerns of Reviewer 1, and the comment: ""Our analysis shows that the most cost-effective way to use RDTs to inform COVID treatment depends on country income, SARS-COV-2 prevalence, and flue prevalence". These are three factors that you picked out to analyse ex ante in this study - are there any other important factors that you weren't able to capture in your analysis that are noteworthy?"

Reviewer 3, "The cost of a test kit is now around ≤$2 (landed price)- how does this alter or streamline the recommendations from this paper?" (I am also interested in the implications for antimicrobial stewardship, but that might be beyond the scope of this manuscript.)

We have replied to each reviewer’s comments and made necessary changes in our manuscript – including adjustment of the cost of test kits in our baseline scenario. See the corresponding sections below.

EDITORIAL COMMENTS

The Editors concur with the Academic Editor that the study needs to be streamlined and the message simplified to be accessible to a broad audience. **Regarding the specific case study examples, we are unsure whether this might create too much of a focus on specific countries and detract from the overall message**. However, if you feel this would strengthen your manuscript while making it more accessible, we leave it up to you to decide whether to include these examples.

Thank you for your comments. As explained above in response to the academic editor, we have tried to simplify the figures and make the message more accessible to a broader audience, in particular by adding a summary table and highlighting the policy implications. We have not used specific case study examples because the data we have used are not sufficiently localized to allow us to do so – this was also our original motivation for not providing specific country examples. Our changes are more extensively detailed under our answers to each specific reviewer.

The Editors also discussed whether your analysis should be **expanded to include broader societal costs/wider economic considerations, including socioeconomic impacts, e.g. through an extended cost-effectiveness analysis (ECEA**). We would be interested to hear your thoughts on this and whether this would be a feasible approach that would be in keeping with the purpose of your study, taking into account the need to make the study more accessible to the average reader.

Thank you for this comment. This is something that we agree could be very interesting and informative as future work. However, we feel that it is outside of the scope of this paper to consider equity and financial protection and that this would require considerable additional data (such as the distribution of health and financial impacts by income/wealth quintile). Moreover, as the manuscript has already been felt by reviewers to be somewhat “too comprehensive”, adding such a substantive piece to the analysis would likely make achieving clarity for an average reader even harder.

We have nevertheless strengthened the conclusions to make the policy implications of our findings clearer.

GENERAL COMMENTS

1) Please cite the reference numbers in square brackets. Citations should be preceding punctuation.

Done, all citations (in the main paper and appendices) are now in square brackets.

2) Please temper claims of primacy of results by stating, "to our knowledge" or something similar.

We have revised the sections where we discuss other results. Mindful of the size of the abstract, we now only make a relatively broad statement there (“*Existing analyses are limited to a narrow set of countries and uses*”). Meanwhile, we have added “*to our knowledge*” in the author summary (third bullet) where we have the statement: “*To our knowledge, no paper has considered both presumptive and symptomatic COVID treatment as alternatives to testing, or has considered a range of treatment options reflective of LMIC contexts”.* We have also added the same caveat in the introduction (second paragraph), where we say: “*To our knowledge, no previous study has considered both presumptive and symptomatic COVID treatment, estimated the unintended effects of treating false positives, or assessed a similar range of treatment options*”.

FINCANCIAL DISCLOSURE

The funding statement should include: specific grant numbers, initials of authors who received each award, URLs to sponsors’ websites. Also, please state whether any sponsors or funders (other than the named authors) played any role in study design, data collection and analysis, the decision to publish, or preparation of the manuscript. If they had no role in the research, include this sentence: “The funders had no role in study design, data collection and analysis, decision to publish, or preparation of the manuscript.”

Done.

COMPETING INTEREST

All authors must declare their relevant competing interests per the PLOS policy, which can be seen here: [https://journals.plos.org/plosmedicine/s/competing-interests](https://url.uk.m.mimecastprotect.com/s/_eHyCM1GzcV7v2QUJQvCb?domain=journals.plos.org). For authors with ties to industry, please indicate whether any of the interests has a financial stake in the results of the current study.

We have asked all authors to fill in conflict of interest forms and are submitting those with the revisions to the paper.

TITLE

Please revise your title according to PLOS Medicine's style. Your title must be nondeclarative and not a question. It should begin with main concept if possible. "Effect of" should be used only if causality can be inferred, i.e., for an RCT. Please place the study design ("A randomized controlled trial," "A retrospective study," "A modelling study," etc.) in the subtitle (ie, after a colon). Please remove “To test or not to test?” from the study title.

“To test or not to test” has been removed from the title, and “a modelling study” added. The revised title is “*Cost-effectiveness of COVID rapid diagnostic tests for severe/critical patients in low-and-middle income countries, a modelling study*”.

ABSTRACT

1. Please structure your abstract using the PLOS Medicine headings (Background, Methods and Findings, Conclusions). Please combine the Methods and Findings sections into one section, “Methods and findings”.

We have revised the abstract accordingly.

2. PLOS Medicine requests that main results are quantified with 95% CIs as well as p values. When reporting p values please report as p<0.001 and where higher as the exact p value p=0.002, for example. For the purposes of transparent data reporting, if not including the aforementioned please clearly state the reasons why not. When a p value is given, please specify the statistical test used to determine it.

We have conducted and present the standard uncertainty analyses as described in economic evaluation reference cases. As the paper covers such a broad range of countries and scenarios, however, most results are qualitative.

3. Throughout, suggest reporting statistical information as follows to improve clarity for the reader “22% (95% CI [13%,28%]; p</=)”. Please be sure to define all numerical values at first use. Please amend throughout the abstract and main manuscript. Please note the use of commas to separate upper and lower bounds, as opposed to hyphens as these can be confused with reporting of negative values.

We have removed hyphens and added comas in all intervals throughout.

4. Please ensure that all numbers presented in the abstract are present and identical to numbers presented in the main manuscript text.

We have checked that.

5. Please include the study design, population and setting, number of participants, years during which the study took place, length of follow up, main outcome measures.

We believe we have done so: this is a modelling study, focusing on low-and-middle-income countries. As this is a modelling study, there are no specific study participants or duration, but we do specify the number of simulations our results are based on. Furthermore, outcome measures are defined in the paper.

6. Please include the important dependent variables that are adjusted for in the analyses.

We have explained which variables were adjusted in the analysis and added “dependent variable” next to the associated description in S2.7 Appendix.

7. Please define all abbreviations including those for statistical reporting at first use.

We have looked through the text and appendix and we believe we have now done so.

8. Abstract Background: The final sentence should clearly state the study question.

We have added the following sentence at the end of the “background” section: “*The aim of this study is to assess the cost-effectiveness of COVID RDTs to inform the treatment of severe patients in LMICs, considering real world practice*.”

9. In the last sentence of the Abstract Methods and Findings section, please describe the main limitation(s) of the study's methodology.

We have added the following sentence on the limitations of the study at the end of the “Methods and Findings” section in the abstract: “*The primary limitation of our analysis is substantial uncertainty around some of the parameters in our model due to limited data, most notably on current COVID mortality with standard of care, and insufficient evidence on the impact of corticosteroids on patients with severe influenza.”*

AUTHOR SUMMARY

At this stage, we ask that you include a short, non-technical Author Summary of your research to make findings accessible to a wide audience that includes both scientists and non-scientists. The authors summary should consist of 2-3 succinct bullet points under each of the following headings:

• Why Was This Study Done? Authors should reflect on what was known about the topic before the research was published and why the research was needed.

• What Did the Researchers Do and Find? Authors should briefly describe the study design that was used and the study’s major findings. Do include the headline numbers from the study, such as the sample size and key findings.

• What Do These Findings Mean? Authors should reflect on the new knowledge generated by the research and the implications for practice, research, policy, or public health. Authors should also consider how the interpretation of the study’s findings may be affected by the study limitations. In the final bullet point of ‘What Do These Findings Mean?’, please describe the main limitations of the study in non-technical language.

Author Summary should immediately follow the Abstract in your revised manuscript. This text is subject to editorial change and should be distinct from the scientific abstract. Please see our author guidelines for more information: [https://journals.plos.org/plosmedicine/s/revising-your-manuscript#loc-author-summary](https://url.uk.m.mimecastprotect.com/s/yc0fCN0JAi5zwZ2fRS6VN?domain=journals.plos.org)

An author summary has now been developed and included in the manuscript, just after the abstract.

METHODS AND RESULTS

1. Please ensure that the study is reported according to the CHEERS guideline and include the completed CHEERS checklist as Supporting Information. Please add the following statement, or similar, to the Methods: "This study is reported as per the Consolidated Health Economic Evaluation Reporting Standards 2022 (CHEERS2022) Statement (S1 Checklist)." When completing the checklist, please use section and paragraph numbers, rather than page numbers.

The CHEERS checklist was already in supporting information. In the main manuscript, we have revised our statement to specify the year of the CHEERS guidelines we are referring to, as follows: “*Study methods are reported as per the Consolidated Health Economic Evaluation Reporting Standards 2022 (CHEERS2022) checklist (S1.1 Appendix)*” (first paragraph of the “Methods” section). Further, in the appendix, we have added “*2022*” to references to the CHEERS checklist and changed page numbers to section/subsection and paragraph numbers (except where there is only one paragraph, in which case, only the section was referred to).

2. PLOS Medicine requests that main results are quantified with 95% CIs as well as p values. We suggest reporting statistical information as detailed above – see under ABSTRACT

We have conducted and present the standard uncertainty analyses as described in economic evaluation reference cases as per the IDSI reference case. As the paper covers such a broad range of countries and scenarios, however, most results are qualitative.

3. Please present numerators and denominators for percentages (at least in the Tables [not necessarily each time they're mentioned]).

Several of the percentages mentioned in the paper (e.g., COVID and flu prevalence or discount rate) are assumptions/scenarios which, as such, do not have numerators or denominators. We reviewed the entire paper (main text and appendices) and added details on numerators/denominators and total sample sizes where relevant and feasible.

DISCUSSION

Please present and organize the Discussion as follows: a short, clear summary of the article's findings; what the study adds to existing research and where and why the results may differ from previous research; strengths and limitations of the study; implications and next steps for research, clinical practice, and/or public policy; one-paragraph conclusion.

We have reviewed the structure of the discussion accordingly, and also added subheadings reflecting the sections above.

FIGURES

For all Figures, please ensure that you have complied with our figures requirements [http://journals.plos.org/plosmedicine/s/figures](http://url.uk.m.mimecastprotect.com/s/j-XHCO8KBCrMEN6IP3exQ?domain=journals.plos.org).

All figures in the manuscript have now been submitted in TIFF formats, in separate files, and checked with the PACE tool.

1) Please provide titles and legends for all figures (including those in Supporting Information files).

All figures have titles, including in supporting information. Most figures have legends (not all of them needed one).

2) Please consider avoiding the use of red and green in order to make your figure more accessible to those with color blindness.

We have adjusted all our graphs, and use a colour-blind-compatible palette, as per the <https://medialab.github.io/iwanthue/> website. We further use line type (dotted vs. full) to mark the difference between scenarios in which only corticosteroids are used on positive cases (full line) and scenarios in which corticosteroid and TCZ are used (dotted lines).

3) Please in the figure legend/description, define abbreviations used in each figure (including those in Supporting Information files).

We have rechecked all the figures and added descriptions defining any abbreviation used in the figures – including in supporting information.

4) Please define the meaning of all dots, lines and bars in the captions/footnotes.

The meaning of the use of all dotted lines vs. full lines and bars now described in captions/footnotes.

TABLES

1) Please provide titles and legends for all tables (including those in Supporting Information files).

All tables have titles. They also have legends whenever relevant.

2) Please define all abbreviations used in the table below each table (including those in Supporting Information files).

Done. All abbreviations are explained below the tables.

SUPPLEMENTARY MATERIAL

1) For supplementary figures and tables, please see the general comments under TABLES and FIGURES (color, abbreviations, titles, descriptions, etc.) and amend accordingly.

Supplementary figures now follow the same colour-blind friendly colour palette as figures in the main papers. They also all have titles and all relevant abbreviation are explained at the bottom of each table or figure.

2) We suggest reporting statistical information as detailed above – see under ABSTRACT. Please be sure to define all numerical values.

We have reviewed the paper looking into numerical values and statistical information we reported. Note that most of our results are qualitative due to the wide range of countries and prevalences considered.

3) As for the main manuscript, please indicate whether analyses are adjusted to help facilitate transparent data reporting please also detail the factors adjusted for and present the unadjusted analyses for comparison. If not, please clearly state the reasons why not.

We believe this comment is not relevant to our paper.

4) Please revise the references in the supplementary material according to comments detailed below – see under REFERENCES.

All the references in the supplementary materials have been revised in the same way as in the main manuscript.

5) Please cite your Supporting Information as outlined here: [https://journals.plos.org/plosmedicine/s/supporting-information](https://url.uk.m.mimecastprotect.com/s/6qc7CPQLDUAPJ07HrwCzd?domain=journals.plos.org)
All supporting information sections, tables and figures now have a title of the form “S1 Appendix” or “S5 figure”, with S and a number preceding the type (figure, table) and title of the corresponding item.

REFERENCES

1) PLOS uses the numbered citation (citation-sequence) method and first six authors, et al.

Done.

2) Please ensure that journal name abbreviations match those found in the National Center for Biotechnology Information (NCBI) databases ([http://www.ncbi.nlm.nih.gov/nlmcatalog/journals](http://url.uk.m.mimecastprotect.com/s/1brhCQ7MET4YJX5C9-UXS?domain=ncbi.nlm.nih.gov)), and are appropriately formatted and capitalised.

We hope the journal names and abbreviations are now appropriate.

3) Where website addresses are cited, please specify the date of access (e.g. [accessed: 12/06/2023]).

Done.

4) Please also see [https://journals.plos.org/plosmedicine/s/submission-guidelines#loc-references](https://url.uk.m.mimecastprotect.com/s/bhbGCR1NGcPxyGAf0EVqt?domain=journals.plos.org) for further details on reference formatting.

Thanks, we have tried to format all references appropriately.

**Comments from the Reviewers**

**Reviewer #1:**

Review: "To test or not to test? A cost-effectiveness analysis of COVID rapid diagnostic tests for severe/critical patients in Low-and-Middle Income Countries" PLOS Medicine 2/6/24

General
The authors present a novel study assessing the cost-effectiveness of COVID RDTs for severe/critical patients in LMIC settings. Using a dizzying array of statistical modeling based on published and/or publicly-available data, the authors provide a tremendous number of "real-world" scenarios involving infection prevalence rates, treatment side effect rates, and medication availability rates (amongst other factors) to inform their analyses. Strong points include how widely representative the author list is, both by geography and by country income level, as well as the intensely comprehensive nature of the analyses. Overall, I believe that this manuscript has intrinsic value and is worthy of publication. I applaud the authors for their clearly hard work.

Thank you for your review and positive comments!

My main concern is that the level of comprehensiveness may detract from the potential impact of the paper. In other words, are the authors trying to accomplish too much all at once and, if so, could they make their same points using fewer subgroup analyses? Is every possible scenario included in this paper necessary? Trying to accomplish everything in one fell swoop creates very dense text and innumerable, highly complex figures, each with multiple variables that require intense concentration to process, that limit the reader's ability to digest the complexity of the analysis in any easy way. The authors would do well by simplifying the analysis (if possible) and limiting the figures—or, perhaps—adding a simple table—that more clearly and easily highlights the most salient findings of their study.

Thank you very much for your comment, which has made us reconsider the presentation of results in the paper. We have:

- Simplified existing graphs in the main paper, by reducing 9-figure panels to 3-figure panels. On reflection, we think that the 9-figure panels actually included multiple scenarios that were best addressed in sensitivity analysis, and removing those from the main analysis makes the paper more readable.
- Added a table to summarize the most cost-effective options at the end.
- Summarized the overall policy implications in the Discussion more clearly.
- Mostly kept the range of scenarios we had originally explored, but moved some of them to the appendices.

Abstract
-The Findings and Interpretation sections, at first read, are confusing as the authors bounce back and forth between mentioning COVID, influenza and steroids. A better organized abstract that explains earlier on how steroids and influenza are relevant to this paper would make it easier to understand.

Thank you very much for this comment. One of the key findings of our study is that the side-effects of COVID treatment (which in many LMICs is only corticosteroids) have a substantial impact on the cost-effectiveness of COVID testing, particularly during peak influenza activity. We have now revised our text to make the scope of the study clearer from the beginning, changing the last sentence of the first paragraph of the “Methods and Findings” section and beginning of the second paragraph to:

“*we assessed both intended (COVID symptom alleviation) and unintended (treatment side-effects) health and economic impacts for each testing scenario. We included the side-effects of corticosteroids, which are often the only available treatment for COVID. Because side-effects depend both on the treatment and the patient’s underlying illness (COVID or COVID-like illnesses, such as influenza), we considered the prevalence of COVID-like illnesses in our analyses”.*

Introduction
-No specific concerns.

Method
-Line 97: "We applied a health provider perspective." This line appears to contradict line 190 and the abstract, which indicates that the authors used "a healthcare payer perspective and net monetary benefit approach." Please clarify.

All our apologies. This was an oversight. We used a health provider perspective and net monetary benefit approach. This has now been corrected in the text.

-Line 105: Please help me to understand how estimates for testing and other factors can be made for 129 LMICs when data for the vast majority of those countries do not exist.

You are right. We do not intend to provide reliable figures for a given individual country – as we explained later in the manuscript, results for individual countries are not sufficiently localized to generate accurate individual country results. We parameterised our model based on extrapolations where individual country data was not available (as in the appendix). We see our results as a starting point to inform decisions at the country level, and countries should consider the overall findings for the country income group plus our sensitivity analyses to specific contextual variables. To make this clearer, we have added a sentence at the end of the “model overview section” “*Due to data limitations, results do not reflect individual country contexts sufficiently hence are not meant to be applied in individual countries without consideration of contextual factors*”.

We also expand on that later (3^rd^ paragraph of the analysis section), as follows: “*We present results indicating for which proportion of countries options are cost-effective for a range of SARS-CoV-2 prevalence. We do this as our results do not reflect country context sufficiently to warrant application without consideration of specific contextual factors. Countries wishing to apply these results should combine our main figures with a careful review of our sensitivity analyses that explore the influence of context specific variables for which we were not able to obtain country level data.*”.

-Lines 181-82: The two population-based surveys cited involve US populations. Are LMIC population-based surveys available?

The first survey involved US populations, the second one (Yao et al.) was done using Taiwanese records. We have looked for similar data sources from LMIC settings however, we did not find any.

Specifically, we undertook a quick review of papers using the search terms: ((short-course or short-term or burst) and (corticosteroids or dexamethasone) and (adverse or side or unintended)) in titles and abstracts in pubmed, excluding papers on inhaled or topical steroids, and those focusing on specific diseases or patient profiles e.g. corticosteroids in multiple sclerosis or transplant patients.

-Lines 182-84: "For COVID years of life lost (YLLs), we used published estimates for 81 countries that we extrapolated to other countries (appendix 2.7)." In reviewing appendix 2.7, it seems that the formula used primarily includes age range-based population estimates. Can the authors provide source information for how/where estimates were obtained for the other countries not included in the 81 published in the study cited?

The estimates for the 81 countries (developed by Arolas et al.) were developed using available age-specific COVID mortality and excess mortality data, and comparing those to life expectancy information. To develop proxies for other LMICs, we used age range-based population estimates from the World Population Prospects and GDP per capita data from the World Bank database. We have now added those two sources to S2.7 Appendix.

-Line 185: "LRTI" and "URTI" should be spelled out for readers not familiar with these terms. Additionally, one could argue that other diseases, such as sepsis with acute respiratory distress syndrome, could lead to "COVID-like" deaths. Can the authors comment on why their analysis only included LRTIs, URTIs and TB?

We have added “*lower respiratory tract infections*” and “*upper respiratory tract infections*” in the text before giving the acronyms in brackets.

Regarding the inclusion of non-respiratory disease syndromes, you are right that many different disease syndromes, including sepsis may, at least for some patients, present as “COVID-like”. When developing this paper, to inform our choices of diseases to include in our calculations, we first consulted with LMIC experts^1^ to understand what diseases were most common in their context that could present with COVID-like symptoms. Influenza, malaria, TB and RSV were the most commonly mentioned diseases. Malaria is the only one of those diseases that does not routinely present with respiratory symptoms and was excluded from our baseline computations. However, mindful that respiratory distress is possible with cerebral malaria^2,3^, we did check how much our results would differ if our choice of diseases had been different (e.g., adding malaria deaths to the computation). The values of YLL per COVID death did not vary substantially, hence we kept the three primary GBD causes we had identified.

We had not considered bacterial or viral causes of sepsis, as this had not been flagged by the LMIC experts, but perhaps we should have done so. The GBD includes only two “sepsis” categories: maternal sepsis and neonatal sepsis. Those two categories correspond on average to around 16.3% of all YLLs and 5.9% of deaths in GBD. Further, 6.2% of cases of sepsis are typically associated with ARDS, with cases with ARDS having approximately four types higher mortality than other cases (<https://www.ncbi.nlm.nih.gov/pmc/articles/PMC3800497/>). Based on that information, we can estimate how much the YLLs lost per death in patients with COVID-like symptoms but not COVID disease would have changed had we included a proportion of sepsis deaths corresponding to the estimated share associated with ARDS. On average, YLLs per COVID-like, non-COVID deaths would have changed by 1.6% in LMICs, with the largest change across all countries being 10%.

We therefore concluded that inclusion of sepsis as one syndrome potentially causing COVID-like symptoms would not have altered our broad conclusions, although it may have affected numerical results slightly. We decided to keep our current methodology but add explanations as to our approach (what other diseases may have been included, why they were not, and how we assessed whether this choice had a large impact on YLLs) in Appendix 2.5.

Results
-Most of table 4 is redundant between the main paper and Appendix 3.3.

We assume you mean Figure 4. It is true that Figure 4 and the figure in Appendix 3.3 looked very much alike. They were not identical as the figure in Appendix 3.3 considered sensitivity analysis when TCZ is available, while Figure 4 focused on settings where corticosteroids are the only treatment available. Considering the reviewer comments suggesting overall simplification, however, we have removed this figure from Appendix 3.3.

-Very complex reporting of results that is often difficult to follow. Please see "general" comment, above.

We have revised our reporting of the results, both in the main text and in the discussion and conclusion, as described above, including by reducing some figures and adding a summary table. We hope that the revised manuscript is easier to read.

Conclusion
-No specific concerns.

**Reviewer #2:**

Thank you to the authors for submitting this clearly important paper. I enjoyed reading it.

Thank you very much for your kind comment!

Whilst I appreciate the (very) impressive scope of the paper and definitely think that it has the potential for publication in this journal, I have some concerns that I personally think would need to be addressed before publication could be considered.

My first issue is the presentation of the results. I am a health economist so I understand the interpretation of the figures (as they are essentially a form of CEAC), however for a lot of readers of this journal these will be difficult to interpret without further explanation. Figures 2 and 5 are also very difficult to read given the amount of panels. I think it is important to present the data in tables somehow as this will be easier for the majority of readers to understand. If you are going to present the results exclusively in figures, I think there needs to be a "base case" of sorts for LIC/LMIC/MIC (i.e. a figure with three panels), with scenario/sub-group analyses (like Figure 2) potentially relegated to the appendix.

Thank you very much for this comment. In line with your suggestions, we have reduced the number of panels and used only figures with 3 panels in the manuscript itself. We have also added a table with the main results of the cost-effectiveness analysis at the end of the paper, hoping this can help the reader.

My second main issue is that given the breadth of countries that you are analysing, it's difficult to really interpret the results. Using the "% of countries in which the scenario is cost effective" isn't the most intuitive metric for me (personally I've never seen this before), as there is massive heterogeneity in the countries you're considering. I personally think this needs to be broken down a bit, for instance using a few selected countries (from a range of geographical regions) as case studies and using "% chance of being cost-effective" rather than "% of countries in which the scenario is cost effective". I think it will be easier to tell a coherent story this way.

We have considered using case studies, however, we had originally decided not to have specific country examples or use specific country names (even in the series of country-specific CEAC graphs in Appendix 4) out of concern that the available data are not sufficiently rich to give results for specific, named countries. See comments also above to reviewer 1.

We understand, however, that the share of countries in which a scenario is cost-effective is not a statistic that most readers will be familiar with. Hence we have considered ways of making reading the paper easier. In this context, we have simplified the graphs by reducing the number of panels and added a table with an overarching summary of the primary results of the analysis.

In similar studies we have conducted where we did not feel confident with individual country estimates, we have tried to synthesise variation across country income groups in other ways as a means of supporting decision makers, for example by presenting a range of ICER values for each group. Based on the reviewers’ comments we considered whether this may work here. We explored the link between GDP per capita and our results to see if there was a clear relationship within country income groups, but that is not clearly the case (although it is a strong determinant, it is more complex than that). So all in all, we think our approach is the best way to convey that, while there are some clear messages for each income group, there is some degree of country level uncertainty. We also highlight that those applying our results should consider the range of factors that drive cost-effectiveness, as illustrated in our sensitivity analyses, and how they may be important in their context. To assist readers further, we have also provided additional figures in S3.1 appendix showing how an individual country’s cost-effectiveness threshold affects which option is the most likely to be cost-effective.

I also have a few smaller points:

Line 105 - To what extent do you think the model you developed can be considered relevant to all 129 LMICs? Which areas of the model do you think are most and least transferable between countries?

The model we developed uses, by nature, a variety of data sources. Some of those are country-specific, while others are only proxies. Hence, we did not aim to develop reliable results for all 129 countries – instead, by running analyses with parameters representing a wide range of countries at each income level, we sought to highlight common trends for countries at a given income level (e.g. when a testing scenario is cost-effective in almost none or almost all of the country simulations). To make this clearer, we have added (also in response to reviewer 1’s comment on the same line): “*Due to data limitations, results do not reflect individual country contexts sufficiently hence are not meant to be applied in individual countries without consideration of contextual factors*” and have also expanded on this issues in the 3^rd^ paragraph of the analysis section.

There are some areas of results that are consistent for all countries in that income range and therefore are likely applicable to individual countries, However, there are key contextual factors that are likely to have variation by country in addition to this, for example influenza prevalence. We have highlighted these in discussion section.

Line 302 - "Our analysis shows that the most cost-effective way to use RDTs to inform COVID treatment depends on country income, SARS-COV-2 prevalence and flue prevalence". These are three factors that you picked out to analyse ex ante in this study - are there any other important factors that you weren't able to capture in your analysis that are noteworthy?

Indeed based on our expert consultation prior to model development and previous literature (ex-ante) we expected heterogeneity between countries (particularly linked to income level) and SARS-CoV-2 prevalence to be important, hence the emphasis we put on those parameters in the paper. We had not expected the role of influenza prevalence to be so pivotal when we started developing the model and this only became apparent when we reviewed corticosteroid side effects. There were no other major factors identified by stakeholders that we did not include in the model structure or consider in parameterisation.

We further detailed several deterministic sensitivity analyses in the main manuscript (focusing on parameters expected to vary between countries but for which we do not have country-specific estimates or only have very poor ones).

We have now also added an appendix, S3.4 appendix, which shows the impact of heterogeneity and uncertainty on all model parameters (based on the distributions detailed in S2 appendix). This section confirmed certain results we had expected ex-ante (such as the high importance of COVID prevalence), but also allowed us to identify, for example, the contribution that uncertainty on baseline mortality risk from severe COVID makes to overall uncertainty, which we had not expected. We have summarized the most salient points of this analysis in the revised section “Other sensitivity analyses” in the main manuscript. For example, we now indicate that: “*The net health benefit of RDTs is sensitive to COVID case-fatality risks, suggesting that variations in the viral variants and/or population immunity could be important drivers of changes in model results.”*

Finally, we have revised the discussion. Here we have sought to make the implications of our results clearer and have also provided more extensive explanations regarding model uncertainty. In particular, we discuss the fact that the impact of treating COVID patients with non-COVID treatments or withdrawing non-COVID treatment (e.g. Tamiflu) from somebody who needs it (if they are erroneously diagnosed as having COVID) is a potentially important factor that we were not able to capture in our analysis. Those factors would likely have increased the cost-effectiveness of more accurate testing algorithms. We were not able to include those factors because of the associated complexity (modelling this would require understanding all the alternative diagnoses a COVID-like patient may receive, beyond COVID, and associated care pathways and impacts on COVID and non-COVID patients). We have now added the following sentence in the discussion to highlight this:

“*Another key simplification is that we have only quantified the impact of treatment for COVID, or its absence, on COVID and non-COVID patients. In doing so, we have ignored the impact of treating a COVID patient erroneously with therapeutics specific to other diseases or of failing to treat a non-COVID patient who tests positive for COVID (false positive) with the appropriate therapeutics. The inclusion of these considerations would likely have increased the impact of testing algorithms that provide more accurate test results.*”

We have not explicitly modelled the impact of delays in obtaining test results, as we could not estimate these delays and their health impacts from our experts and current data. However, we were informed by our experts that these delays have substantially shrunk since the beginning of the pandemic, from too long to inform clinical decision in some contexts, to less than 24 hours today, so this approximation is likely not as impactful as it may have been earlier in the pandemic. We have originally briefly mentioned this, but have now also added a comment in this regard in the discussion, as follows:

“*At the beginning of the pandemic, PCR test results in some contexts were so delayed they could not be used to inform clinical decisions. However, test results are now generally obtained within 24 hours [8], so our approximation is likely more accurate.*”

Line 326 - "Our work has demonstrated the high uncertainty around the optimal algorithm" - this was always going to be the case given the heterogeneity of the countries involved in the study. How heterogenous were the results within each type of country (LIC, LMIC, MIC) and geographical region?

The largest heterogeneity in outcomes (i.e., which option is the most cost-effective) was found among lower-middle-income countries (see e.g. Table 1 and S3.1 appendix). This makes discussing results for lower-middle-income countries trickier than for low-income or upper-middle-income contexts. We have therefore added graphs in S3.1 Appendix showing how a country’s cost-effectiveness threshold influences what scenario is the most cost-effective. We do not present those in detail in the main manuscript to avoid further adding to the complexity, but do mention some of the headline results (e.g., change in the most cost-effective option for LMICs with a cost-effectiveness threshold above a certain value) in the summary table in the main text.

Discussion
Are there any specific limitations of the model structure that you think it would be useful to mention in the discussion? What simplifications do you think will have the largest impact on the results?

There are limitations to the model due to precision on parameter values, which we have mentioned. Regarding the scope of the exercise, we have taken a provider perspective and therefore do not include indirect impacts of testing. We have also not included the impact of treatments for non-COVID diseases (if given to COVID patients or not given to patients needing them) on economic costs. We have also not included the impact of given these unnecessary treatments on a patient’s health or the impact of delays in obtaining PCR test results. We have now added a discussion of these limitations.

**Reviewer #3**:

Overall, this was a very thorough analysis of the potential cost-effectiveness of the use of RDTs for possible severe cases of COVID in LMICs.

Thank you very much for your kind comments and insightful suggestions!

While the context dependency is underscored, I wonder how the main messages could be further distilled for policy and implementation (e.g. it might not be feasible to implement an approach that changes depending on where we are in an epidemic)

Point well taken. We have revised the presentation of results and policy implications to increase clarity and also address you concerns around practical implementation. We have added the statement “*Our analysis shows that COVID testing among patients with severe, COVID-like illness can be cost-effective in all LMICs depending on SARS-CoV-2 and influenza prevalence among COVID-like patients*” at the beginning of the discussion and a similar one in the abstract. We have also added a summary table at the end of the results section on the cost-effectiveness of testing in contexts in which only corticosteroids are available. We have finally revised our summary of results and their implications in the discussion.

Because we agree that it can be difficult to implement an approach that changes depending on where we are in the epidemic, we also revised the discussion accordingly. In particular, we propose an alternative perspective, based on our earlier discussion with LMIC colleagues. Indeed, some of them had suggested that clinicians differentiated their practice depending on a patient’s “index of suspicion” for COVID (e.g., confirming negative test results with PCR when the initial “index of suspicion” for COVID was high), taking into account for a patient’s symptoms and the local epidemiology. While this is somewhat subjective, to support the interpretation of results by those in clinical practice, we have therefore added the following text:

*“SARS-CoV-2 prevalence varies over time, so developing universal testing policies based on prevalence may be difficult. However, our expert consultation [8] suggests that clinicians are likely to consider local SARS-CoV-2 prevalence at a given time, and combine this with clinical symptoms to determine the “index of suspicion” for a patient. Given this, one way to consider prevalence is for countries to define clinical screening algorithms that are more specific than commonly used algorithms (RDTs alone or RDTs with confirmation of negative results with PCR) hence lead to the identification of patients with a higher index of suspicion, particularly in low-income settings where testing is only cost-effective for such patients.*

*With this in mind, our findings imply that in low-income countries, suspected cases with a low index of suspicion may be treated as non-COVID, whereas those with a high index of suspicion should be tested with RDTs to inform treatment decision.*

*In lower-middle-income countries, only patients with a much lower index of suspicion should be treated as non-COVID, and all other suspected cases should be tested. In the better resourced of those countries, when the index of suspicion (pre-test) is high, patients’ negative RDT results may be confirmed with PCR, whereas when the index of suspicion is low and influenza prevalence very high, positive RDT results may be confirmed with PCR.*

*Finally, in upper-middle-income countries, all suspected cases should be tested and those with a high index of suspicion (pre-test) and a negative RDT result confirmed with PCR. Furthermore, during influenza outbreaks, positive test results in a patient with a low index of suspicion for COVID (pre-test) should be confirmed with PCR.”*

Line 142: "…assumed that antimicrobials are sometimes given to severe patients" - but this may also sometimes be indicated, as secondary bacterial infections are not rare.

This is accurate. We did not assume that antimicrobial provision would automatically be counter-indicated and did not include a health impact associated with this antimicrobial use. We only, more modestly, quantified antimicrobial use in different contexts, based on expert consultations^6^, during which the likelihood of antibiotic provision for patients with COVID-like symptoms and no test result, a positive test result, or a negative test result, was discussed. It was difficult to get a good sense of the level of antibiotic use, particularly as, on the one hand, antibiotics may be indicated to address secondary bacterial infections and, on the other hand, over-prescription of antibiotics remains common in some LMICs. Some agreement was reached that, in most countries, there would be some decline in antibiotic prescription following a positive test result (as compared to a negative result or no result), with a very broad uncertainty range. We have included this estimate in our paper for completeness though, should researchers want to focus on antibiotic prescription, for example to study the implications of COVID testing for antimicrobial resistance, more precision would be needed.

Regarding the wording of the manuscript itself, we are proposing to edit the text in response to your comment, adding a precision in bracket, as follows: “*Finally, we assumed that antibiotics are sometimes given to severe patients, for example to address secondary bacterial infections) with their level of use varying based on test results [8]”.*

Line 149: Figure 2: Can the authors elaborate on the choice in the decision tree to have no further actions if a case is 'clinical screening positive' but does not receive a test? I'm sure that if there is no test available, a clinical diagnosis (albeit with poorer sensitivity/specificity than an RDT) would be made and possibly treated (especially if severe). How is this reflected in the model.

Thank you for this comment. We assume you are talking of figure 1 (which is the decision tree and is on line 149). We realize that in finalizing our figure for submission we failed to include the details of what happens when tests are not available and clinical screening is positive. This was our mistake. We have now revised Figure 1 to accurately reflect our decision tree. Patients that screen positive may, in our model, either be treated as non-COVID (i.e., hospitalized and treated for their symptoms including oxygen supplementation as per need) or treated presumptively for COVID (which means they may, in addition, receive corticosteroids and/or tocilizumab).

We have not included something called “clinical diagnosis” per se in the paper but the LMIC experts we consulted did mention patients with a “high index of suspicion”, which we discuss in the revised paper. Identification of patients with a “high index of suspicion” may be done at the level of the individual clinician or the country may decide to define a screening algorithm that is far more specific than those usually used. We have accordingly added the following paragraph in the discussion (integrating several of your and other reviewers’ comments): “*our expert consultation [8] suggests that clinicians are likely to consider local SARS-CoV-2 prevalence at a given time, and combine this with clinical symptoms to determine the “index of suspicion” for a patient. Given this, one way to consider prevalence is for countries to define clinical screening algorithms that are more specific than commonly used algorithms (RDTs alone or RDTs with confirmation of negative results with PCR) hence lead to the identification of patients with a higher index of suspicion, particularly in low-income settings where testing is only cost-effective for such patients.”*

Finally, please note that we have also adjusted, for clarity, the text describing the different options in the decision tree, in the “model overview” section:

- We adjusted “*In contexts where both RDTs and PCRs are available, we compared five testing options*” by adding “*for patients that are suspected of having COVID based on clinical assessment*” at the end.
- We have also adjusted “*if suspected cases are not tested,* *they may either be treated as not having COVID (receiving O_2_/MV but not corticosteroids/TCZ), or as if they all had COVID (in which case they receive corticosteroids and/or TCZ presumptively)”*. We now write: “*we assume untested suspect cases may either be (i) treated as not having COVID (receiving O2/MV but not corticosteroids/TCZ), in which case incoming patients would still undergo clinical assessment for other diseases but not for COVID, or (ii) treated as having COVID (receiving corticosteroids and/or TCZ presumptively as in the scenarios above), in which case clinical assessment of COVID severity would be needed”.*
- Further, in the footnote under Figure 1, we have adjusted “*(4) includes scenarios that may be chosen in the absence of clinical screening*” by adding “*for COVID*” after “*clinical screening*”.

Line 149: is there any empiric treatment that should be included? (even if a test is negative, some proportion of individuals may be treated with a COVID-related protocol anyway)

Good point!

First of all, just to clarify, we have assumed that severe negative patients (including true and false negatives) would be given at least basic treatment aligning with the symptoms they have: for example, oxygen therapy or, for the most severe cases, mechanical ventilation where available, so nobody is left entirely untreated. What a COVID assessment may change relates to the provision of drugs such as corticosteroids and IL-6 receptor blockers.

Second, you allude to the possibility of treating negative patients with a COVID-related protocol anyway. In the consultations with LMIC experts that formed the basis for the decision tree and care pathways described in this paper, we discussed that point. The feedback was that in some cases severe patients with symptoms strongly evocative of COVID would indeed still be treated as if they had COVID, at least while waiting for them to receive confirmatory testing or for their symptoms to evolve. However, we were not able to elicit any quantification of that phenomenon, hence we did not include it in the model. Inclusion of this phenomenon in the model (even in a simple manner) would have required a rough estimate both of the proportion that would be treated anyway and of the actual probability that those that are treated despite a negative test result actually may have COVID. It is indeed likely that those are the patients most likely to be false negatives, as they are the ones with the most evocative symptoms.

To highlight this point, we have now added a sentence in the discussion, as follows: “*Some clinicians may also test but ignore the test results: we have ignored this phenomenon because of difficulties in quantifying the factors that enter into such decisions.*”

Line 149: how are false positives and false negatives considered in this decision tree? I imagine if a false positive, then outcomes might differ than a true positive. How is this captured?

False positives and false negatives are captured in the decision tree, at least in part. We captured the health impacts of giving COVID drugs to people who do not have COVID – both considering the side-effects similarly likely to happen in COVID and non-COVID patients (for example, nausea or vomiting, or muscle weakness, etc.), and considering the side effects that depend on the actual disease the patient has (e.g. treating patients with influenza using corticosteroids has been flagged by WHO and others as potentially increasing mortality rates). As for false negatives, we capture the impact of them not receiving COVID-specific treatment drugs, based on the results of different trials on the impact of COVID medicines.

What we did not capture is the impact on false negatives of being given, say, oseltamivir because they are thought to have influenza. Doing that would have required 1) to capture what are the most likely alternative diagnoses (which we have done, in part) 2) what treatments are likely to be given in different contexts (not all countries have the same practices) 3) what side effects are associated with those treatment in somebody without severe flu. Similarly, we did not capture the impact on false positives of not being given, say, oseltamivir, for the same reason. A fully comprehensive investigation would have even required understanding the rates of accurate vs. false diagnosis for alternative diagnoses in the different countries, which is largely beyond anything we could have done.

We mention this point in the revised discussion section, as follows: *“Another key simplification is that we have only quantified the impact of treatment for COVID, or its absence, on COVID and non-COVID patients. In doing so, we have ignored the impact of treating a COVID patient erroneously with therapeutics specific to other diseases or of failing to treat a non-COVID patient who tests positive for COVID (false positive) with the appropriate therapeutics. The inclusion of these considerations would likely have increased the impact of testing algorithms that provide more accurate test results.”*

Line 153: the impact on antibiotic prescriptions might be interesting in terms of impact on antimicrobial stewardship (the likelihood of over/under prescription of antibiotics as a consequence of the use of testing to guide treatment rather than clinical diagnosis).

Thank you for the suggestion. When we started this work, we had initially considered modelling this, and this is largely why the model still includes some consideration of the quantity and cost of antimicrobials that are provided to patients. However, consultation with the experts that are also co-authors to this paper^1^ has suggested that knowledge of the implications of testing on the use of antibiotics is very limited. The broad ranges we have used in this paper come from our consultation. They are very imprecise and do not include information on what proportion of those antibiotics are prescribed correctly. As such we did not feel we had enough information to undertake an analysis focusing specifically on antibiotic stewardship.

Line 190: To which prices was the discounting rate applied to?

Many costs do not need discounting (e.g., hospital bed days) because spending is mostly immediate. However, the discounting rate applies to some of the side-effects of corticosteroids or tocilizumab that have long-term implications e.g., the rare but severe impact of increased risk of heart failure. Though we did not apply the discounting ourselves, we made sure that the cost information we used had already been discounted (with a 3% discounting rate).

We have now adjusted the text of our paper to specify which costs are discounted, as follows: “*using a 3% discounting rate for the costs of treatments side-effects lasting more than one year*”.

Line 199: "… so we made broad assumptions based on the likely balance between these different types of costs." What type of approach was taken for this estimation? After reviewing the appendix, it seems like an ingredients-based costing approach. Is that an accurate summary? (If so, I would call it that, rather than broad assumptions)

We have now changed our wording to “*We used an ingredient based costing approach, with estimates of the average cost of each input category made from reviewing secondary data sources, to estimate the cost of treatment side effects (S2.4 Appendix)”*.

Line 237: There are many different scenarios, what would be your recommendations to policy as a consequence of all of this analysis? Also, the likelihood that a policy can effectively be implemented based on underlying prevalence of disease is low- what would be your overall recommendation?

Thank you for this comment! As explained above, we have added the statement “*Our analysis shows that COVID testing among patients with severe, COVID-like illness can be cost-effective in all LMICs depending on SARS-CoV-2 and influenza prevalence among COVID-like patients*” at the beginning of the discussion and a similar one in the abstract. We have added a summary table in results and revised our summary of results and policy implications in the discussion. We have also reflected on the difficulty of implementing a policy that changes with COVID prevalence, as described in response to your comment above. We suggest that, in practice, countries may define clinical screening algorithms that are more specific than commonly used algorithms hence leading to the identification of patients with higher likelihood of having COVID. This would be particularly useful in low-income settings where testing is only cost-effective for such patients.

Line 256: how would the countries know what the prevalence of COVID would be without some type of underlying testing strategy? (current surveillance systems are still not set up for this)

Good point. The point of the paper is not that countries should find out which exact scenario applies to them but instead should look at the range of possibilities (including results of the sensitivity analysis) for their income group, and make decisions based on that range and other factors like their own judgement, feasibility, political factors, etc. We have reflected this within the section on “policy implications” in the discussion, as follows: “*Country decision-makers may consider results for the country income group relevant to them, variability within this group as well as the results of the sensitivity analyses, alongside other factors such as any locally-relevant research, their own judgement, feasibility or political factors to inform local decision-making”.* While we agree that good COVID (or ARI) surveillance would help with this, it is beyond the scope of our paper to make recommendations about surveillance. We have accordingly added sentences to the discussion. These include: “*it is important to note that the most cost-effective option is sensitive to influenza prevalence, which may not be well known in countries without laboratory-based acute respiratory infection surveillance*” and “*Some level of testing for surveillance may be useful to inform this, but it is beyond the scope of our study to make specific recommendations about surveillance.*”

Line 269: Figure 4: I dont understand the +'s in the figure- what exactly do they mean? (e.g. 30% refusal + 60% refusal?)

The “+” was simply a marker because in some cases we had explored two options: 30% and 60% refusal, hence had two data points below the x-axis, and decided to represent the first one with a bar, and the second one with a “+”. The fact that this was not apparent to you suggests that this approach was not clear enough and we have simplified/clarified the figure while revising this paper: in particular, we present fewer option in this figure, which helps improve the visualization.

Line 321: The cost of a test kit is now around ≤$2 (landed price)- how does this alter or streamline the recommendations from this paper?

This is a good question. The Global Fund’s^4^ stated test kit prices range from $0.6-2.25. In this context, we decided to adjust the medium price for COVID test kits in our analysis in line with Global Fund prices ($2 instead of $3.9), as well as the lower boundary for our range ($0.6 instead of $1.6). However, we are uncertain whether all countries would access those kits or whether prices are always below $2.25 (the literature review and expert discussions we had around mid-2023 suggested that, at least at the time, this was not the case). We have therefore elected to keep the upper limit of the price range used in sensitivity analysis.

**Reviewer #4:**

Very nice paper. The only comment I have is that the legends appear to be cut off on some of the figures but I am not sure whether that is an issue with the compiled document. Please check this.

Thank you very much for your comment. Figures in our manuscript were not cut off, so we assume this was an issue with the compiled document rather than the original. However, figures are now submitted as separate files, which hopefully will solve the problem on your end.

Any attachments provided with reviews can be seen via the following link:

-----------------------------------------------------------

General editorial requests:

1) Please ensure that the paper adheres to the PLOS Data Availability Policy (see [http://journals.plos.org/plosmedicine/s/data-availability](http://url.uk.m.mimecastprotect.com/s/fbb1CVQRMUjYz2DcrYiUM?domain=journals.plos.org)), which requires that all data underlying the study's findings be provided in a repository or as Supporting Information. For data residing with a third party, authors are required to provide instructions with contact information for obtaining the data. PLOS journals do not allow statements supported by "data not shown" or "unpublished results." For such statements, authors must provide supporting data or cite public sources that include it.

We have provided the code of the model in gitub and the data in appendix and in github. To make this clearer, we have added “*alongside associated datasets”* in the sentence: “*The model was coded in R version 4.2.2 and is available (alongside associated datasets) at* [*https://github.com/gbn0931/tcov*](https://github.com/gbn0931/tcov)”

2) In addition, we request that you upload any figures associated with your paper as individual TIF or EPS files with 300dpi resolution at resubmission; please read our figure guidelines for more information on our requirements: [http://journals.plos.org/plosmedicine/s/figures](http://url.uk.m.mimecastprotect.com/s/j-XHCO8KBCrMEN6IP3exQ?domain=journals.plos.org). While revising your submission, please upload your figure files to the PACE digital diagnostic tool, [https://pacev2.apexcovantage.com/](https://url.uk.m.mimecastprotect.com/s/sKZtCWqVNfwVN6AHBdWpf?domain=pacev2.apexcovantage.com). PACE helps ensure that figures meet PLOS requirements. To use PACE, you must first register as a user. Then, login and navigate to the UPLOAD tab, where you will find detailed instructions on how to use the tool. If you encounter any issues or have any questions when using PACE, please email us at [PLOSMedicine@plos.org](mailto:PLOSMedicine@plos.org).

We have checked all the figures in our manuscript with PACE, and have uploaded them as separate TIF files.

3) We ask every co-author listed on the manuscript to fill in a contributing author statement, making sure to declare all competing interests. If any of the co-authors have not filled in the statement, we will remind them to do so when the paper is revised. If all statements are not completed in a timely fashion this could hold up the re-review process. If new competing interests are declared later in the revision process, this may also hold up the submission. Should there be a problem getting one of your co-authors to fill in a statement we will be in contact.  YOU MUST NOT ADD OR REMOVE AUTHORS UNLESS YOU HAVE ALERTED THE EDITOR HANDLING THE MANUSCRIPT TO THE CHANGE AND THEY SPECIFICALLY HAVE AGREED TO IT. You can see our competing interests policy here: [http://journals.plos.org/plosmedicine/s/competing-interests](http://url.uk.m.mimecastprotect.com/s/Ljs2CX5WOI7Wk4xtxAEba?domain=journals.plos.org).

All co-authors have now filled in a conflict of interest form that we have provided with the revised submission.

Reference:

1.	Bonnet G, Bimba J, Chavula C, et al. “We usually see a lot of delay in terms of coming for or seeking care”: an expert consultation on COVID testing and care pathways in seven low- and middle-income countries. *BMC Health Serv Res*. 2023;23(1):1288. doi:10.1186/s12913-023-10305-0

2.	Orimadegun A, Ogunbosi B, Orimadegun B. Hypoxemia predicts death from severe falciparum malaria among children under 5 years of age in Nigeria: the need for pulse oximetry in case management. *Afr Health Sci*. 2014;14(2):397-407. doi:10.4314/ahs.v14i2.16

3.	Cabrales P, Martins YC, Ong PK, Zanini GM, Frangos JA, Carvalho LJ. Cerebral tissue oxygenation impairment during experimental cerebral malaria. *Virulence*. 2013;4(8):686-697. doi:10.4161/viru.26348

4.	The Global Fund. *Pooled Procurement Mechanism Reference Pricing: COVID-19 Diagnostics*. Accessed March 15, 2024. https://www.theglobalfund.org/media/10233/covid19_diagnosticsreferenceprices_table_en.pdf
